# Covalent assembly of nanoparticles as a peptidase-degradable platform for molecular MRI

**DOI:** 10.1038/ncomms14254

**Published:** 2017-02-15

**Authors:** Francisco Perez-Balderas, Sander I. van Kasteren, Alaa A. A. Aljabali, Kim Wals, Sébastien Serres, Andrew Jefferson, Manuel Sarmiento Soto, Alexandre A. Khrapitchev, James R Larkin, Claire Bristow, Seung Seo Lee, Guillaume Bort, Filippo De Simone, Sandra J. Campbell, Robin P. Choudhury, Daniel C. Anthony, Nicola R. Sibson, Benjamin G. Davis

**Affiliations:** 1 Department of Oncology, Cancer Research UK & Medical Research Council Oxford Institute for Radiation Oncology, University of Oxford, Oxford OX3 7DQ, UK; 2 Department of Chemistry, Chemistry Research Laboratory, University of Oxford, Oxford OX1 3TA, UK; 3 Department of Cardiovascular Medicine and Oxford Acute Vascular Imaging Centre, University of Oxford, John Radcliffe Hospital, Oxford OX3 9DU, UK; 4 Department of Pharmacology, University of Oxford, Oxford OX1 3QT, UK

## Abstract

Ligand-conjugated microparticles of iron oxide (MPIO) have the potential to provide high sensitivity contrast for molecular magnetic resonance imaging (MRI). However, the accumulation and persistence of non-biodegradable micron-sized particles in liver and spleen precludes their clinical use and limits the translational potential of MPIO-based contrast agents. Here we show that ligand-targeted MPIO derived from multiple iron oxide nanoparticles may be coupled covalently through peptide linkers that are designed to be cleaved by intracellular macrophage proteases. The synthesized particles possess potential characteristics for targeted MRI contrast agents, including high relaxivity, unappreciable sedimentation, clearance from circulation and no overt toxicity. Importantly, we demonstrate that these particles are rapidly degraded both *in vitro* and *in vivo*, and that the targeted probes can be used for detection of inflammation *in vivo* using MRI. This approach provides a platform for molecular MRI contrast agents that is potentially more suitable for translation to humans.

## Abstract (web summary)

Iron oxide microparticles (MPIO) are better MRI contrast agents than nanoparticles, but are of limited clinical use as they are not degradable and so risk toxicity. Here the authors present an iron oxide microparticle MRI contrast agent with peptide linkers that enable degradation into non-toxic nanoparticles *in vivo*.

The high magnetization and low toxicity of superparamagnetic iron oxide particles has led to their widespread use in biomedical and biological applications^1^,^2^,^3^, including MRI^4^,^5^, cancer therapy^6^, cell labelling^7^, biomolecule sensing^8^,^9^ and multimodal imaging^10^. These particles can be synthesized across a range of diameters, of which the ultrasmall superparamagnetic particles of iron oxide^5^ (USPIO; 20–50 nm in diameter) have been clinically used the most. The long circulation half-life of USPIO precludes rapid molecular imaging of target-specific binding owing to high background levels^11^. In contrast, microparticles of iron oxide (MPIO) have a short half-life (<5 min (ref. 12)) in the circulation and greater contrast-to-noise per particle than USPIO owing to their higher iron content (0.26 pg iron per particle versus 1.1 × 10^−6^ pg iron per particle). Moreover, the increased surface area of MPIO (*ca*. 2–12 μm^2^) compared with USPIO (*ca*. 0.005–0.03 μm^2^) enables greater ligand valency, which can substantially increase binding affinities through multivalent effects^13^,^14^. In this context, the strong correlation between target quantification in tissue and contrast volume on non-invasive MRI points strongly towards the advantages of MPIOs^15^. The potential of MPIO for imaging diagnostically useful endovascular cellular events, such as inflammation and activated platelet adhesion, has already been highlighted in a range of animal models of important human disease, including multiple sclerosis^16^,^17^, thrombosis^18^, atherosclerosis^19^, renal^15^ and cerebral ischaemia^16^,^20^, cerebral malaria^21^ and brain metastases^22^. If such MPIO could be synthesized in a biodegradable form, they have the potential to provide superior capabilities for molecular imaging of disease in humans (see Supplementary Note 1).

Although regulatory approval must of course be considered on a case-by-case basis, we consider that translation to clinical use requires a number of essential attributes: biocompatibility, high iron content, controlled biodegradability, functionalizable surface with multivalent capacity, appropriate shape to promote multivalent binding to the planar endothelial surface, short blood half-life, minimal non-specific accumulation and low tendency to agglomerate. Here, we describe a microparticle system designed through covalent assembly of multiple iron oxide nanoparticles (NPs), which combines the advantages of the micron-size iron particle range with biodegradability via the endogenous clearance^23^,^24^,^25^ and degradation systems of the body (Fig. 1, Supplementary Fig. 1). These microparticles are therefore deserving of further research for their potentially enhanced utility as *in vivo* imaging agents and potential clinical application as iron-based molecular MRI contrast agents.

## Results

### mMPIO construction via iron oxide NP conjugation

**Figure 2:**
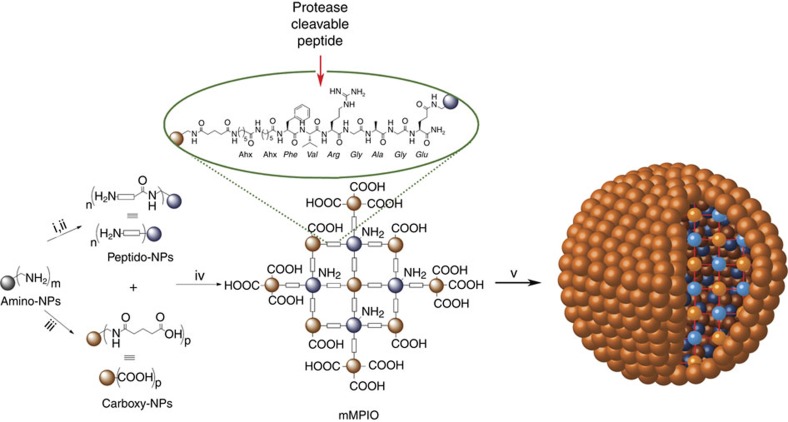
Schematic representation of the synthesis of mMPIO. Grey spheres represent amine-functionalized dextran-coated iron oxide nanoparticles, blue spheres represent peptido-NPs and brown spheres represent carboxy-NPs. Conditions: (i) peptide **3**, *N*-(3-dimethylaminopropyl)-*N*′-ethylcarbodiimide hydrochloride (EDC) *N*-hydroxysuccinimide (NHS), 2-(*N*-morpholino)ethanesulfonic acid (MES) buffer (0.1 M pH6.0)/dimethylsulfoxide (DMSO) 1:1; (ii) piperidine/ *N*,*N*-dimethylformamide (DMF)/DMSO (2:3:5); (iii) succinic anhydride, sodium bicarbonate buffer (0.1 M pH 8.3)/DMSO 1:1; (iv) EDC, sulfoNHS, MES buffer (0.1 M pH 6.0); (v) when an excess of carboxy-NPs are used an mMPIO with excess surface carboxylic acid functions is formed; when an excess of peptido-NPs is employed the mMPIO surface contains excess amine functions (not shown).

Biodegradable multimeric MPIO (mMPIO) were covalently assembled from multiple colloidal iron oxide NPs of diameter 65 nm bearing an amine-functionalized dextran coat (Fig. 2). These common precursor particles (Supplementary Figs 2 and 3 and Supplementary Tables 1 and 2) were then elaborated into two complementary NP subtypes that could be readily coupled together. In the first subtype, carboxylate groups were created from amino groups using succinic anhydride to form carboxy-NPs^26^ (Supplementary Fig. 3). In the second, peptide sequences were added to create peptido-NPs (Supplementary Fig. 3 and Supplementary Table 3). These peptide sequences were carefully designed to provide not only a suitable linker between monomer particles but also a linker that would be sensitive to specific intracellular degradative enzymes. Such proteases have previously been shown to display strong potential in, for example, targeted toxin release systems^27^,^28^ and are noted in differential regulation in some disease states^29^. The cathepsin proteins are the primary degradative enzymes in macrophages, which are the main site of sequestration of iron oxide particles in the liver and spleen on clearance from the circulation^30^,^31^,^32^. The proteolytic activity of lysosomal cathepsin B (EC 3.4.22.1) and L (EC 3.4.22.15) was tested against possible consensus peptide sequences^33^,^34^,^35^. The most efficiently cleaved (Supplementary Figs 4–8) yet plasma stable (Supplementary Figs 9 and 10) peptide was chosen and incorporated. Stability of this peptide under serum conditions was also tested (Supplementary Figs 11–13); assays revealed degradation following clotting of blood but stability upon treatment with EDTA (Supplementary Fig. 13), suggesting susceptibility to metal-dependent proteases induced in the clotting cascade (e.g., Factor IXa and XIa). To stringently test the specificity of these chosen sequences we created four homologues of the cathepsin L-specific peptide Fmoc-Ahx-Ahx-FVRGAGE (Supplementary Fig. 4). In these homologues, key residues were varied or scrambled, and D-amino acids were used (including a ‘mirror-image' peptide). When examined in detail, through the kinetics of cleavage combined with MS analysis (see Supplementary Figs 14–17 and Supplementary Table 5), these revealed much lower cleavage activity towards the scrambled and particularly the D-amino acid peptide, as expected.

**Figure 3:**
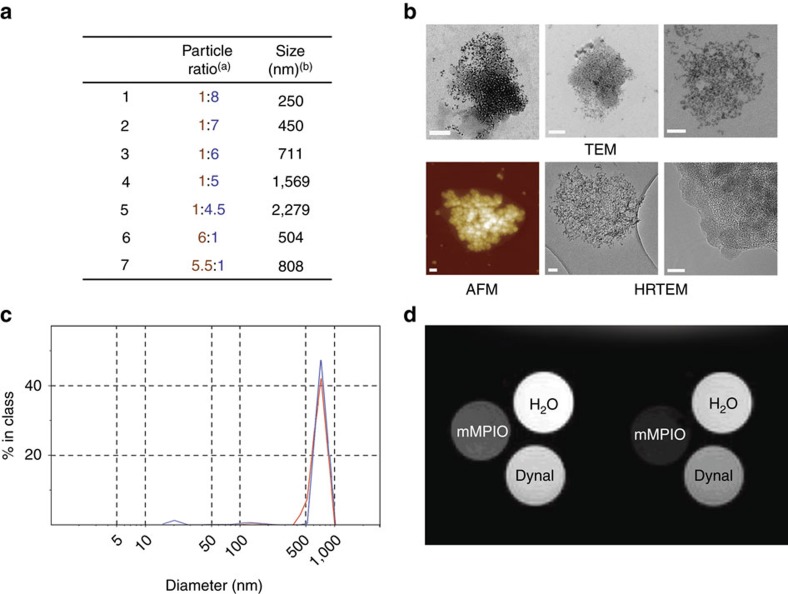
Physicochemical characterization of mMPIO. (**a**) Size and yield of mMPIO as a function of carboxy-NP/peptido-NP ratio. ^[a]^Ratio between carboxy-NPs and peptido-NPs (w/w). ^[b]^Determined by dynamic light scattering (DLS). (**b**) Microscopic characterization of mMPIO by TEM (top three images showing different representative examples; scale bars left-to-right 100, 100 and 50 nm), atomic force microscopy (bottom left, scale bar 50 nm) and HRTEM (bottom middle and right; bottom right is the inset magnification from bottom middle; scale bars 50 and 5 nm). (**c**) DLS size distribution graph of ∼700 nm mMPIO just after synthesis (red line) and after storage for 6 months (blue line). (**d**) T_2_-weighted images of the phantoms (iron concentration=0.224 mM) at echo times 9.7 ms (left) and 16.3 ms (right).

Using the carboxy-NP and peptido-NP monomers, larger mMPIO were synthesized. This synthesis was performed using robust amide-forming chemistry in MES buffer pH 6.0 via *N*-hydroxysulfosuccinimide (sulfoNHS) ester^36^. The resulting panel of mMPIO was characterized and optimized for key parameters, including size, yield and reactivity (Fig. 3a and Supplementary Table 4). Combined electron microscopy, transmission electron microscopy (TEM), atomic force microscopy (AFM), zeta potential and dynamic light scattering (DLS) measurements confirmed construction and the anticipated morphology and characteristics of our designed larger, multimeric particles (Fig. 3b and Supplementary Fig. 18 and Supplementary Tables 4 and 6). Composition and dextran content were further confirmed by chemical and elemental analyses. Importantly, by varying the reaction ratio of the two monomer types it was possible to change both the particle size and the nature of the surface reactive functional groups of the resulting mMPIO (Fig. 3a and Supplementary Table 4), which in turn would ensure that a broad range of targeting ligands could be conjugated. This approach, therefore, allowed us to readily create mMPIO bearing either an excess of amine (mMPIO-NH_2_) or those bearing an excess of carboxylic acid (mMPIO-COOH).

### mMPIOs show low sedimentation and high MRI relaxivities

We have previously shown that microparticles of 0.5–1 μm diameter combine favourable characteristics of high contrast effect and rapid blood phase clearance rate that are well suited to *in vivo* targeting studies^16^, by delivering efficient particle binding to the site of interest and low background signal at the time of MRI. At the same time, the MPIO are still much smaller than erythrocytes and not prone to microvascular plugging. mMPIO within this size range were efficiently and reliably constructed through modulation of the starting ratios of the two monomers (Fig. 3a). These mMPIO exhibited physicochemical advantages over equivalent monomeric iron-dextran MPIO (∼0.7 μm diameter; see Supplementary Methods for synthesis). In particular, the mMPIO showed very little tendency to precipitate, with sedimentation rates markedly lower than correspondingly sized monomeric particles and commercially available polystyrene-coated particles (<1, 51 and 86% of sedimentation at 24 h, respectively; Supplementary Figs 19 and 20); such effects potentially increase the translational potential of the mMPIOs and may be due to crosslinking or surface charge changes (although they are consistent with many different surface potential levels; Supplementary Fig. 3). Moreover, the mMPIO displayed storage stability at 4 °C for more than 6 months (Fig. 3c). They also exhibited significantly higher T_2_ relaxivities (194.5±4.5 mM^−1^ s^−1^ at 4.7 T and 196.5±4.9 mM^−1^ s^−1^ at 7 T; Fig. 3d and Supplementary Fig. 21 and Supplementary Table 7) than commercially available polystyrene-coated particles (58.5±2.2 and 59.5±1.2 mM^−1^ s^−1^ at 4.7 and 7 T, respectively; Supplementary Table 7) that have previously^16^ demonstrated good contrast *in vivo*, potentially due to previously noted magnetic relaxation switch phenomena^9^.

### mMPIO are biodegraded *in vitro* and *in cellulo*

**Figure 4:**
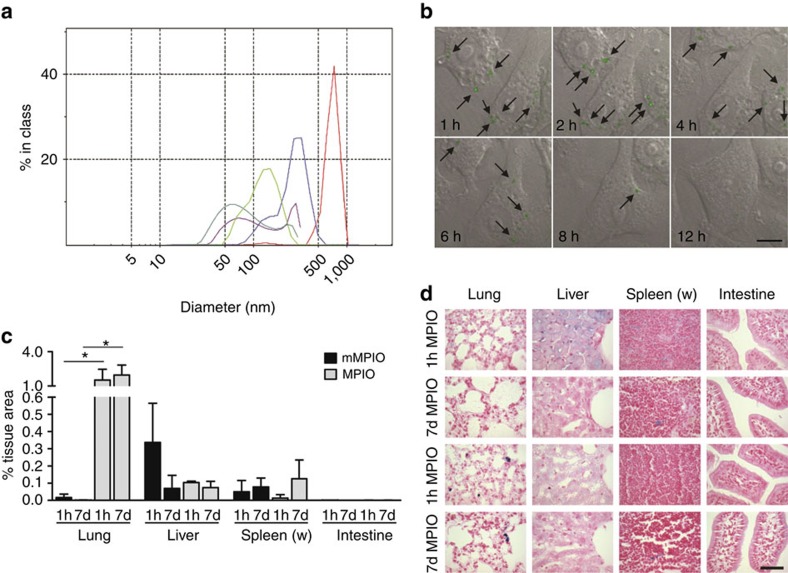
*In vitro* and *in vivo* degradation studies. (**a**) DLS analysis of particle size distribution of mMPIO after addition of cathepsin L. Particle size was analysed at different time points 0 h (red line), 1 h (blue line), 4 h (light green), 8 h (purple) and 24 h (dark green). Experiments were performed in triplicate. (**b**) Time-course montage of particle degradation by the murine macrophage cell line RAW264.7 obtained by live cell confocal imaging. Visible particles are indicated by black arrows. Experiments were performed in triplicate. Scale bar 10 μm. (**c**) Graph to show distribution of ca. 700 nm mMPIO and MPIO in different organs 1 h and 7 days after intravenous injection (*n*=3 per group). Data are mean±s.d. for three fields per organ. *P<0.05 based on one-way ANOVA on a tissue-wise basis followed by Newman–Keuls *post-hoc* tests. (**d**) Images of immunohistochemical sections taken from different organs 1 h and 7 days after intravenous injection of either mMPIO or MPIO. Sections have been stained with Prussian Blue to identify iron and counterstained with Nuclear Fast Red. Magnification is × 400, scale bar=50 μm.

Next, the biodegradability of the mMPIO was evaluated *in vitro*. Consistent with their molecular design, incubation of mMPIO with both cathepsins B and L efficiently degraded the peptide linker yielding the monomeric NPs from which they were built (Fig. 4a). Having demonstrated the biodegradable nature of the linker with respect to the appropriate enzymes, cellular uptake and intracellular degradation of the mMPIO were evaluated. Both amino-terminated mMPIO (mMPIO-NH_2_) and carboxylic acid-terminated mMPIO (mMPIO-COOH) (entries 3 and 7 from Fig. 3a, respectively) were labelled with the fluorophore AlexaFluor 488 cadaverine. These fluorescently labelled mMPIO were incubated with cultured macrophages. Confocal time-course experiments showed a consistent and clear reduction in the number of intracellular mMPIO over 12 h (Fig. 4b, Supplementary Figs 22 and 23 and Supplementary Movie 1). Further experiments (Supplementary Figs 24 and 25) showed that punctuate fluorescence in the intracellular compartment faded over time. After 72 h, the fluorescence was homogeneously distributed and no large particles were detectable, suggesting complete degradation. By comparison, the equivalent large (733 nm) amino-terminated monomeric MPIO remained intact after 48 h, with only slight degradation after 72 h (Supplementary Fig. 26), suggesting a considerably slower rate of degradation compared with the mMPIO. Cellular uptake of the mMPIO was confirmed and subcellular distribution of the degradation products was also analyzed in macrophage cell line (RAW 264.7) using TEM (see Supplementary Fig. 27) over 72 h. Upon internalization into the distinctive cell morphology of the cells, mMPIO were clearly surrounded by visible membrane structures, suggesting anticipated formation of endolysosomal compartments within which the structure of the mMPIO degraded. This confirmed the uptake into phagosomes of high-intensity material containing iron and then its loss over time. In contrast, commercially available monomeric MPIO beads remained intact (with high iron content) throughout. We also constructed mMPIOs linked by peptides containing D-amino acids in the same sequence used for the degradable mMPIOs; consistent with the resistance shown by D-amino acid-containing peptides to cathepsins, these ‘D'-mMPIO were not degraded (Supplementary Figs 28–32). These experiments further supported both the efficacy and the molecular mechanistic basis of the designed degradable ‘L'-mMPIO system.

### mMPIOs are not non-specifially retained and clear rapidly

Having confirmed *in vitro* degradation of the mMPIO, the *in vivo* biodistribution and clearance of the mMPIO were evaluated histologically (Fig. 4c,d and Supplementary Figs 33 and 34). Both mMPIO-NH_2_ and the equivalent size, amino-terminated, monomeric MPIO were injected intravenously into naïve mice. Primary uptake of both the mMPIO and monomeric MPIO was evident in the liver 1 h after injection, and in both cases the particles were almost entirely cleared from this site by 7 days. Low-level mMPIO retention was also observed in the intestine and lung at 1 h, although the level of particle staining was low compared with that observed in the liver (0.001 and 0.044, respectively, versus 0.208% area stained) and, again, was negligible by 7 days. Similar low levels of monomeric MPIO retention were found in the intestine. In striking contrast, substantial monomeric MPIO retention was evident in the lungs, which was lower but still evident 14 days post-injection (*ca*. 1% tissue area). The lung is known to play an important role in removing blood-borne foreign bodies via adherent phagocytes and endothelial cells, and particles cleared in this way are passed through the endothelium to accumulate in macrophages in the alveolar interstitium^37^. Thus, it might be expected that MPIO would also be cleared from the circulation by this pathway. Unlike the MPIO, however, the mMPIO were not cleared to any appreciable extent via the lungs (cf. 1 h data; Fig. 4c,d and Supplementary Fig. 34), reflecting their different physicochemical properties. Very low and constant levels of iron staining were evident in the white pulp of the spleen in both mMPIO- and MPIO-injected animals (0.1–0.2% tissue area), while the very high intrinsic iron levels in the red pulp of the spleen precluded quantitation of the small increases arising from mMPIO/MPIO accumulation. No appreciable retention of the mMPIO or monomeric MPIO was found in heart, brain or kidney (<0.0005% of tissue area; Supplementary Fig. 33). Importantly, for our goal of creating a flexible particle system, chemical surface functionality did not alter *in vivo* distribution; no appreciable differences were seen in animals injected with mMPIO-COOH compared with those injected with mMPIO-NH_2_.

To assess whether labelling of the mMPIO with targeting antibodies altered the clearance profile and to obtain formal toxicological data, preclincial studies were conducted (Sequani; see Supplementary Methods) in which mice were injected with mMPIO conjugated to a humanized anti-human-vascular cell adhesion molecule (VCAM)-1 antibody (αhuVCAM) with cross-reactivity to mouse (αhuVCAM-mMPIO). All values for clinical observations (body weight, organ weights, macroscopic histology) were within normal ranges, and no blood chemistry (Supplementary Tables 9 and 10), haematological (Supplementary Tables 11 and 12) or histological findings (Supplementary Fig. 35) were of toxicological significance. In accord with our findings above, a very low level of diffuse iron staining was found in the liver 2 days after administration of αhuVCAM-mMPIO in 5/6 mice, which was no longer evident by 14 days post-αhuVCAM-mMPIO injection (Supplementary Fig. 35). In-house quantitative analyses confirmed the absence of particulate iron deposits (i.e., undegraded mMPIO) at both 2 and 14 days post-αhuVCAM-mMPIO injection, indicating degradation within the first 48 h period. No αhuVCAM-mMPIO retention was evident in any other tissue, or in control animals, at either time point. Thus, the biodistribution profile of the mMPIO remained the same when conjugated to a targeting antibody. Since VCAM-1 is not highly expressed on vascular endothelium under normal conditions, accumulation in tissue beds expressing VCAM-1 other than the brain was not examined, as this would have required different disease models. No evidence of infarction or inflammation was found in any of the organs studied in any of the above studies, up to 14 days post-mMPIO injection.

### Antibody-targeted mMPIOs allow molecular imaging

Having established biodistribution profiles, the utility of this platform to create targeted mMPIO as tools for molecular imaging was tested. The diversity of functional groups in the mMPIO, which is a consequence of their multimeric assembly, also allowed ready orthogonal labelling such that different moieties could be incorporated via different functional groups (Supplementary Fig. 36). In this way, mMPIO were created that contained both multiple fluorescent labels (AF488) in the inner core of the particle and high levels of surface, targeting moieties. Particles were synthesized bearing either an anti-VCAM-1 antibody (αVCAM) or a corresponding IgG control antibody (αVCAM-AF488-mMPIO and IgG-AF488-mMPIO, respectively). Both targeted particles exhibited similarly high surface antibody density (Supplementary Fig. 37). As expected, αVCAM-AF488-mMPIO, but not IgG-AF488-mMPIO, showed high binding capacity towards activated endothelial cells *in vitro* (Supplementary Fig. 38).

**Figure 5:**
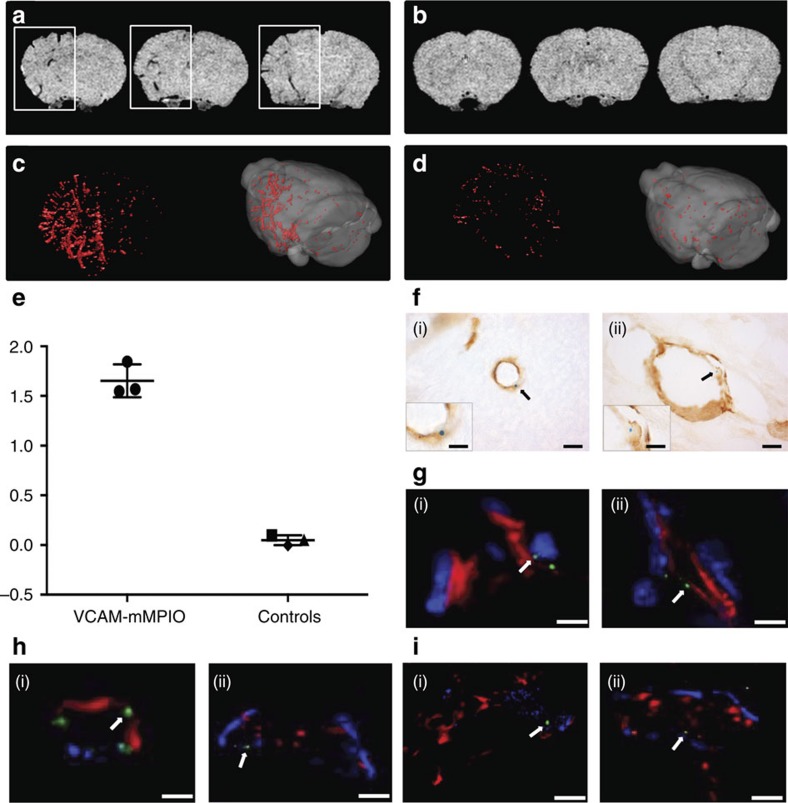
*In vivo* VCAM-1-targeting experiments. (**a**,**b**) Selected T_2_*-weighted images from 3D data sets obtained from mice injected intrastriatally with IL-1β 3 h before intravenous injection of either (**a**) αVCAM-AF488-mMPIO or (**b**) control non-targeted IgG-AF488-mMPIO. Focal hypointense areas (black) in the left hemisphere reflect the specific αVCAM-AF488-mMPIO retention on acutely activated vascular endothelium (**a**), with negligible contrast effects evident in either the contralateral control hemisphere or in the animal injected with IgG-AF488-mMPIO (**b**). Box indicates the injected hemisphere. (**c**,**d**) 3D reconstructions of the mMPIO-induced hypointensities (coloured in red) from mice injected with either αVCAM-AF488-mMPIO (**c**) or IgG-AF488-mMPIO (**d**) shown both as a stacked plot (left) and within the mouse brain frame of reference (right). Negligible contrast effects are evident in the animal injected with IgG-AF488-mMPIO. (**e**) Graph to show volumes of hypointensities in αVCAM-AF488-mMPIO-injected mice (black circles; *n*=3) and controls (black square, IL-1β+IgG-AF488-mMPIO; black triangle, saline+IgG-AF488-mMPIO; black diamond, naive+αVCAM-AF488-mMPIO). Data are shown as specific contrast (left–right difference) for each animal; note negligible specific contrast is apparent in the control mice. Each data point represents an individual mouse; since this was a proof-of-principle experiment to demonstrate *in vivo* contrast effects, statistical analysis was not performed. (**f**–**i**) Immunohistochemical and immunofluoresence images of brain sections taken from IL-1β-injected mice. **f** (i, ii) Immunohistochemical sections showing VCAM-1 expression (brown) co-localized with αVCAM-AF488-mMPIO (Prussian Blue staining). Epifluorescence (**g** (i)) and confocal (**g** (ii)) images further demonstrate adherence of αVCAM-AF488-mMPIO (green) to VCAM-1-positive (red) vessels. Nuclei are stained with DAPI (blue). Additional alkaline phosphatase staining enabled co-localization of αVCAM-AF488-mMPIO (green) with VCAM-1 (blue) and vessels (laminin—red) to be confirmed using both epifluorescence (**h** (i)) and confocal (**h** (ii) and **i** (i, ii)) microscopy. Arrows indicate αVCAM-AF488-mMPIO. Scale bars=10 μm (**f** (i,ii); **h** (ii); **i** (i,ii)) and 5 μm (**g** (i,ii); **h** (i)).

Finally, the potential of the αVCAM-AF488-mMPIO for *in vivo* molecular MRI was evaluated in a mouse model of cerebral inflammation. Mice were injected intracerebrally with interleukin-1β (IL-1β) in the left striatum to induce endothelial activation and VCAM-1 expression^16^. In animals subsequently injected intravenously with αVCAM-AF488-mMPIO, a marked contrast effect was evident in the T_2_*-weighted images, manifest as focal hypointensities in the IL-1β-injected hemisphere (Fig. 5a,c, Supplementary Fig. 39a and Supplementary Movie 2). Notably, the contrast effect was unilateral with no non-specific mMPIO-induced hypointensities in the non-injected hemisphere. Negligible contrast effects arising from mMPIO retention were present in any of the controls: (i) naïve mouse injected intravenously with αVCAM-AF488-mMPIO; (ii) mouse injected intracerebrally with saline and intravenously with αVCAM-AF488-mMPIO; and (iii) mouse injected intracerebrally with IL-1β and intravenously with the non-targeted IgG-AF488-mMPIO (Fig. 5b,d, Supplementary Fig. 39b and Supplementary Movie 3). Quantitative analyses of the volumes of hypointensity induced by αVCAM-AF488-mMPIO binding yielded substantially greater volumes in the IL-1β-injected animals than in any of the control animals (Fig. 5e). Subsequent T_1_-weighted images acquired after intravenous administration of the passive contrast agent gadolinium-DTPA revealed no areas of contrast enhancement in any animal, verifying that the blood–brain barrier was intact. Taken together, these data indicate specific binding of αVCAM-AF488-mMPIO to acutely activated endothelium in the absence of blood–brain barrier breakdown.

Following the *in vivo* MRI experiments, co-localization of VCAM-1 expression and αVCAM-AF488-mMPIO binding was verified both immunohistochemically and by immunofluorescence (Fig. 5f–i). Immunohistochemical analysis demonstrated upregulation of VCAM-1 in the IL-1β-injected, but not the contralateral, hemisphere. Subsequent Prussian Blue detection of iron revealed the presence of bound αVCAM-AF488-mMPIO in VCAM-1-positive vessels (Fig. 5f). Both epifluorescence and confocal microscopy of the brain sections further confirmed successful co-localization of the αVCAM-AF488-mMPIO with both VCAM-1 and laminin, indicating association of the targeted mMPIO with VCAM-1-positive vessels (Fig. 5g–i).

## Discussion

We have demonstrated here some potential advantages conferred by the use of covalent linkages for the synthesis of mMPIO that include control of size and enhanced stability. These are both prerequisites for clinical use of the agent that are not provided by, for example, current methodologies based on non-covalent linkages^8^,^9^.

We have demonstrated here these mMPIOs as intravascular agents. In the system we propose here, molecular targeting is determined by a surface-displayed binding agent on the particle and an appropriate ‘biomarker' binding partner on the cell surface. We should add the clear caveat that we have only tested a single targeting antibody type (anti-VCAM); other important target sites also exist (e.g., interstitial space or poorly vascularized tumour cells) for imaging on which they might also be tested in the future. However, at such sites targeted agents may lose their molecular selectivity since they can also accumulate passively.

The choice of a cathepsin-cleavable peptide as the linker ensured rapid degradation of the mMPIO once sequestered by macrophages, primarily within the liver following clearance from circulation^30^,^31^, while particles that associate with their target remain unaltered and functional. Rapid sedimentation, slow degradation and mechanical retention in organs^23^,^24^, such as the lung, make the corresponding monomeric MPIO particles unsuitable for use in man. In contrast, the mMPIO appear to possess properties that are useful for clinical application of molecular imaging, such as high relaxivity (see Supplementary Table 8), unappreciable sedimentation rate, rapid degradation, no overt toxicity and fast clearance from circulation. Owing to these mechanistic differences, unlike toxic particles where dose would be rapidly limiting, excesses of mMPIO can advantageously be used. In this way, percentage binding can be even tuned accordingly, since any excess that does not bind is non-toxic, cleared rapidly and degraded. This therefore provides both a vital mechanistic and potential translational advantage. Thus, while key additional translational hurdles will need to be considered in even greater detail for future development and regulatory approval (i.e., stability, reproducibility, dispersity), we believe that these proof-of-principle experiments with mMPIO might provide a promising platform for the clinical use of molecular MRI contrast agents.

## Methods

### General considerations

All animal experiments were authorized by the UK Home Office. Chemical abbreviations and details of the equipment employed are given in Supplementary Methods.

### Synthesis of dextran covered NPs

About 5 mmol of FeCl_3_·6H_2_O and 9, 10 or 12 g of dextran average mol. wt. 9,000–11,000 (SigmaAldrich, cat. Nr. D9260, lot Nr. 1331472 or 1382459) or 7.3 g of Dextran 10 Pharmaceutical Quality (Pharmacosmos, batch Nr. HX4271) were dissolved in 20 ml of water and deoxygenated thoroughly by repeated cycles of vacuum assisted by sonication and argon flushing. After the first deoxygenation cycle, 3 mmol of FeCl_2_·4H_2_O in 5 ml of water was added and the solution was deoxygenated by the above procedure (four times). While being stirred with an overhead stirrer at 600 r.p.m., NH_4_OH (4 ml, 25%) was added at a rate of 168 ml h^−1^. The reaction was heated to 80 °C and then stirred at this temperature for 1 h. The solution was cooled and placed in a SpectraPor membrane (MWCO 100,000) leaving some space for volume increase. The solution was dialysed against 5 l of water for 21 h with water changes at times 1, 2 and 4 h. Iron content was measured spectrophotometrically at 410 nm after acid dissolution (6 M HCl (aq)) and oxidation (3% hydrogen peroxide solution) for 1 h (ref. 38), and adjusted to a final concentration of 10 mg Fe per ml using a Vivaspin centrifugal unit (MWCO 30.000). An aliquot (10 μl) was diluted with 700 μl of phosphate-buffered saline (PBS) and particle size was determined by DLS. A sample (5 mg of iron) was freeze dried and elemental analysis was determined (Supplementary Methods and Supplementary Table 1).

### Synthesis of amino-terminated NPs

Twenty millilitres of dextran covered NPs (10 mg ml^−1^) were placed into a 250-ml round flask equipped with a 30 × 16 mm oval stirrer bar. While the solution was stirred at 500 r.p.m., 36.7 ml of NaOH 5 M was added at a rate of 168 ml h^−1^. After that, 13.3 ml of epichlorohydrin (20 ml in case of Phamacosmos HX4271 dextran, results ‘k–l' Supplementary Table 1) was added at a rate of 94 ml h^−1^. The mixture was stirred at 1,000 r.p.m. for 7 h and then 20 ml of NH_4_OH (25%) was added at a rate of 168 ml h^−1^. The mixture was stirred at 1,000 r.p.m. for 14 h and then was placed in a SpectraPor 2 or 100 kDa cutoff membrane leaving some space for volume increase. The solution was then dialysed against 5 l of water for 21 h with water changes at times 1, 2 and 4 h and the dialysate concentrated on a Vivaspin 15 unit (MCWO 30.000) to 15 mg Fe per ml. A sample (5 mg of iron) was freeze dried and elemental analysis was determined (Supplementary Methods and Supplementary Table 2).

### Synthesis of carboxylic acid terminated NPs

Succinic anhydride in dimethyl sulfoxide (DMSO) (4 ml, 15 mg ml^−1^) was added to a solution of amino-NPs (8 ml, 10 mg Fe per ml) in sodium bicarbonate buffer 100 mM pH 8.3. Note: The reaction is exothermic and a nitrogen-filled balloon with a syringe was placed on the tube. The mixture was shaken for 3 h, then 4 ml of succinic anhydride in DMSO (15 mg ml^−1^) was added and the mixture shaken for a further 3 h. The sample was dialyzed in a 10,000 Da dialysis membrane against 5 l of water for 21 h with water changes at times 1, 2 and 4 h and was concentrated in a Vivaspin 20 unit (MCWO 30.000) to 15 mg Fe per ml.

### Peptide synthesis

SPPS peptide synthesis was performed under standard Fmoc-conditions using HBTU/HOBt as an activator and DIPEA as a base catalyst. Deprotection and couplings were performed under microwave irradiation employing a single coupling protocol except for the first residue, which was introduced by double coupling. Peptide cleavage was performed using TFA/TIS/H_2_O 95:2.5:2.5 (10 ml per g resin). The solvent was partially evaporated and the peptide was precipitated by addition of a cold mixture of diethylether/hexane 1:1, centrifuged and washed again with the same mixture. The peptide was homogenized, dried under high vacuum for 3 h, re-dissolved in water/acetonitrile/DMSO/TFA 70:15:5:0.1 and purified by preparative RP chromatography. This product was dissolved in 2% DMSO in water and analysed by high-performance liquid chromatography (HPLC). For structure of peptides, see Supplementary Fig. 4. Peptides were characterized by HPLC, high-resolution mass spectrometry and tandem mass spectrometry (MS/MS) (Supplementary Figs 40–47 and Supplementary Tables 13–17). The proteolytic activity of the cathepsins on the peptides was analyzed by HPLC (Supplementary Methods).

### Synthesis of peptide covered NPs (peptido-NPs)

A solution of EDC in water (0.84 ml, 38 mg ml^−1^) was added to a solution of peptide 3 (200 mg) and NHS (24 mg) in DMSO (3.7 ml). This solution was incubated for 5 min at r.t. and then added to a solution of 20 mg of amino-NPs in 3.3 ml of MES buffer 0.1 M pH 6.0. The solution was shaken for 3 h at room temperature (r.t.), and then split into two 50 ml centrifuge tubes. Particles were precipitated by addition of 45 ml of acetonitrile, centrifuged for 20 min at 3250*g* and the supernatant was carefully discarded taking care not to disturb the precipitate. The precipitate was re-suspended in 5 ml of DMSO, an aliquot (100 μl) was taken and Fmoc analysis was performed (Supplementary Methods). The rest of the sample was precipitated again by addition of 45 ml of acetonitrile and centrifuged as before. Samples were re-suspended in 5 ml of DMSO and 5 ml of 40% piperidine in DMF was added. The samples were shaken for 30 min after which the particles were precipitated by addition of 45 ml of 1,4-dioxane. Samples were re-suspended in 5 ml of DMSO and the deprotection step was repeated. Precipitate was re-suspended in water, dialyzed in a 10.000 MWCO dialysis membrane against 5 l of water for 21 h with water changes at times 1, 2 and 4 h and was concentrated in a Vivaspin 6 unit (MCWO 30.000) to 15 mg Fe per ml (Supplementary Table 3). An essentially similar method was used for the synthesis of D-peptido-NPs containing D-amino acids in the peptide.

### Synthesis of amino-terminated mMPIOs (mMPIO-NH_2_)

A solution of carboxy-NPs (25.5 μl, 11.2 mg Fe per ml, 0.285 mg Fe) in MES buffer 0.1 M pH 6.0 was activated by sequential addition of sulfoNHS (2 μl, 1.5 eq. respect to the N content of its amino-NPs precursor; see Supplementary Table 2) and of EDC (1 μl, 1.2 eq. respect to the N content of its amino-NPs precursor; see Supplementary Table 2) in MES buffer 0.1 M pH 6.0. The resulting solution was shaken and incubated at r.t. for 5 min and added to a solution of peptido-NPs (171 μl, 10 mg ml^−1^, 1.71 mg Fe) in MES buffer 0.1 M pH 6.0. The reaction was shaken at 55 °C for 24 h at 1,400 r.p.m.. Once the reaction had finished the sample was diluted and purified by dialysis and either by magnetic pelleting (Supplementary Methods) or by sucrose gradient (Supplementary Methods). An aliquot (2 μl) was redispersed in 700 μl of PBS and size was measured by DLS (Supplementary Table 4). An essentially similar method was used for the synthesis of the D-amino-acid-mMPIOs from D-peptido-NPs.

### Synthesis of carboxy-terminated mMPIOs (mMPIO-COOH)

A solution of carboxy-NPs (122 μl, 15.3 mg Fe per ml, 1.71 mg Fe) in MES buffer 0.1 M pH 6.0 was activated by sequential addition of sulfoNHS (15 μl, 1.5 eq. respect to the N content of its amino-NPs precursor; see Supplementary Table 2) and of EDC (10 μl, 1.2 eq. respect to the N content of its amino-NPs precursor; Supplementary Table 2) in MES buffer 0.1 M pH 6.0. The resulting solution was shaken and incubated at r.t. for 5 min and added to a solution of peptido-NPs (22.8 μl, 12.5 mg per ml, 0.285 mg Fe) in MES buffer 0.1 M pH 6.0. The reaction was shaken at 55 °C for 24 h at 1,400 r.p.m.. Once the reaction had finished the sample was diluted and purified by dialysis and either by magnetic pelleting (Supplementary Methods) or by sucrose gradient (Supplementary Methods). An aliquot (2 μl) was redispersed in 700 μl of PBS and particle size measured (Supplementary Table 4).

### Sedimentation of particles

A solution of mMPIO or 733 nm MPIO (for synthesis see Supplementary Methods) (0.11 mg Fe per ml) was placed, after vortexing and sonication, in a quartz cuvette equipped with PTFE stopper. Absorption was measured at *λ*=500 nm for 24 h at 5 min time intervals.

### Relaxivity of compounds

Phantoms were prepared by addition of 0.75 ml of an agarose solution (12%) to 0.75 ml of serial dilutions of the contrast agent in a Nalgene Cryoware vial. Samples were centrifuged at 1,000*g* for 5 min to eliminate air bubbles. MRI experiments were performed on Magnex 4.7 or 7.0 T superconductive magnet driven by Varian DirectDrive spectrometer (Magnex Scientific and Varian Inc.; subsidiaries of Agilent Technologies, Santa Clara, CA, USA). A spin-echo sequence was used to acquire T_2_ and T_1_ maps. Single slice images were acquired with a matrix size of 128 × 128 pixels in all cases, corresponding to voxel dimensions of 0.4 × 0.4 × 5.0 mm. T_2_ maps were generated from a series of spin echo images (repetition time (TR)=3.0 s) in which the echo time (TE) was logarithmically distributed in 10 steps from 9.7 to 100 ms. The total experimental time was ca. 1 h. T_1_ maps were generated from a series of inversion recovery spin echo images (TR=10.0 s; TE=9.7 ms) in which the inversion recovery time was logarithmically distributed in 10 steps from 10 ms to 6.0 s. The total experimental time was ca. 3.5 h. The relaxation maps were calculated using a standard mono-exponential fit employing a least-squares procedure.

### *In vitro* degradation of mMPIO by cathepsin L

Cathepsin L (0.88 μg) in 98 μl of citrate buffer 0.1 M pH 5.0 containing 1 mM EDTA was activated by addition of DTT (2 μl, 100 mM in water). After 15 min at r.t., this pre-activated solution was added to 0.4 mg of mMPIO in 400 μl citrate buffer pH 5.0. The solution was incubated at 37 °C and at selected time points 100 μl of solution was taken and 1 μl of the potent cysteine protease inhibitor E-64 (Sigma-Aldrich, UK) solution 1 mM in DMSO was added. After 1 h at 4 °C the sample was diluted to 700 μl and particle size measured.

### *In vitro* macrophage uptake and degradation experiments

The synthesis of fluorescent-labelled mMPIO (with both L- and D-amino acid peptide linkers) and monomeric MPIO was performed as described in Supplementary Methods. RAW264.7 murine macrophage cells (ATCC) were grown in DMEM supplemented with 10% FBS until ca. 50% confluent. Cells were incubated with 1 μg of multimeric particles (per 35 mm culture dish) for 30 min at 37 °C. Culture medium was replaced with fresh, pre-warmed complete Dulbecco's Modified Eagle's medium, and live cell experiments performed at 37 °C using a Zeiss LSM510 laser scanning confocal equipped with a microscope incubator (CO_2_=5%). Images were collected using a 63X 1.4NA objective, with the 488-nm laser line of an argon laser used for fluorescence and transmitted light imaging. Twelve hours time courses were run; imaging interval, 2 min. Further experiments were performed in freshly isolated murine macrophages (Supplementary Methods) and the subcellular degradation process was monitored by TEM (Supplementary Methods).

### *In vivo* mMPIO uptake and clearance experiments

Adult male NMRI mice (30–40 g) were anaesthetized and injected intravenously with 4 mg Fe per kg body weight of (i) mMPIO-NH_2_ or (ii) equivalent sized amine-terminated monomeric MPIO. Animals were transcardially perfused with saline followed by 4% paraformaldehyde at either 1 h or 7 days (*n*=3 per group) after microparticle injection. Two additional animals were injected with the same dose (4 mg Fe per kg) of mMPIO-COOH and perfused 7 days post-injection. A further group of animals injected with the monomeric MPIO were perfused 14 days post-injection (*n*=3). All *in vivo* experiments were approved by the UK Home Office.

Tissue samples from brain, heart, lung, kidney, liver, spleen and intestine were post-fixed for 7 days and paraffin wax embedded. Ten micrometre sections were dried overnight, de-waxed and stained for iron using Perls' Prussian Blue stain with a Nuclear Fast Red counterstain. For each tissue, three fields of view per animal at × 400 magnification were analysed using a semiautomated thresholding method (ImageJ) to determine the percentage area of iron staining. Thresholding criteria, based on hue, saturation and luminosity values specific to the microparticles were constant for each tissue. Differences between groups were assessed using ANOVA followed by *post-hoc* pairwise *t*-tests with a Welch's correction for unequal variances where appropriate; group sizes were based on previous clearance experiments with commercial MPIO. Mice were assigned randomly to each group, and all analysis was performed blind to the sample groups.

Subsequently, a single dose extended acute toxicology study was commissioned from the Commercial Research Organization Sequani Ltd (full details in Supplementary Methods). The following assessments were made: body weight, organ weights, macroscopic pathology, haematology and blood chemistry. In addition, microscopic analysis, including assessment of iron deposition, was conducted on brain (at three levels), heart, kidney, liver, lung and spleen tissue. Subsequent in-house quantitative analysis of Perls' staining was performed as described above. Group sizes were determined by Sequani Ltd based on previous toxicology work, mice were assigned randomly to each group and analysis was performed blind to sample group.

### Synthesis of fluorescently labelled, targeted mMPIOs

A solution of AlexaFluor 488 SDP ester (2 mg per ml, 250 μl) in DMSO was added to a solution of mMPIO-COOH (5 ml, 1 mg Fe per ml) in sodium bicarbonate buffer 0.1 M pH 8.3. The reaction was shaken for 24 h. Particles were collected using a Dynal magnet (Invitrogen, UK) and washed. Particles were resuspended in 900 μl of MES buffer 0.1 M pH 6.0. SulfoNHS (2 μl, 1 M) in water and EDC (3.5 μl, 0.1 M) in water were sequentially added to 180 μl of the AF488-mMPIO solution. The resultant solution was stirred for 15 min at room temperature and then 400 μl of rat anti-mouse VCAM-1 (clone M/K2) or rat IgG_2a_ isotype control antibody (clone KLH/G2a-1-1) (0.5 mg ml^−1^) followed by 400 μl of sodium bicarbonate buffer 0.1 M pH 8.3 was added. The sample was shaken for 24 h. Particles were collected using a magnet (Invitrogen) washed and resuspended in 200 μl of PBS buffer. The antibody loading was determined on multimeric particles from fluorescence intensity using Qifikit calibration beads (Dako, UK) according to the manufacturer's protocol, but substituting the provided fluorescently conjugated antibody with AlexaFluor594-conjugated rabbit anti-rat IgG (H+L) (Invitrogen; Cat. Nr. A-21211). mMPIO containing 5 μg iron were diluted in 200 μl of 1 × PBS/0.1% Tween-20. AlexaFluor594-conjugated goat anti-mouse IgG (H+L) (Invitrogen; Cat. Nr. A-11005) (8 μg) was added and incubated at 4 °C for 1 h. The particles were pelleted on a Dynal magnetic separator (Invitrogen) and washed three times with 1 × PBS/0.1% Tween 20. Flow cytometry experiments were performed on a BD LSRII flow cytometer and the data analysed using Flow Jo (Tree Star Inc., OR, USA).

### *In vitro* antibody-mMPIO binding experiments

Murine endothelial (sEnd.1, PMID: 2736622) cells, cultured in 35 mm dishes cultured in 35 mm dishes (Corning, USA), were stimulated with recombinant mouse tumor necrosis factor-α for 8 h, fixed with 4% formaldehyde for 10 min at room temperature, washed with PBS and stored at 4 °C. αVCAM-AF488-mMPIO (1.625 μg Fe per ml), IgG-AF488-mMPIO (1.625 μg Fe per ml) or PBS was added to cells and these were placed on a bench-top rocker for 5 min at r.t. prior to thorough washing with PBS. Particle binding events were visualized using a × 40, 0.6 NA objective fitted to an Olympus IX-71 microscope.

### *In vivo* anti-VCAM-1-targeted experiments

Adult male NMRI mice (30–40 g or 10–12 weeks, *n*=3) were anaesthetized with 2.0–2.5% isoflurane in 70% N_2_O:30% O_2_ and stereotaxically microinjected in the left striatum (co-ordinates from Bregma: anterior 0.5 mm, lateral 2 mm, depth 2.5 mm) with 20 ng of recombinant mouse IL-1β in 1 μl low endotoxin saline (*n*=3). After 3.1±0.1 h, mice were re-anaesthetized and injected via a tail vein with αVCAM-AF488-mMPIO (4 mg Fe per kg). Three control mice were studied: (i) naïve mouse injected intravenously with αVCAM-AF488-mMPIO as above; (ii) mouse injected intracerebrally with vehicle (1 μl saline) and intravenously with αVCAM-AF488-mMPIO after 3.2 h; and (iii) mouse injected intracerebrally with 20 ng IL-1β in 1 μl saline and intravenously with the non-targeted IgG-AF488-mMPIO after 3.0 h. Following mMPIO injection, animals were positioned in a quadrature birdcage coil. During MRI, anaesthesia was maintained with 1.5–1.8% isoflurane, and electrocardiogram was monitored and body temperature maintained at ∼37 °C. All *in vivo* experiments were approved by the UK Home Office.

### Magnetic resonance imaging

A T_2_*-weighted three-dimensional gradient echo data set was acquired: flip angle=27°; TR=65 ms; TE=7.5 ms; field of view (FOV)=11.2 mm × 22.5 mm × 22.5 mm; matrix size, 96 × 192 × 256; number of averages=2; total acquisition time ∼40 min. Mid-point of acquisition was 1.6±0.4 h after microparticle injection. Data were zero-filled to 128 × 256 × 256; final isotropic resolution ∼88 μm. Brains were masked, thresholded and MPIO binding quantified. Spin-echo T_1_-weighted images (TR=500 ms; TE=20 ms; FOV 25 mm × 25 mm; matrix size, 128 × 128) were acquired pre- and 5 min post-Gd-DTPA injection (Omniscan; GE Healthcare, UK; 30 μl, i.v.). Each T_2_*-weighted data set was converted into tiff images, manually masked to exclude extracerebral structures and converted to 8-bit greyscale in Adobe Photoshop (Adobe Systems Incorporated, UK). The images were thresholded at a consistent level in the grey channel, such that any pixels of signal intensity >3 s.d. below the mean intensity of normal brain were set to zero (black) and all others were set to 1 (white), see Supplementary Fig. 39. The absolute level of thresholding varied between data sets according to variations in signal-to-noise. Signals arising from ventricles or sinuses were excluded by comparison to a naïve animal imaged with no contrast agent, in which these structures appear hypointense naturally. The masked and thresholded images were subsequently imported into ImagePro (Media Cybernetic, UK) and stacked into a single sequence. mMPIO binding, defined as all pixels with signal levels of zero, was quantified in 160 consecutive brain slices for each animal. Analysis was performed blind to the origin of the dataset. Segmented images were reconstructed using the three-dimensional Constructor plug-in to visualize the spatial distribution of binding, with low-signal areas assigned to the red channel and the anatomical image to the green channel. Voxel volumes were summed and expressed as raw volumes in microlitres with no surface rendering or smoothing effects. Since these were proof-of-principle experiments, to demonstrate the *in vivo* contrast effects of the mMPIO, only a sufficient number of animals were studied to demonstrate that these effects were reproducible and statistical analysis was not performed.

### Immunohistochemistry and immunofluorescence

Following MRI, animals were transcardially perfused and the brains post-fixed, cryoprotected, embedded and frozen in isopentane. Expression of VCAM-1 and co-localization with mMPIO was verified both by immunofluorescence microscopy and immunohistochemically (rat anti-mouse VCAM-1 (clone M/K2)) using avidin–biotin complex amplification^22^. To verify co-localization of the mMPIO with VCAM-1 expression, Prussian-blue staining to detect iron oxide was performed. Following completion of VCAM-1 immunohistochemistry, some slides were incubated with 20% hydrochloric acid and 10% potassium hexacyanoferrate(II) trihydrate for 20 min at 37 °C. After washing with PBS, the slices were then counterstained with cresyl violet.

### Microscopy

Images were acquired using either an inverted epifluorescence microscope (DM IRB; Leica Microsystems, Wetzlar, Germany) or an inverted confocal microscope (LSM-710; Carl Zeiss Microimaging, Jena, Germany) and analysed using Image J and Zen (Carl Zeiss) software.

The LSM-710 confocal microscope was equipped with PC-Apochromat × 63 1.2NA oil immersion objective lens. Detection ranges were set to eliminate crosstalk between fluorophores: 409–485 nm for DAPI, 494–553 nm for AlexaFluor 488 and 564–712 nm for Cy3. The set of dichroics MBS 488/561 and MBS-405 were used. The 32 PMT array of the confocal was used to record lambda images that were subsequently unmixed with the Zen software using individually recorded spectra, thus removing inherent autofluorescent signal from the tissue.

### Data availability

The particle sizing, MRI, microscope images and HPLC data that support the findings of this study are available in Oxford University ORA data system with the identifier https://doi.org/10.5287/bodleian:qaY9QE2kN. Other data that support the findings of this study are available from the corresponding authors upon request.

## Additional information

**How to cite this article:** Perez-Balderas, F. *et al*. Covalent assembly of nanoparticles as a peptidase-degradable platform for molecular MRI. *Nat. Commun.* **8**, 14254 doi: 10.1038/ncomms14254 (2017).

**Publisher's note:** Springer Nature remains neutral with regard to jurisdictional claims in published maps and institutional affiliations.

## Supplementary Material

Supplementary Information Supplementary Figures, Supplementary Tables, Supplementary Note, Supplementary Methods and Supplementary References

Supplementary Movie 1 Particle degradation by macrophages. Time-course montage of particle degradation by the murine macrophage cell line RAW264.7 obtained by live-cell confocal imaging. Fluorescent particle degradation was imaged over 12 hours taking an image every 2 minutes, during which time the number of visible particles decreased markedly.

Supplementary Movie 2 MRI of mouse injected intracerebrally with Il-1b and intravenously with aVCAMAF488-mMPIO. Serial in vivo T2*-weighted coronal images of mouse brain taken from a 3D gradient echo data set with ~90 mm isotropic resolution. This mouse received intrastriatal injection of 20 ng IL-1b in 1 ml saline 3 h prior to intravenous injection of aVCAM-AF488-mMPIO (4 mg iron per kg body weight). Intense low signal areas (i.e. black) on the left side of the brain reflect the specific retention of MPIO on acutely activated vascular endothelium with virtually absent contrast effect in the contra-lateral control hemisphere.

Supplementary Movie 3 MRI of mouse injected intracerebrally with Il-1b and intravenously with IgGAF488-mMPIO. Serial in vivo T2*-weighted coronal images of mouse brain taken from a 3D gradient echo data set with ~90 mm isotropic resolution. This mouse also received intrastriatal injection of 20 ng IL-1b in 1 ml saline 3 h prior to intravenous injection of IgG-AF488-mMPIO (4 mg iron per kg body weight).

## Figures and Tables

**Figure 1:**
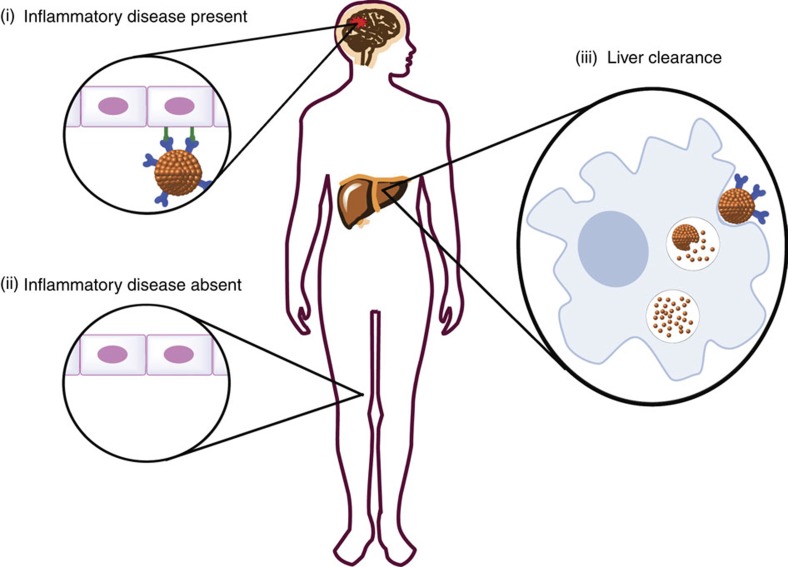
Molecular basis of mMPIO as a targeted MRI contrast agent. Intravenously injected targeted mMPIO bind to their target on the diseased endothelial surface (i), but do not bind to healthy endothelium (ii). The unbound mMPIO are rapidly cleared from blood. Thus, negligible background contrast effects are evident with mMPIO shortly after injection. mMPIO are efficiently taken up by macrophages (iii), and after internalization and fusion to lysosomes the internal peptide linkers are degraded. mMPIO are represented as brown sphere conglomerates, iron oxide nanoparticles are represented as brown spheres, targeting agents are represented in dark blue and endothelial surface disease markers are shown in green.
