# Supplementary material for: Covalent assembly of nanoparticles as a peptidase-degradable platform for molecular MRI
Source: Nat Commun. 2017 Feb 15;8:14254. doi: 10.1038/ncomms14254 (PMC5316865; doi:10.1038/ncomms14254)
Supplement: Supplementary Information — Supplementary Figures, Supplementary Tables, Supplementary Note, Supplementary Methods and Supplementary References [file ncomms14254-s1.pdf]

Supplementary Notes

Supplementary Note 1. A strategic comparison of mMPIO, MPIO and SPIO/USPIO

The R_2 effect of the mMPIO may be similar to that of SPIO for the same amount of iron, but per particle the R_2 effect for mMPIO is ~ 1000-fold greater than for SPIO (see **Supplementary Table 8**). This is an important comparison to make when considering relative contrast effects, since there are only a finite number of binding sites (for example VCAM-1 density expression in TNFα stimulated HUVEC cells[1] is 16 molecules/μm^2), using bigger particles means a bigger 'payload' of magnetic iron is delivered to each site of inflammation. Combined with the reduction in background signal (due to shorter blood half-life and lower level of non-specific retention), this results in a far greater signal-to-noise-ratio for the constructs. On this basis, the mMPIO clearly show a marked contrast benefit over SPIO and USPIO, which makes our approach very appealing.

Current, exciting pre-clinical models using MPIOs suggest that this approach will also be useful in cerebral inflammation,[2] cerebral malaria,[3] multiple sclerosis,[4] arterial thrombosis,[5,6] renal ischemia reperfusion imaging,[7] cerebral ischemia reperfusion imaging,[8] metastatic cancer,[9] cancer angiogenesis (in review); atherosclerosis,[10,11] and plaque rupture,[12] aortic aneurysm,[13] and myocardial ischemia reperfusion.[14]

Despite these advantages, current large/microparticle systems suffer from non-degradation and after some period of time aggregation leading to severe side effects, such as mechanical organ (lung, liver) retention.[15,16] Furthermore, it should be noted that Kupfer cells clear larger particles much more rapidly than nanoparticles and route these phagocytosed particles to vesicles of which the lysosomal fusion kinetics are greatly enhanced.

The targeted degradable microparticles (mMPIO) described here exhibit the beneficial properties of microparticles without such disadvantages; no untoward toxic effects from organ accumulation were observed during *in vivo* experiments. Thus, all of these models that currently use only MPIO are waiting

for the translational capacity that the mMPIO method may deliver through its degradability. In this way the advantages of SPIO and MPIO are combined in one system.

Supplementary Figures

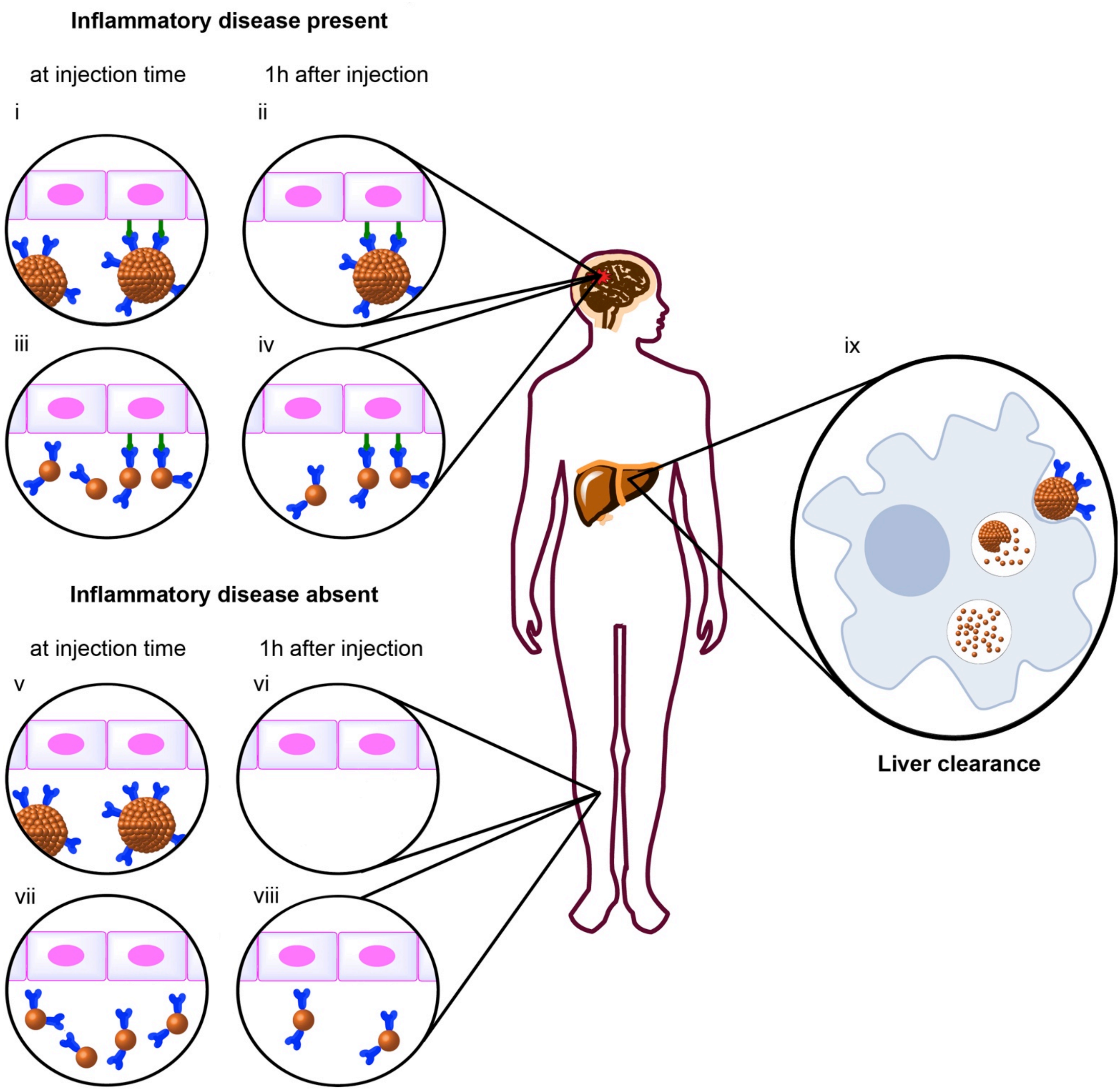

Supplementary Figure 1. Schematic to illustrate the molecular basis of the mMPIO as a targeted MRI contrast agent.

Intravenously injected targeted mMPIO bind to their target on the diseased endothelial surface (i), but do not bind to healthy endothelium (v). The unbound mMPIO are rapidly cleared from blood (ii and vi). Thus, negligible background contrast effects are evident with mMPIO shortly after injection. mMPIO are efficiently taken up by macrophages (ix), and after internalisation and fusion to lysosomes the internal peptide linkers are rapidly degraded to yield nanoparticles that are further degraded as widely described.

In contrast targeted USPIO are slowly cleared from blood, which increases the background level during MRI (iii, iv, vii, viii). USPIO are represented as brown spheres, mMPIO are represented as brown sphere conglomerates, iron oxide nanoparticles are represented as brown spheres, targeting agents are represented in dark blue and endothelial surface disease markers are shown in green. The different components in the figure are only representations and are not in scale.

Particle synthesis

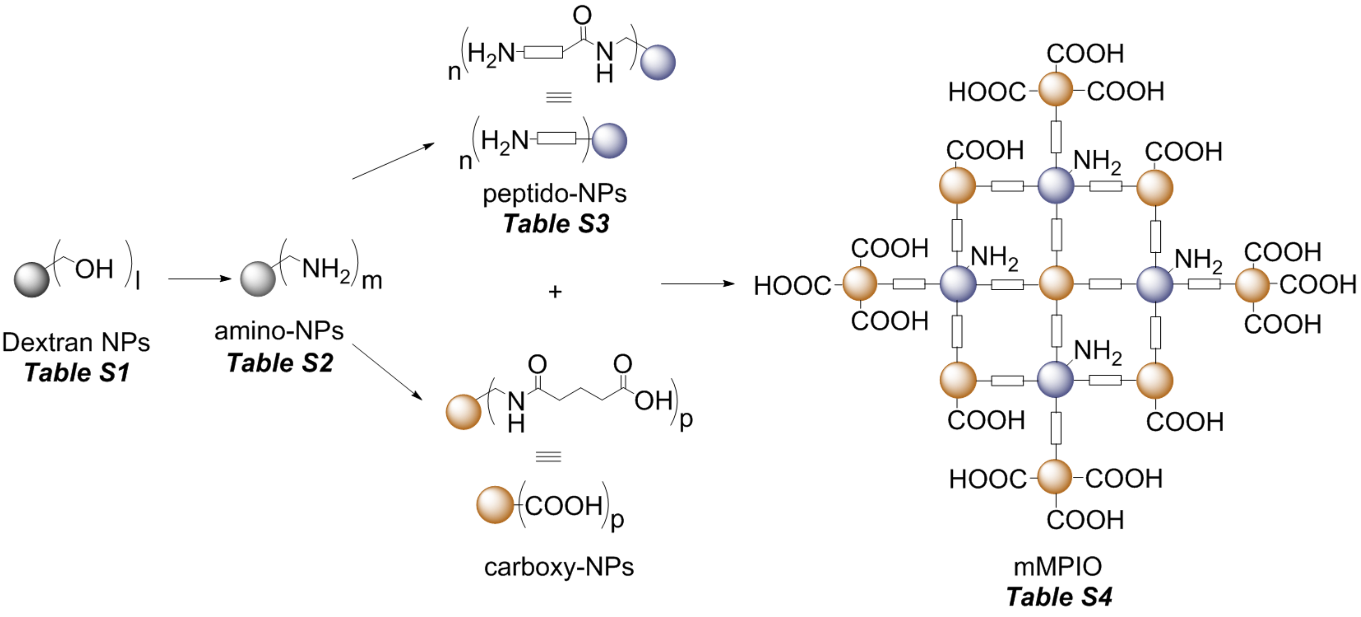

Supplementary Figure 2. Correspondence between particles and Supplementary Tables 1-4.

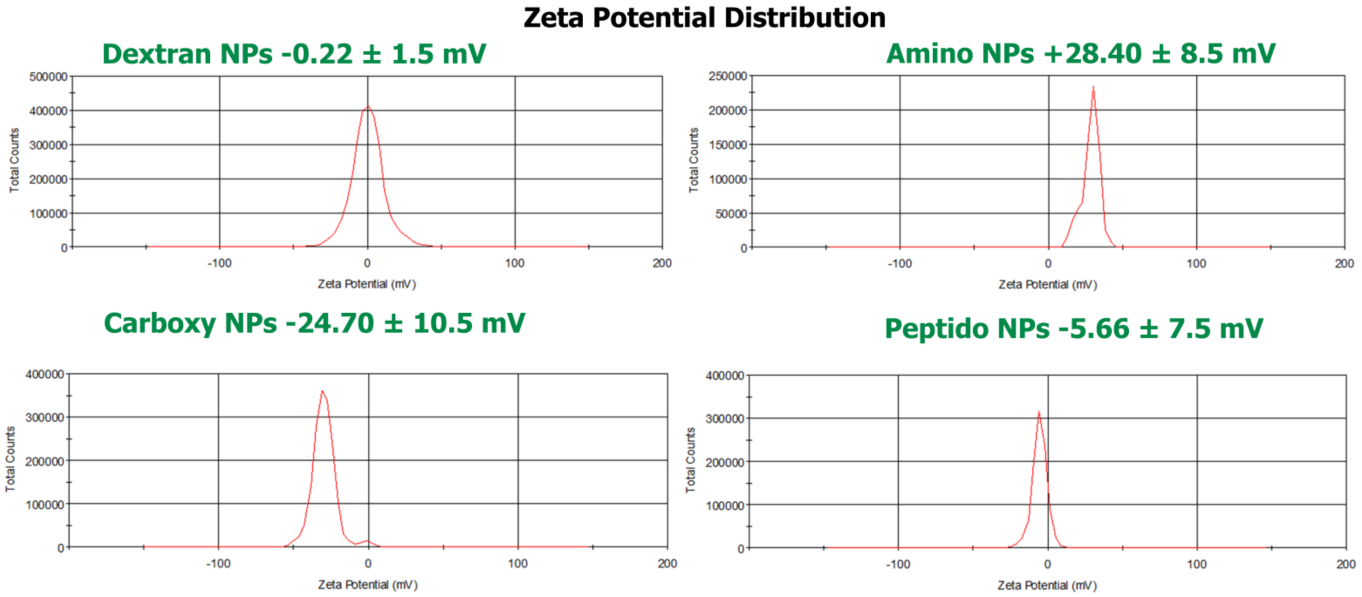

Supplementary Figure 3. Representative zeta potential mesurements.

Results are given for dextran-covered nanoparticles, amino-NPs, carboxy-NPs and peptido-NPs. The data reveal a significant change in the particle outer–surface charge confirming successful surface modification. Measurements were recorded in 10 mM sodium phosphate buffer at pH 7.0. All data were recorded in triplicate (mean±s.d, n=9).

Degradation studies of the peptidic linker

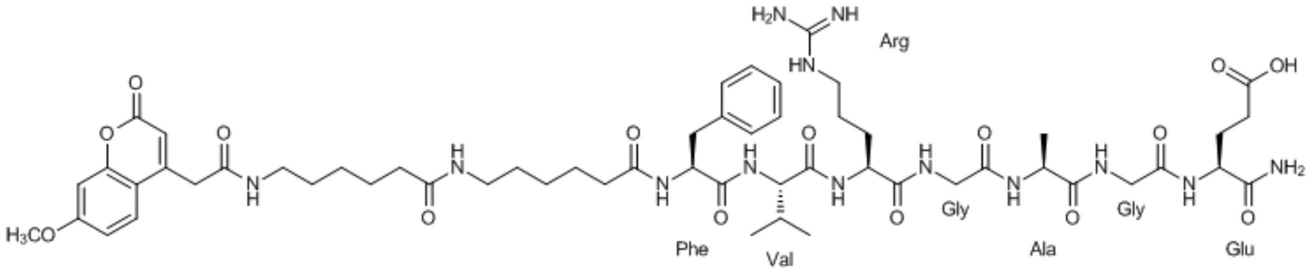

1

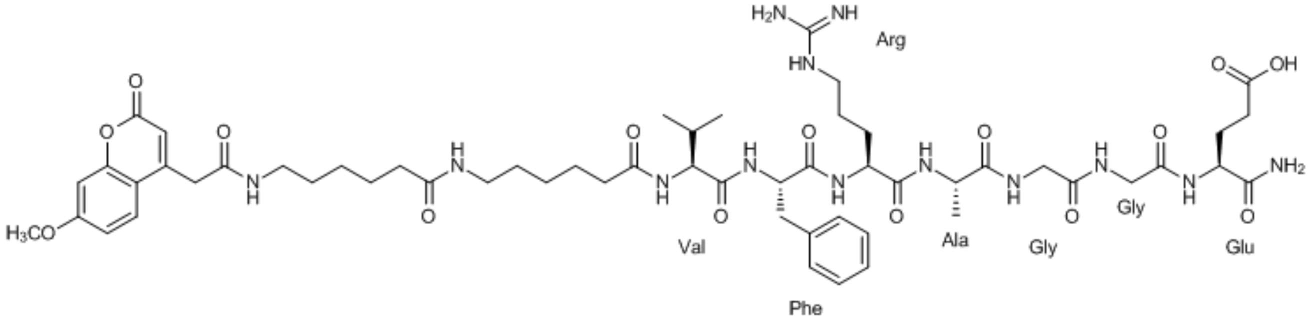

2

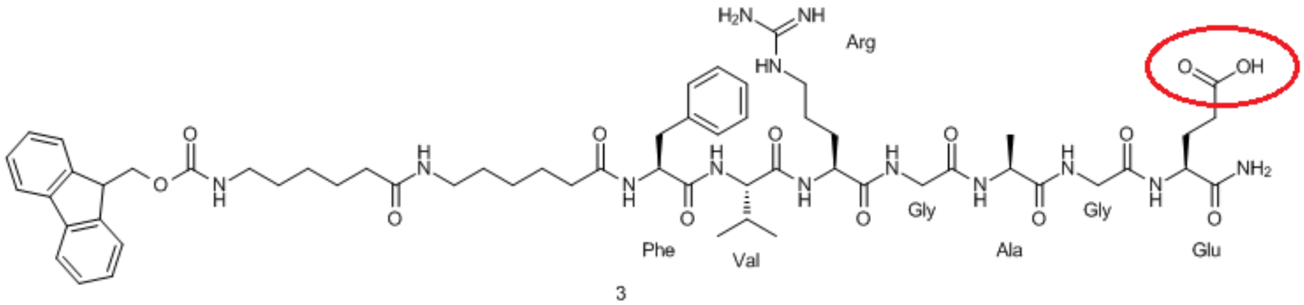

3

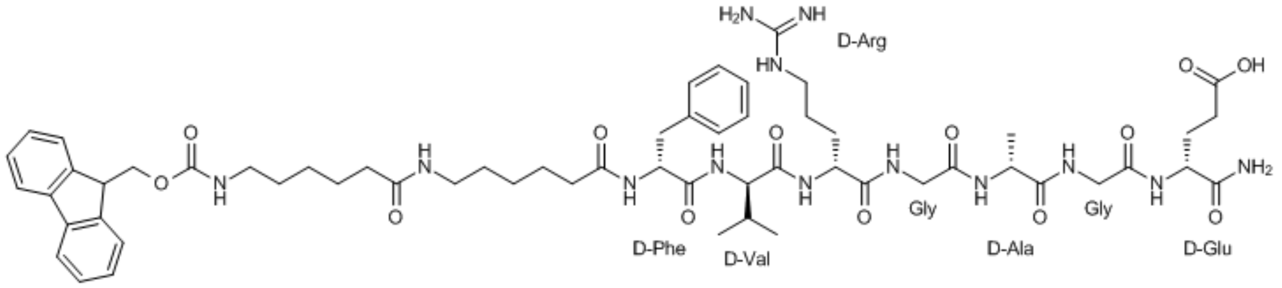

4

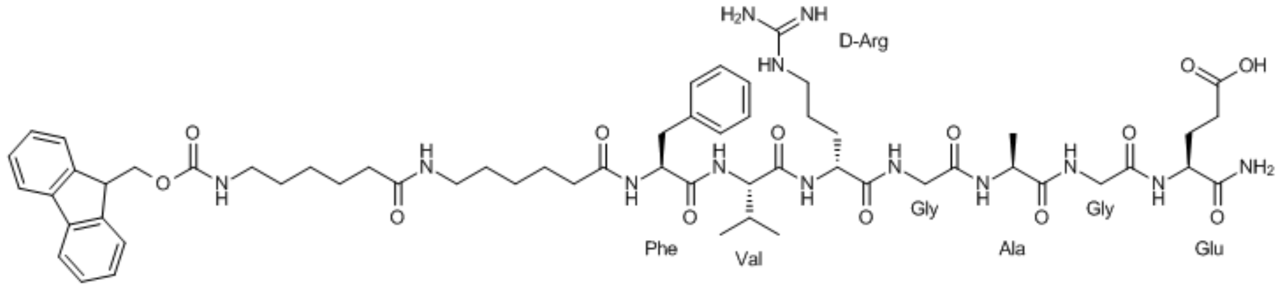

5

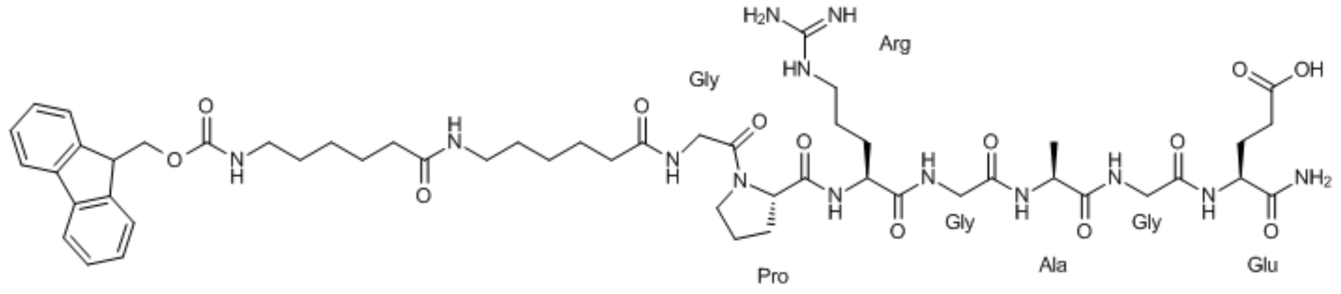

6

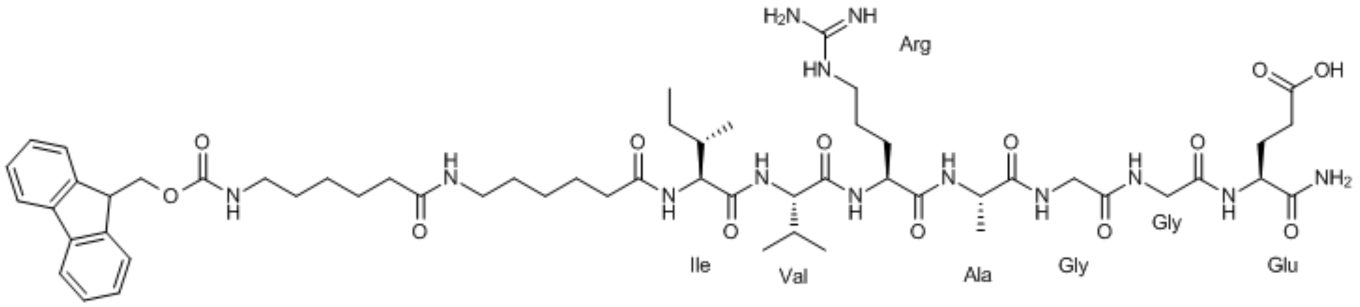

7

Supplementary Figure 4. **Structure of peptides 1-7.**

Sequence of peptides **1** (Mca-XXFVRGAGE-NH_2), **2** (Mca-XXVFRAGGE-NH_2), **3** (Fmoc-XXFVRGAGE-NH_2), **4** (Fmoc-XXfvrGaGe-NH_2), **5** (Fmoc-XXFVrGAGE-NH_2), **6** (Fmoc-XXGPRGAGE-NH_2), **7** (Fmoc-XXIVRAGGE-NH_2) (X= 6-aminohexanoic acid, Mca = 7-methlycoumarin-4-acetyl, upper case letters denote L-amino acids and lower case letters denote D-amino acids). The carboxylic acid function that reacts with the amine-functionalized dextran covered iron oxide nanoparticles (NPs) is highlighted by a red ellipse. Peptide **3** is the peptide integrated in the mMPIO.

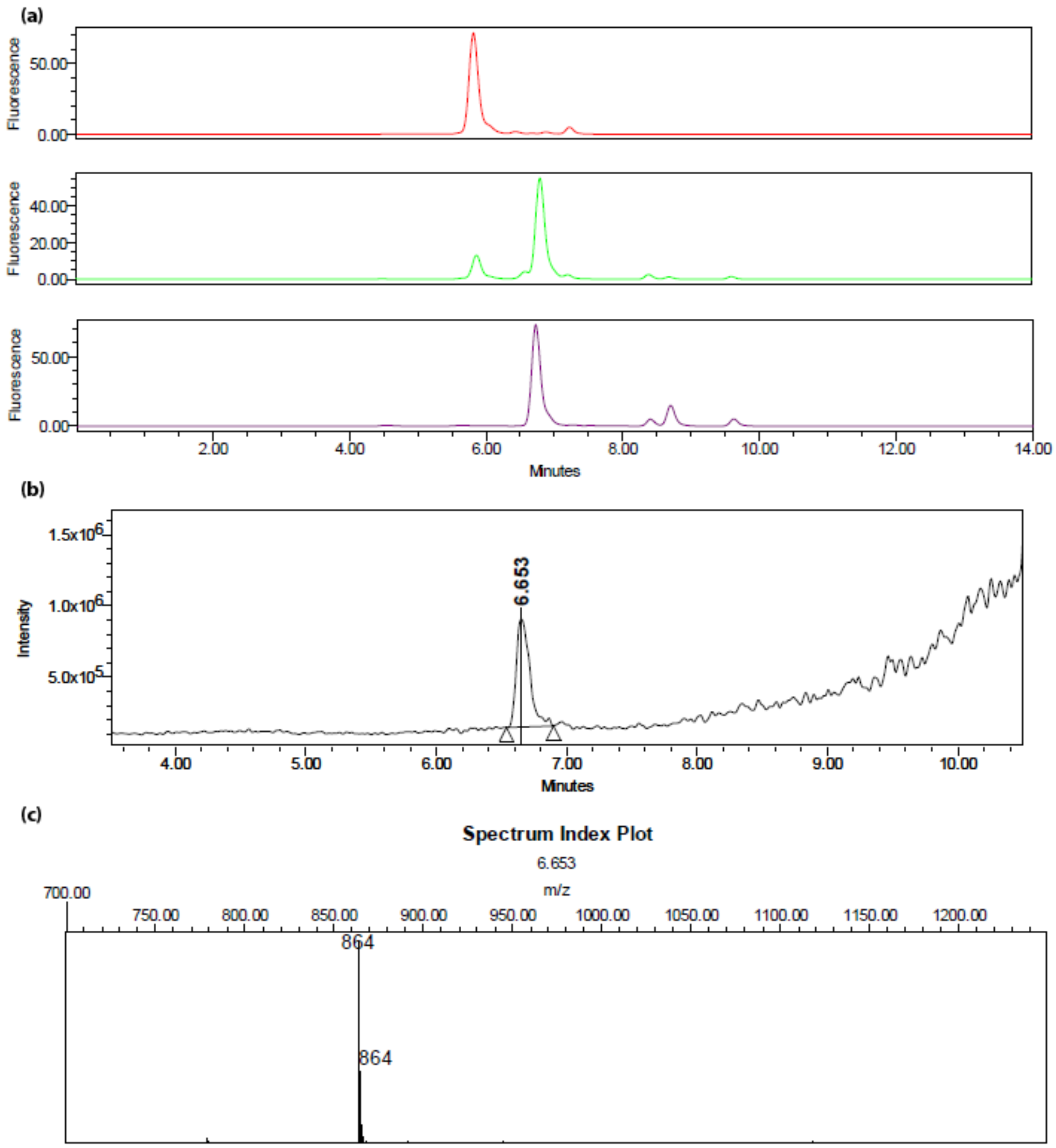

Supplementary Figure 5. The degradation of peptide 1 by cathepsin B.

(a) HPLC chromatogram of the degradation of peptide **1** with 20 nM of cathepsin B at times 0 (red), 1 (green) and 8 hours (dark magenta); (b) MS chromatogram of the degradation of peptide **1** with cathepsin B after 8 hours of reaction and (c) MS spectra of peak extracted from MS chromatogram at time 6.6 min. The uncleaved peptide elutes at 5.8 minutes, the main fluorescent cleaved peptide elutes at 6.7 minutes with a $[M+H]^+$ = 863.38, which corresponds to a peptide with the sequence Mca-AhxAhxPheValArg-OH. Few more fluorescent cleaved peptides elute at longer retention times.

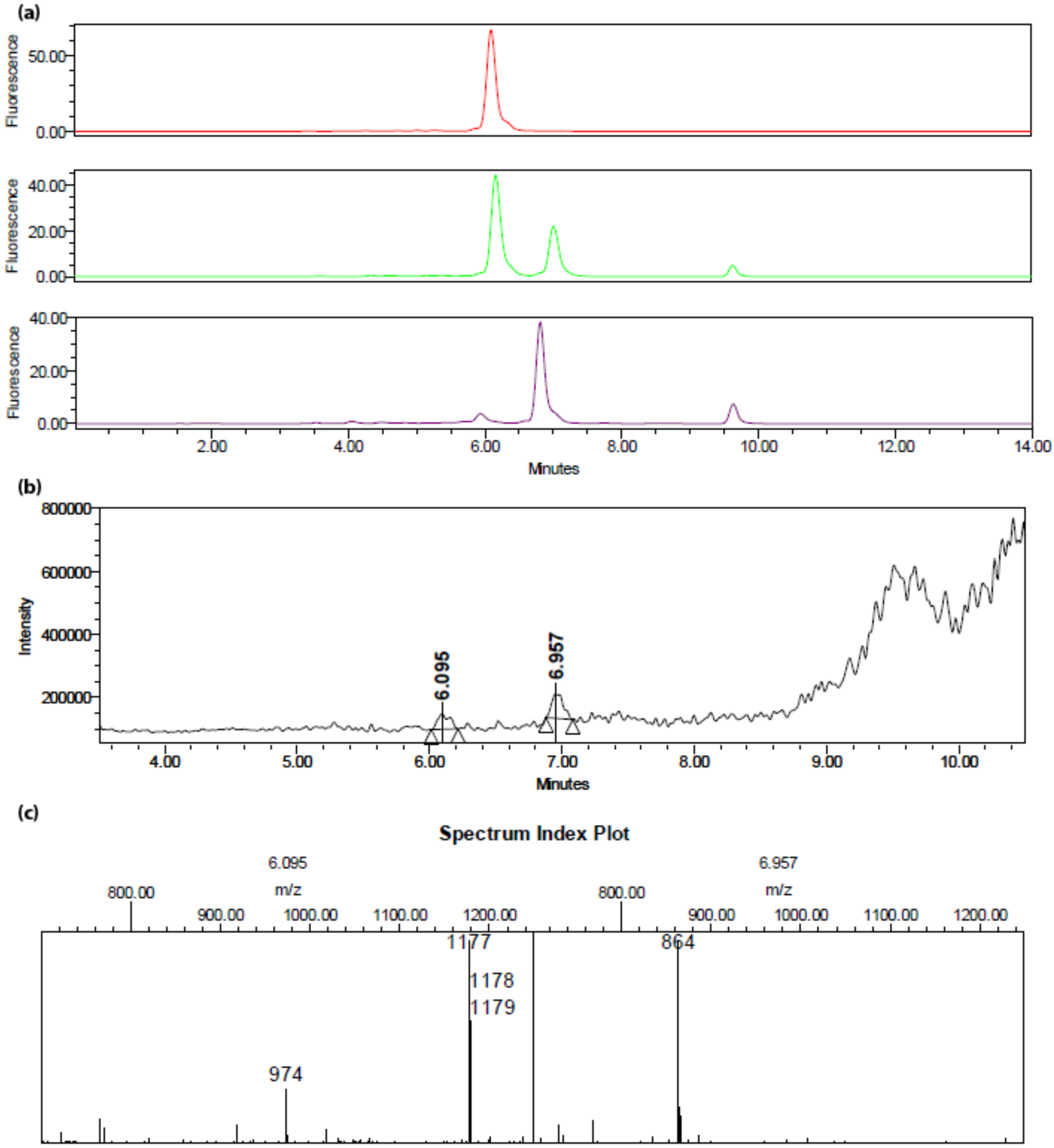

Supplementary Figure 6. The degradation of peptide 2 by cathepsin B.

(a) HPLC chromatogram of the degradation of peptide **2** with 20 nM of cathepsin B at times 0 (red), 1 (green) and 8 hours (dark magenta), (b) MS chromatogram of the degradation of peptide **2** with cathepsin B after 1 hour of reaction and (c) MS spectra of peaks at times 6.1 min (left) and 7.0 min (right) extracted from MS chromatogram. The uncleaved peptide elutes at 6.1 minutes, the main fluorescent cleaved peptide elutes at 7.0 minutes with a $[M+H]^+$ = 863.63, which corresponds to a peptide with the sequence Mca-AhxAhxValPheArg-OH. A minor fluorescent cleaved peptide elutes at 9.6 minutes.

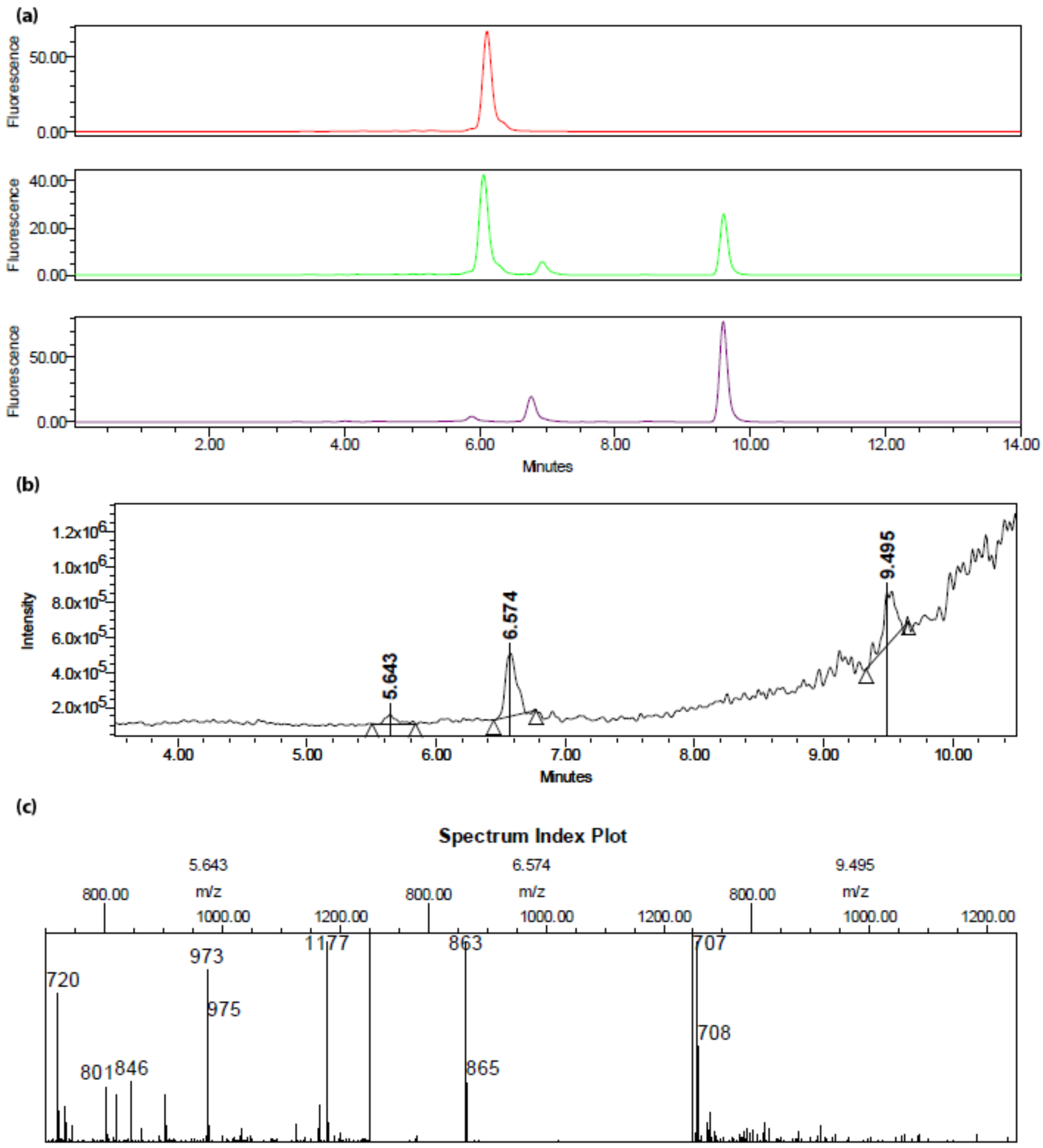

Supplementary Figure 7. The degradation of peptide 1 by cathepsin L.

(a) HPLC chromatogram of the degradation of peptide **1** with 3 nM of cathepsin L at times 0 (red), 1 (green) and 8 hours (dark magenta). (b) MS chromatogram of the degradation of peptide **1** with 3 nM of cathepsin L after 8 hours of reaction and (c) MS spectra of peaks extracted from MS chromatogram at times 5.6 min (left), 6.6 min (centre) and 9.5 min (right). The uncleaved peptide elutes at 5.6 minutes, a first fluorescent cleaved peptide elutes at 6.6 minutes with a $[M+H]^+$ = 863.44, which corresponds to a peptide with the sequence Mca-AhxAhxValPheArg-OH and an additional fluorescent cleaved peptide elute at 9.5 minutes with a $[M+H]^+$ = 707.46, which corresponds to the peptide Mca-AhxAhxValPhe-OH.

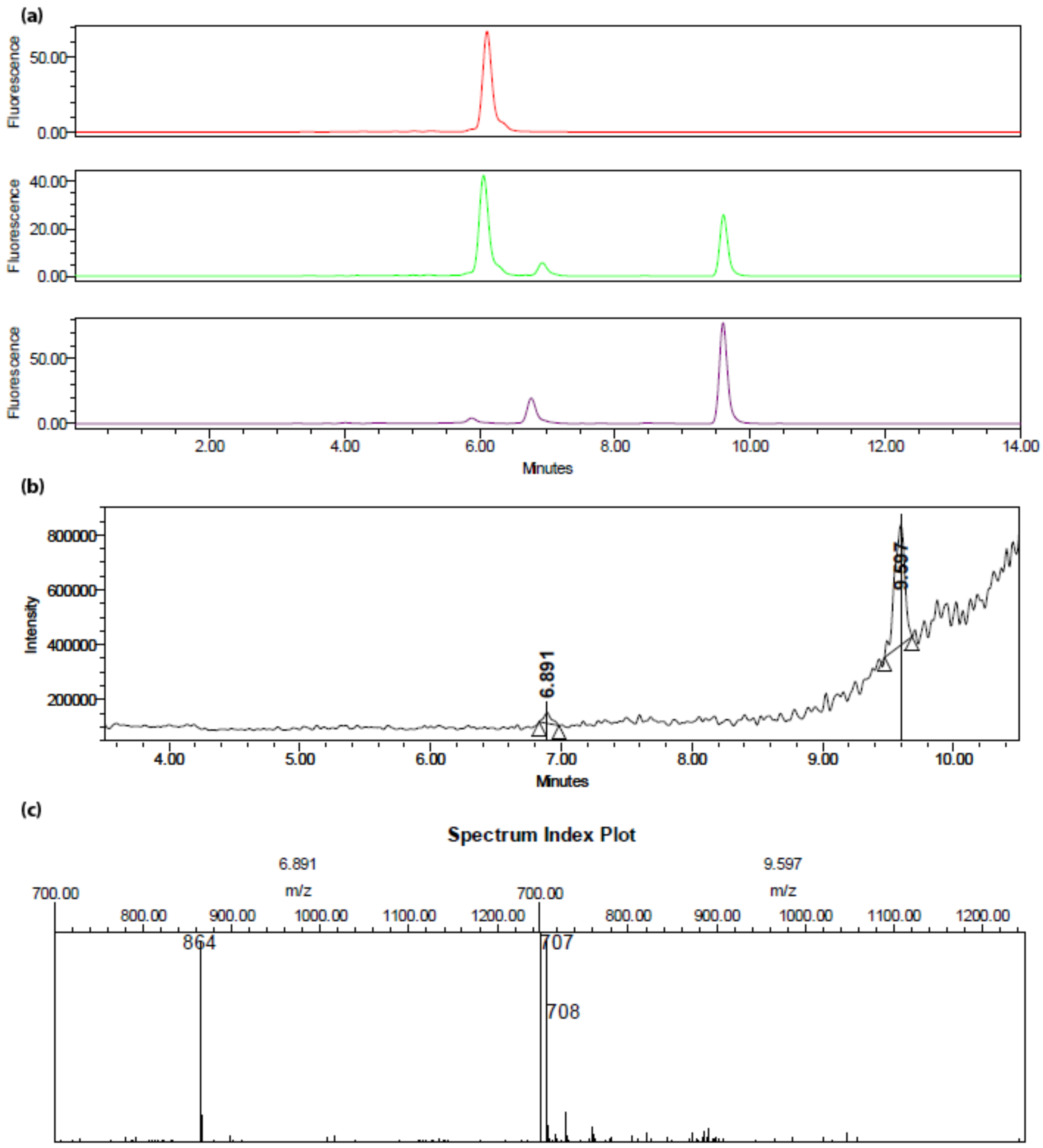

Supplementary Figure 8. The degradation of peptide 2 by cathepsin L.

(a) HPLC chromatogram of the degradation of peptide **2** with 3 nM of cathepsin L at times 0 (red), 1 (green) and 8 hours (dark magenta). (b) MS chromatogram of degradation of peptide 2 with cathepsin L after 4 hours of reaction and (c) MS spectra of peaks extracted from MS chromatogram at times 6.9 min (left) and 9.6 min (right).. The uncleaved peptide elutes at 5.8 minutes, a main fluorescent cleaved peptide elutes at 9.6 minutes with a [M+H]+ = 707.46, which corresponds to a peptide with the sequence Mca-AhxAhxPheVal-OH and an additional fluorescent cleaved peptide elutes at 6.9 minutes with a [M+H]+ = 863.44, which corresponds to a peptide with the sequence Mca-AhxAhxPheValArg-OH.

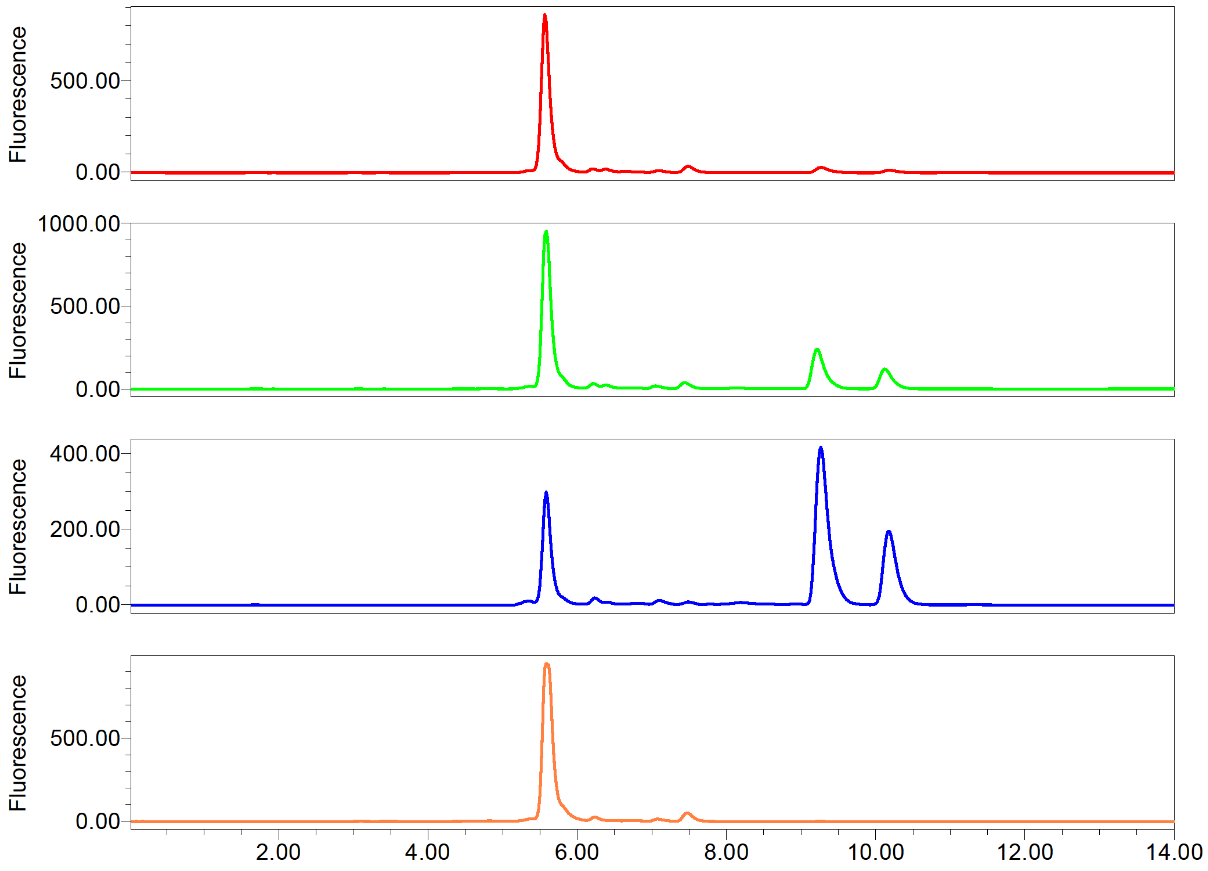

Supplementary Figure 9. Stability of the peptide 1 in human plasma.

HPLC chromatogram of the stability of peptide **1** in human plasma at times 1 (red), 6 (green) and 24 (blue) hours and in PBS at 24 hours (orange). This peptide is stable for the duration of the whole animal experiment.

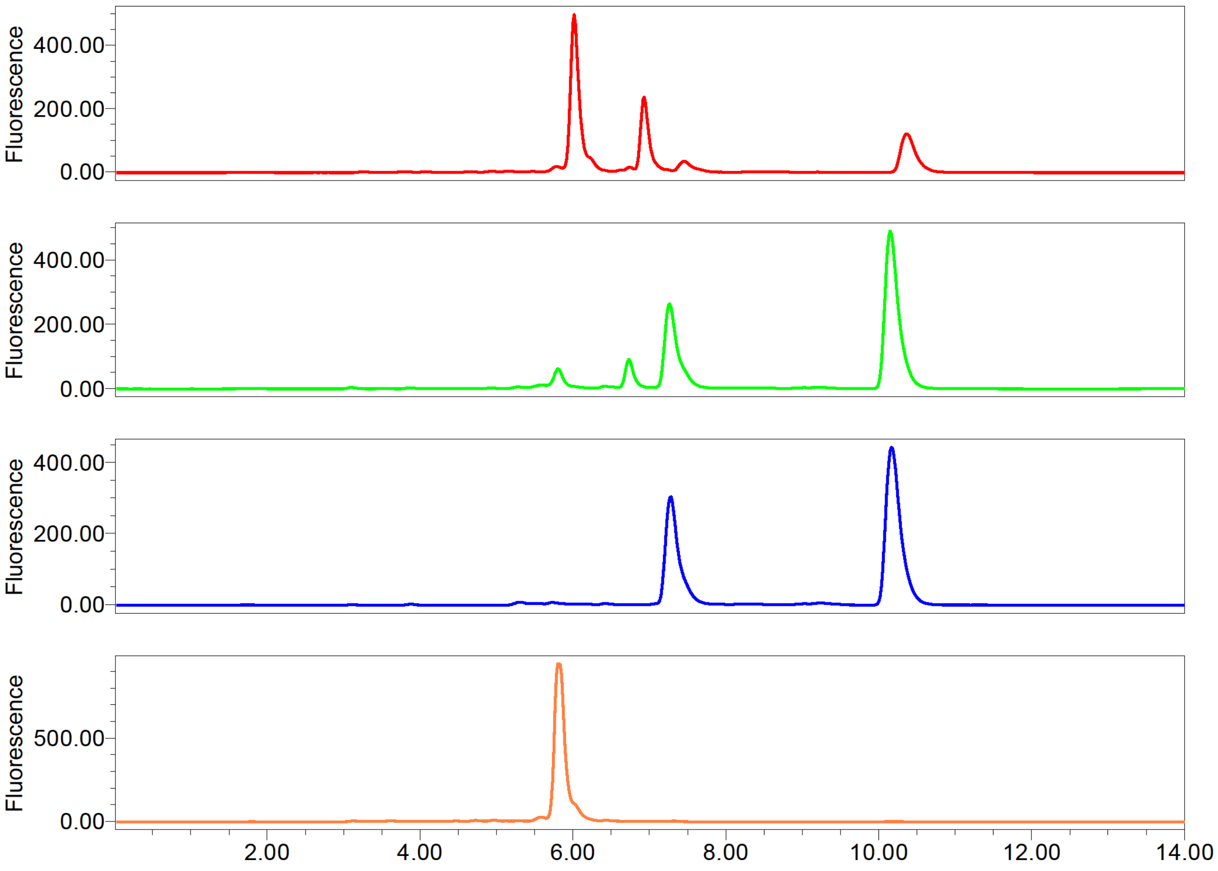

Supplementary Figure 10. Stability of the peptide 2 in human plasma.

HPLC chromatogram of the stability of peptide **2** in human plasma at times 1 (red), 6 (green) and 24 (blue) hours and in PBS at 24 hours (orange).

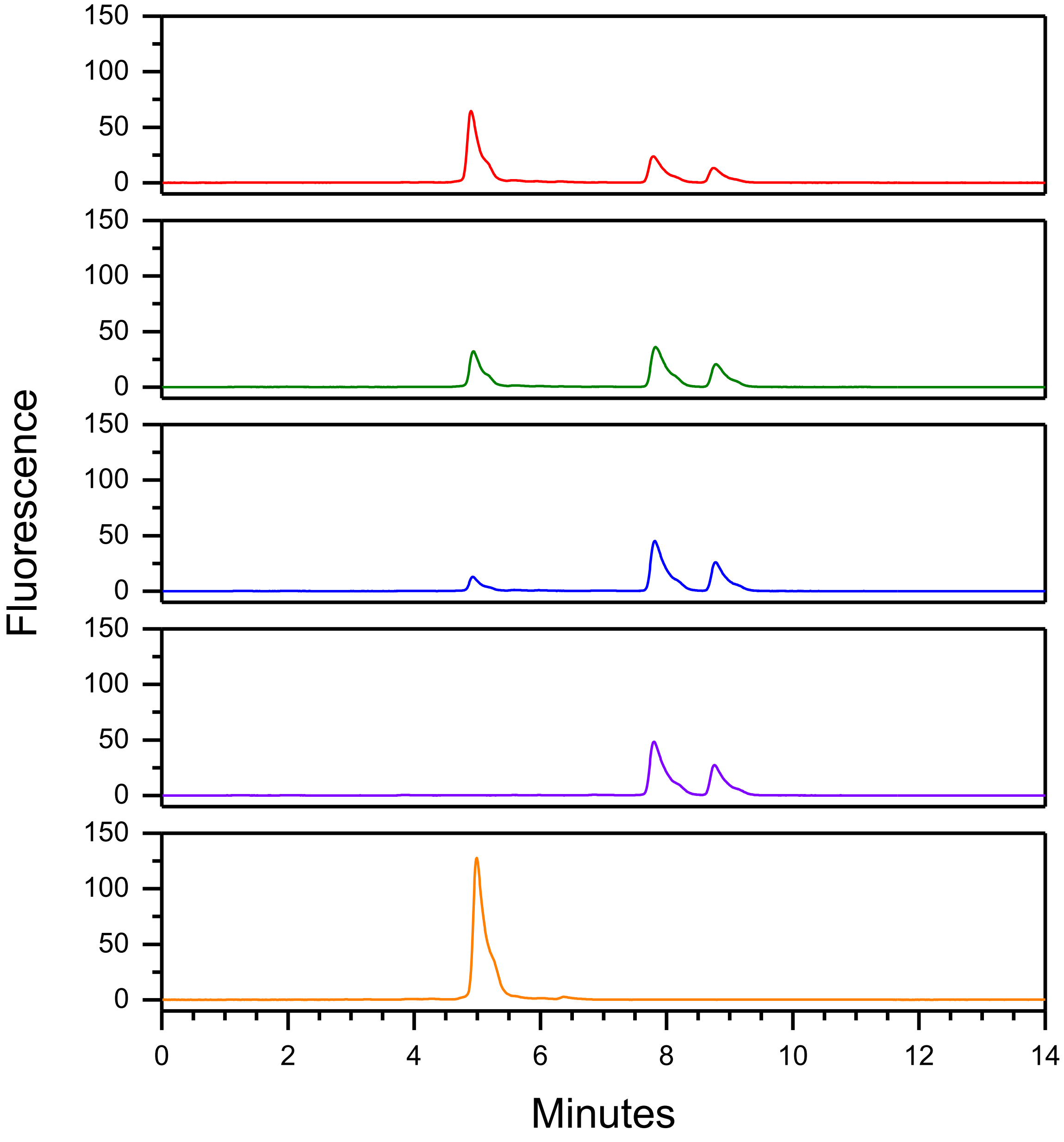

Supplementary Figure 11. Stability of peptide 1 in human serum.

HPLC chromatogram of the stability of peptide **1** in human serum at times 1h (red), 2h (green), 4h (blue) and 24h (violet), and peptide **1** in PBS at 24h (orange).

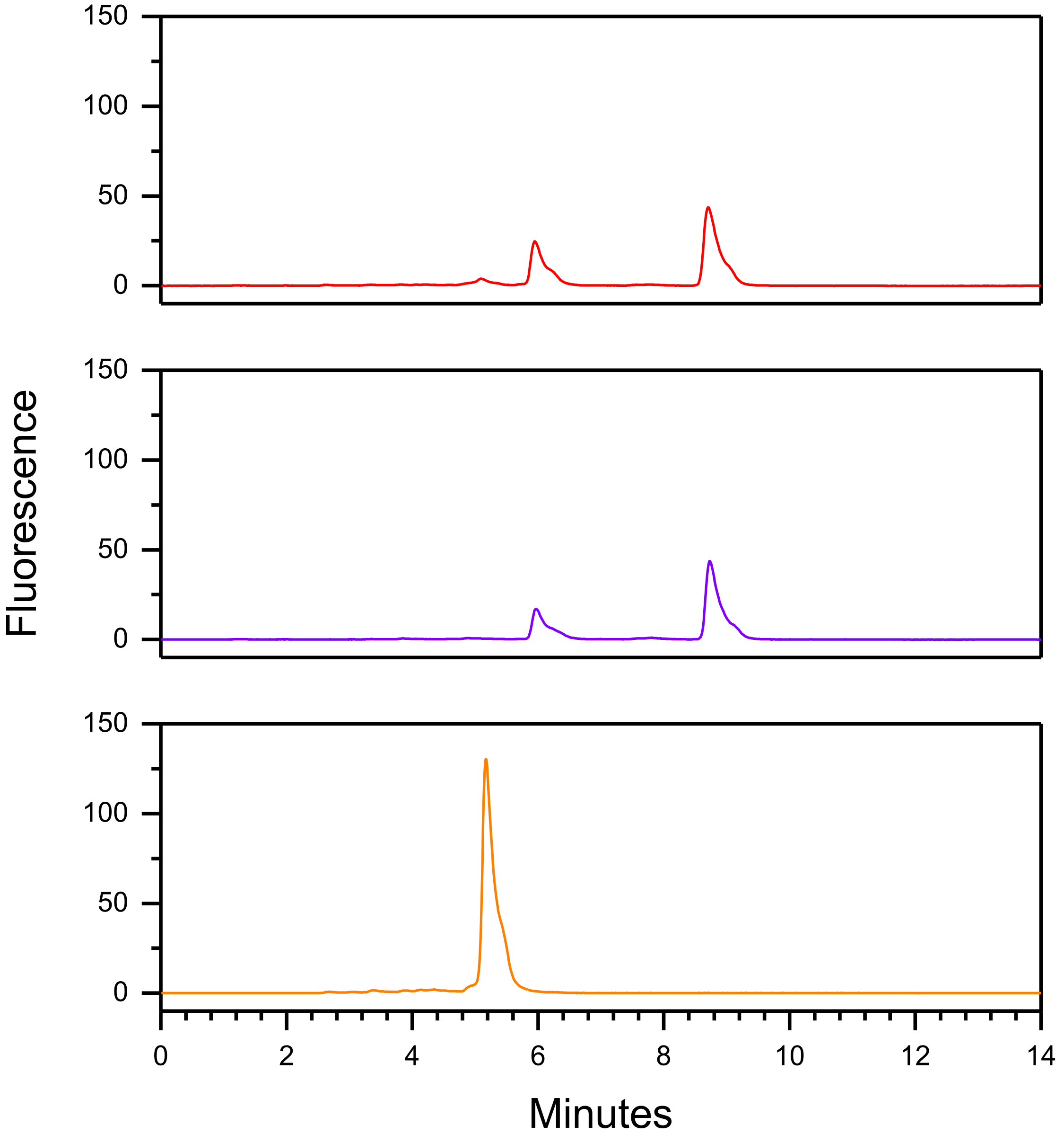

Supplementary Figure 12. Stability of peptide 2 in human serum.

HPLC chromatogram of the stability of peptide **2** in human serum at times 1h (red) and 24h (violet), and peptide **2** in PBS at 24h (orange).

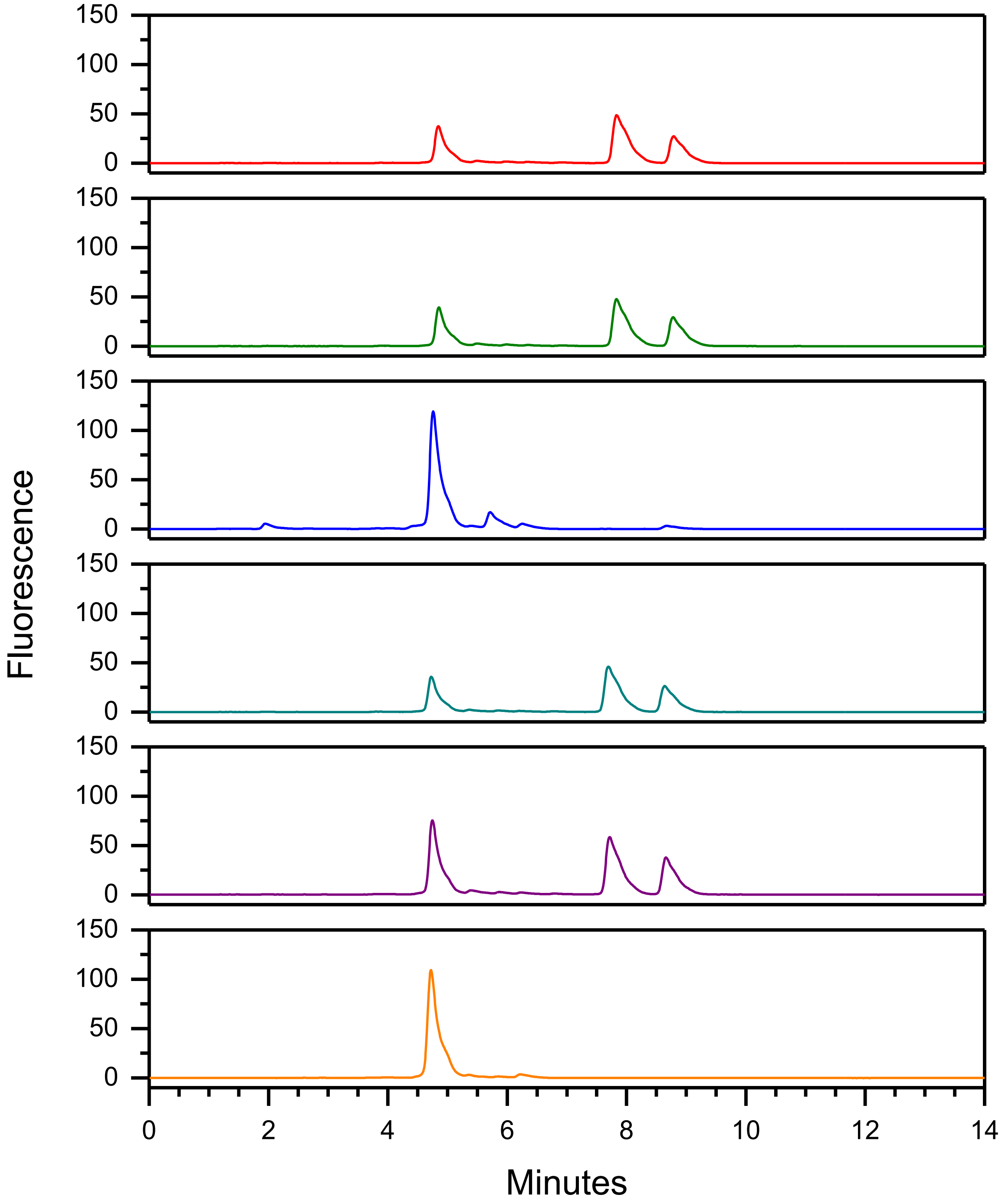

Supplementary Figure 13. Stability of peptide 1 in human serum with different inhibitors.

HPLC chromatogram of the stability of peptide **1** in human serum at the following conditions (from top to bottom): no additive added, 200 µg/mL hirudin, 10 mM EDTA, 10 IU/mL heparin, complete mini EDTA free cocktail, and serum pre-treated at 95 ºC for 10 min.

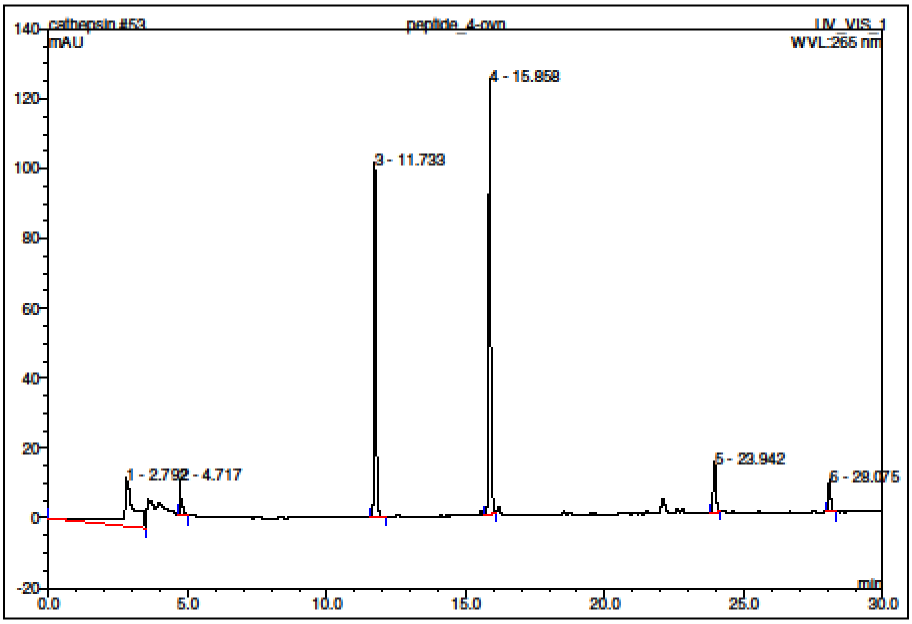

Supplementary Figure 14. The degradation of peptide 4 by cathepsin L after 17 h.

HPLC chromatogram; Peak at time 11.7 min corresponds to Cathepsin L and at time 15.9 min corresponds to uncleaved peptide **4**.

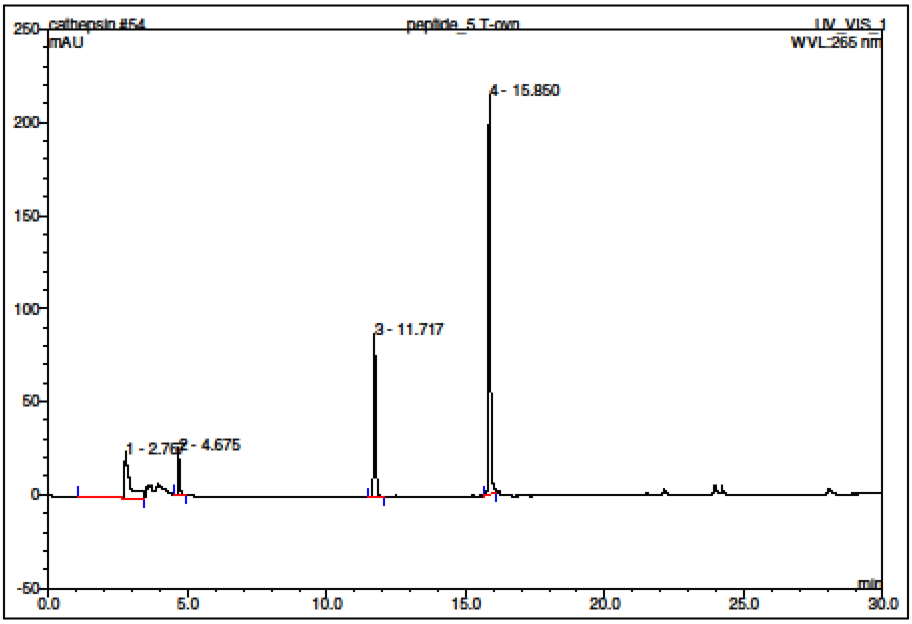

Supplementary Figure 15. The degradation of peptide 5 by cathepsin L after 17 h.

HPLC chromatogram; Peak at time 11.7 min corresponds to Cathepsin L and at time 15.9 min corresponds to uncleaved peptide **5**.

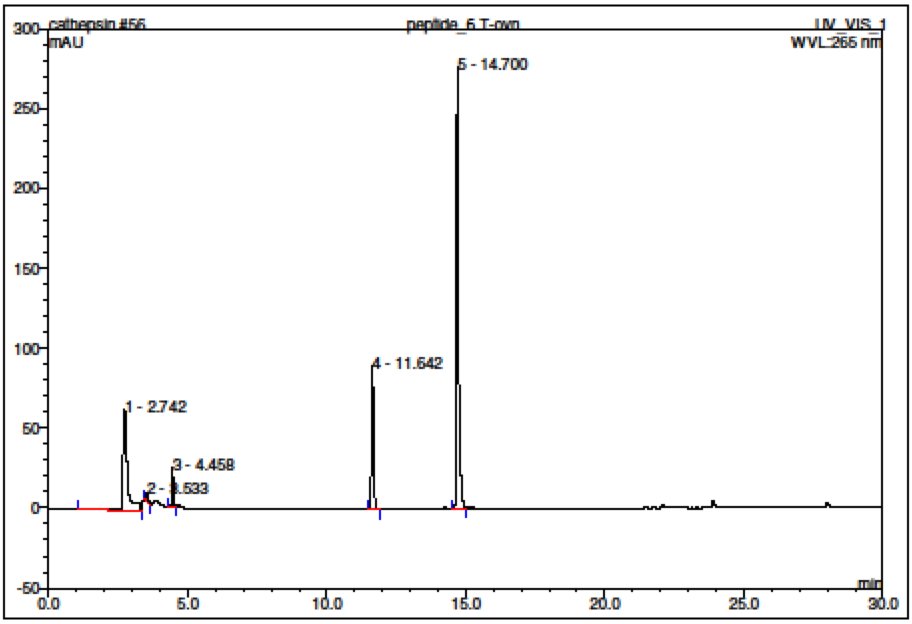

Supplementary Figure 16. The degradation of peptide 6 by cathepsin L after 17 h.

HPLC chromatogram; Peak at time 11.6 min corresponds to Cathepsin L and at time 14.7 min corresponds to uncleaved peptide **6**.

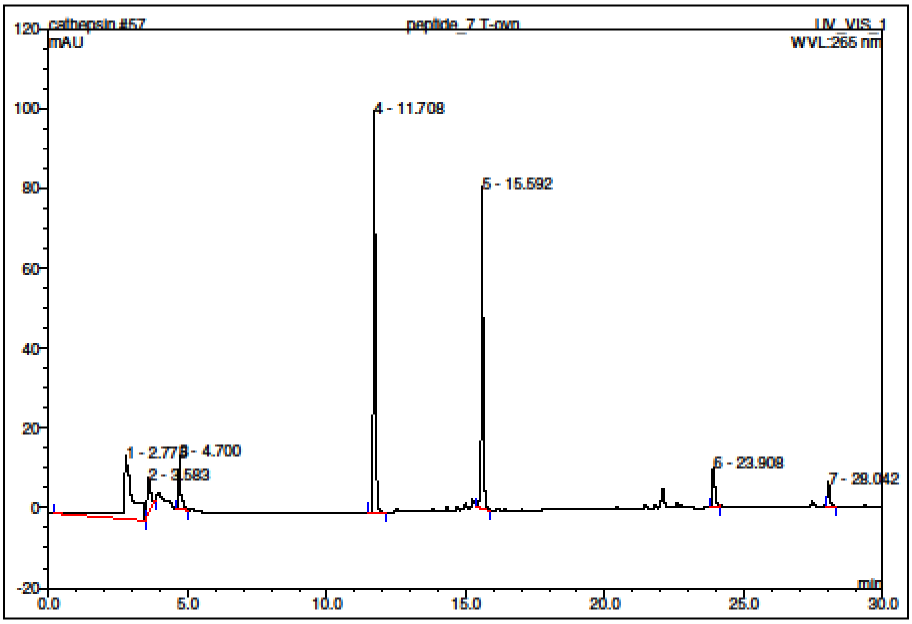

Supplementary Figure 17. The degradation of peptide 7 by cathepsin L after 17 h.

HPLC chromatogram; Peak at time 11.7 min corresponds to Cathepsin L and at time 15.6 min corresponds to uncleaved peptide **7**.

mMPIO physicochemical properties

(a)

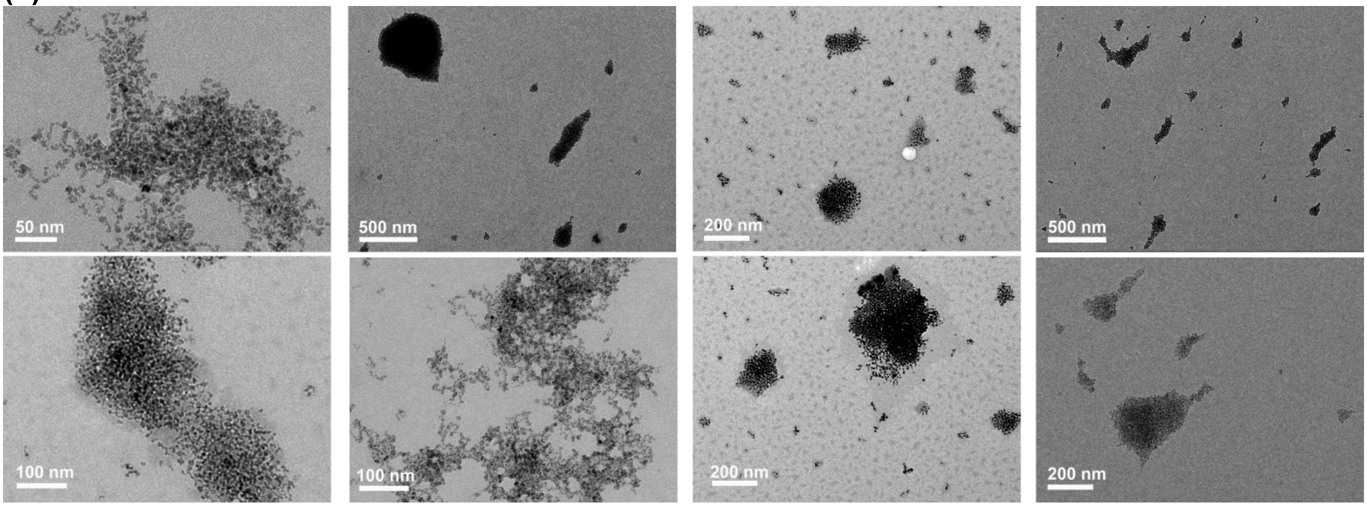

(b)

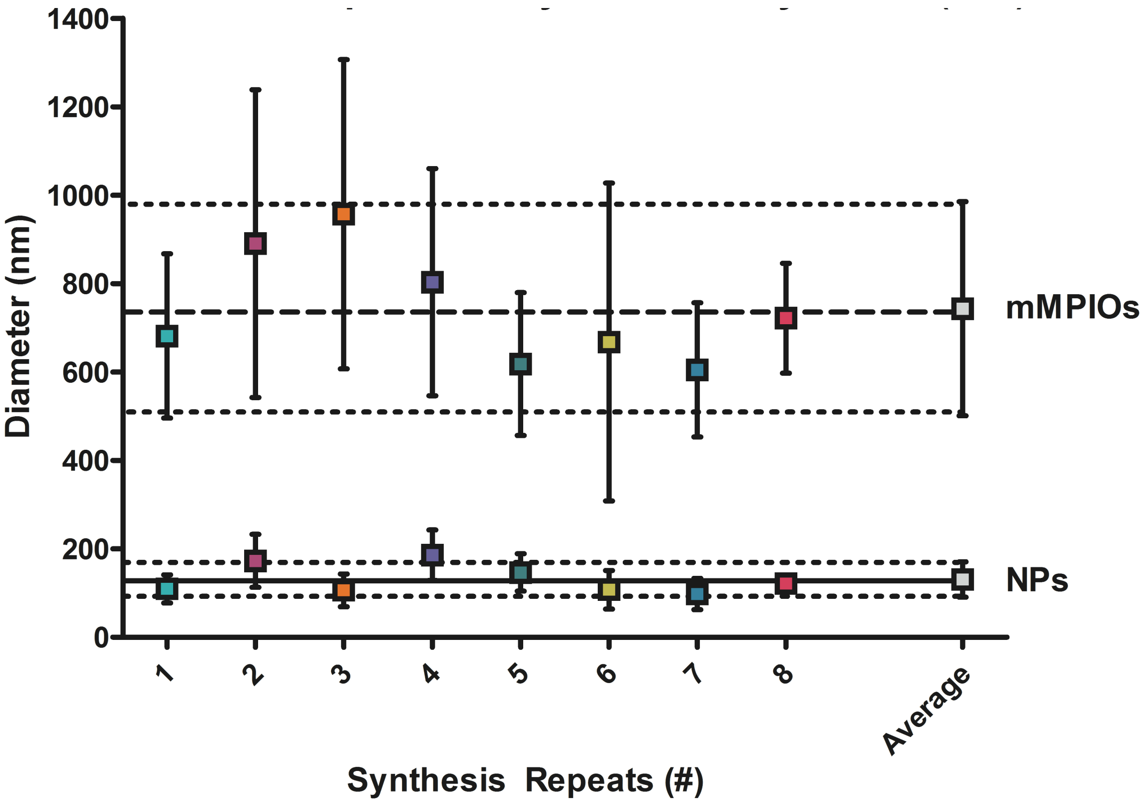

(c)

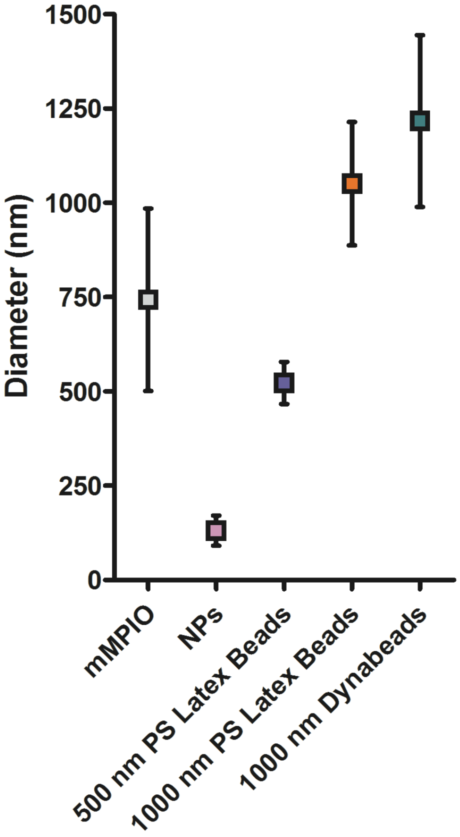

Supplementary Figure 18. Particle Morphology and Size Distribution Analyses

(a) TEM micrographs (unstained) taken of the crude reaction mixtures from different particle syntheses. The data show the mMPIOs assembled with some structural variance; however, larger particles appear to be pseudo-spherical. Scale bars 50-500 nm, as shown.

(b) Size and reproducibility of the mMPIO synthesis. NPs (building blocks) and mMPIO size distribution from crude reaction mixtures were measured by DLS (Zetasizer Nano ZSP) and analysed with Zetasizer Software (Malvern, version 7.11). The average resulting mean particle diameter is 743.01 ± 242.18 nm (mean±s.d, n=8) for the synthesized mMPIOs and 131.05 ± 40.01 nm (mean±s.d, n=8) for the NPs building blocks used. Each synthesis was measured at least 3 times and the standard deviation was plotted for each individual synthesis. The figure was generated with GraphPad Prism 5.01 software.

(c) Comparison of size, size distribution of commercial particles and mMPIOs as measured by DLS. Size distribution was measured by DLS (Zetasizer Nano ZSP) and analysed with Zetasizer Software (Malvern, version 7.11). Both commercial and mMPIOs were measured at least 3 times and the standard deviation was plotted for the particles. The figure was generated with GraphPad Prism 5.01 software.

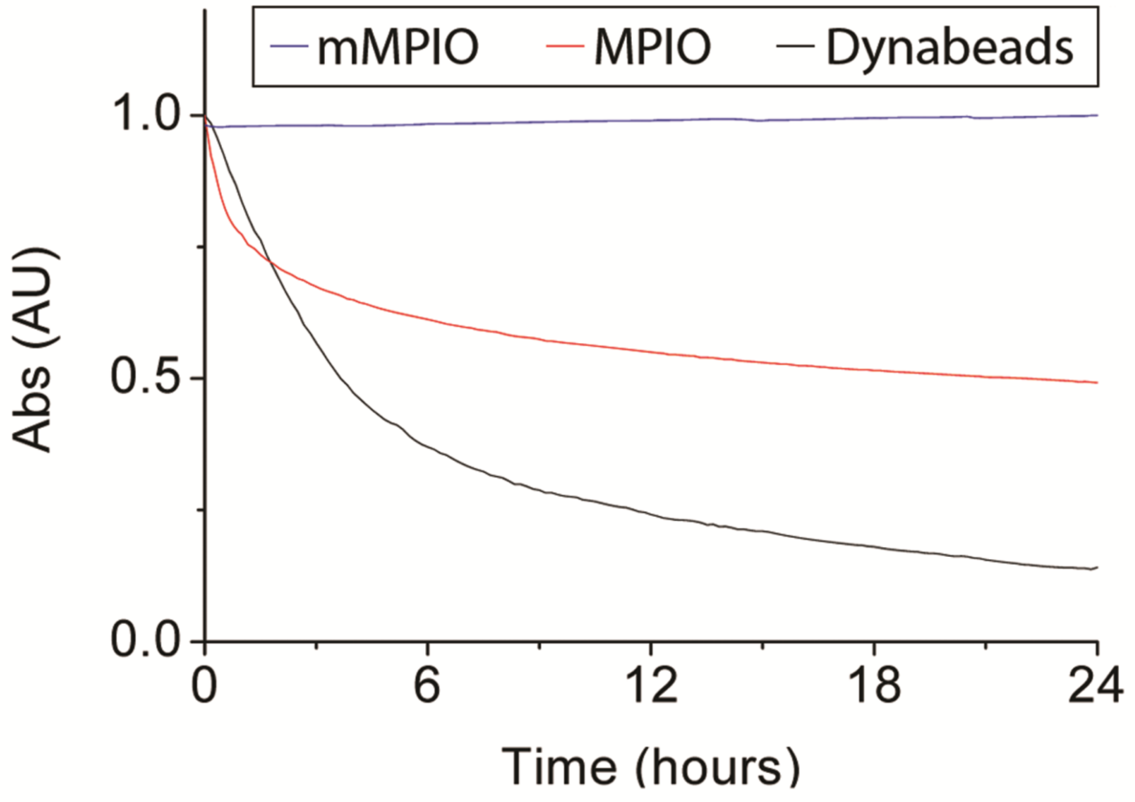

Supplementary Figure 19. Spectrophotometric determination of particle sedimentation.

Sample light absorption was measured at λ = 500 nm for 24 hours in a closed cuvette. Dynabeads rapidly sediment within few hours, MPIO sedimentation is slightly slower but appreciable finally mMPIO remain in solution during the time of the experiment with negligible particle sedimentation.

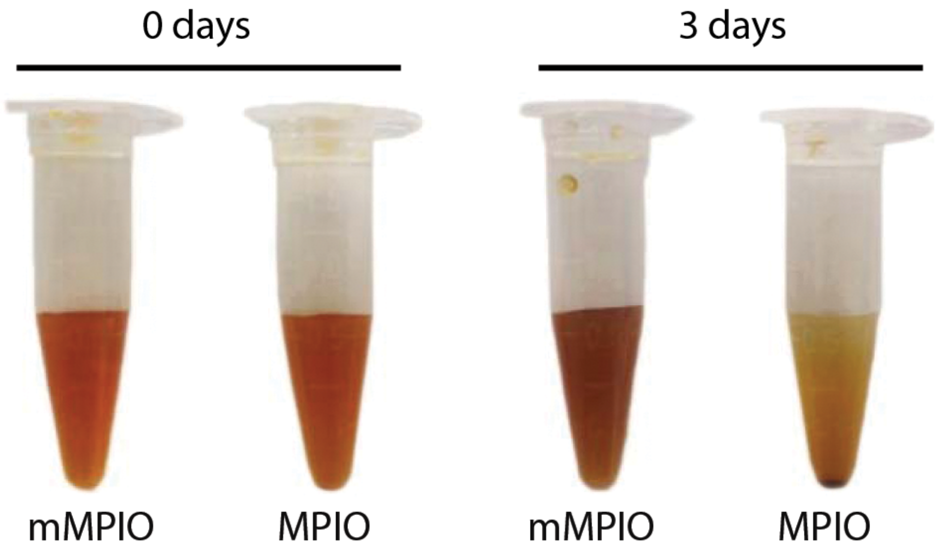

Supplementary Figure 20. mMPIO and MPIO sedimentation.

mMPIO and MPIO solutions immediately after vortexing and sonication (left). The large monomeric particles largely sediment after 3 days while the sedimentation was negligible in case of the mMPIO solution (right).

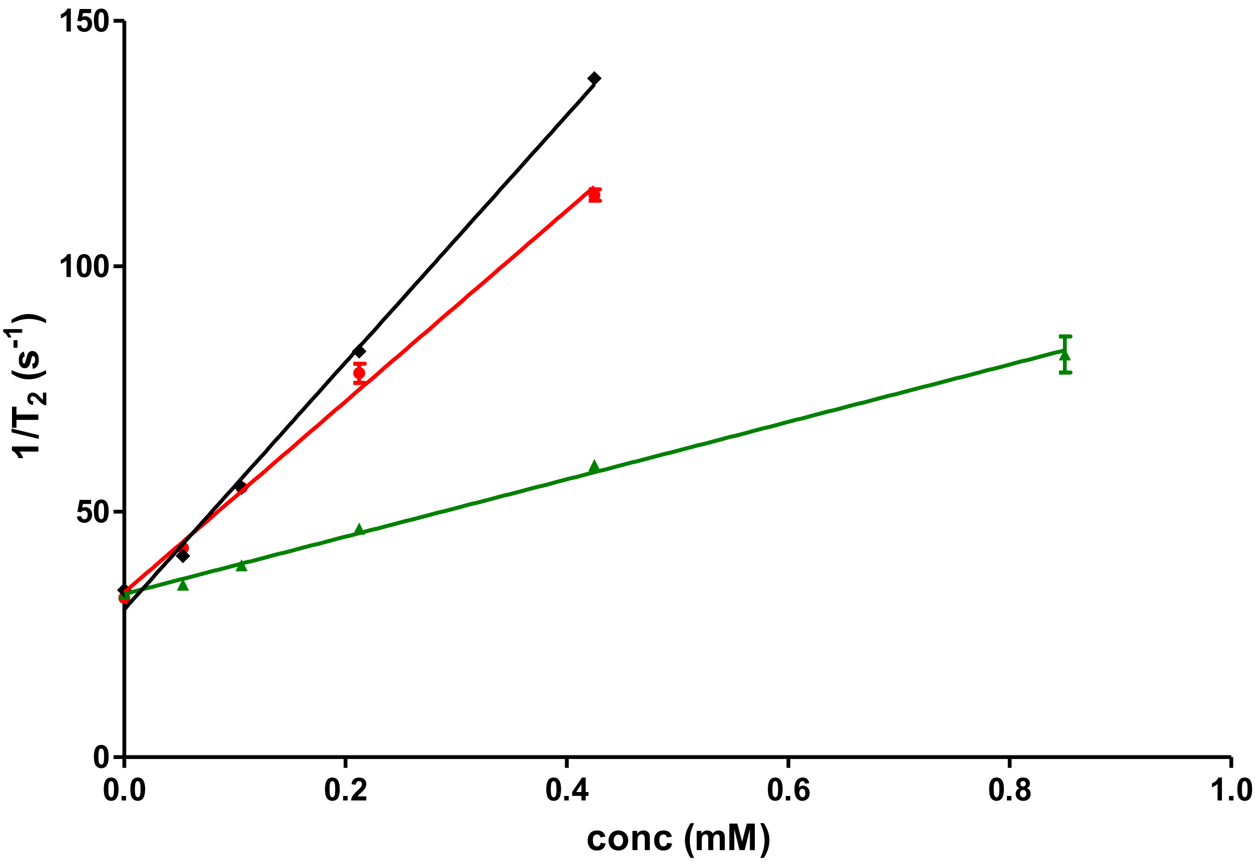

Supplementary Figure 21. T_2 relaxation rates of mMPIO, MPIO and Dynabeads at 4.7 T.

T_2 relaxivity plots for uniform dispersions of particles in a 6% agarose matrix. mMPIO (red circles) have a greater relaxivity (shown by a steeper slope) than Dynabeads (green triangles), but slightly lower than the MPIO (black diamonds). Errors are expressed as mean±s.d. of three samples.

IId. mMPIO degradation studies

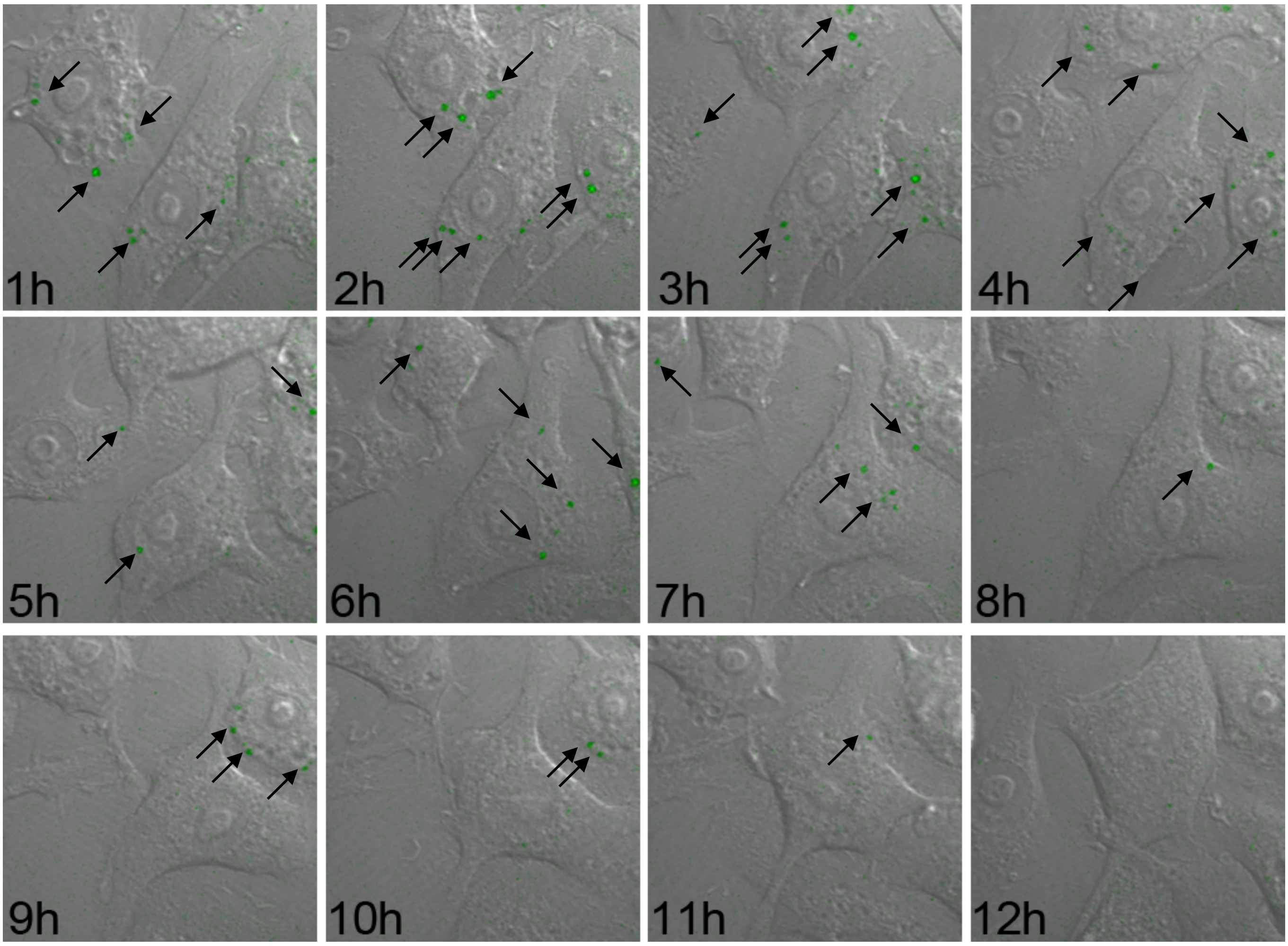

Supplementary Figure 22. Macrophage uptake and degradation of fluorescently-labelled mMPIO-COOH.

Time-course montage of particle degradation by the murine macrophage cell line RAW264.7 obtained by live cell confocal imaging. Particle degradation was imaged over 12 hours, during which time the number of visible particles (indicated by a black arrow) per field of view decreased markedly. Images are representative of the time-point at hourly intervals (**Supplementary Video 1**). See **Supplementary Method 11** for synthesis of fluorescently-labelled mMPIO-COOH.

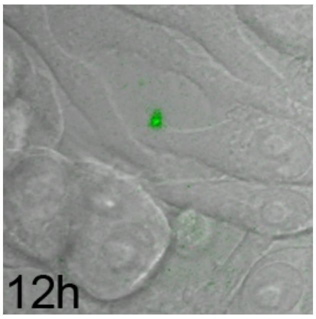

Supplementary Figure 23. Fluorescent mMPIO show excellent photostability during the microscopy experiments.

Particles that were not phagocytosed remained highly fluorescent after 12 hours of imaging. See **Supplementary Method 11** for synthesis of fluorescently-labelled mMPIO-COOH.

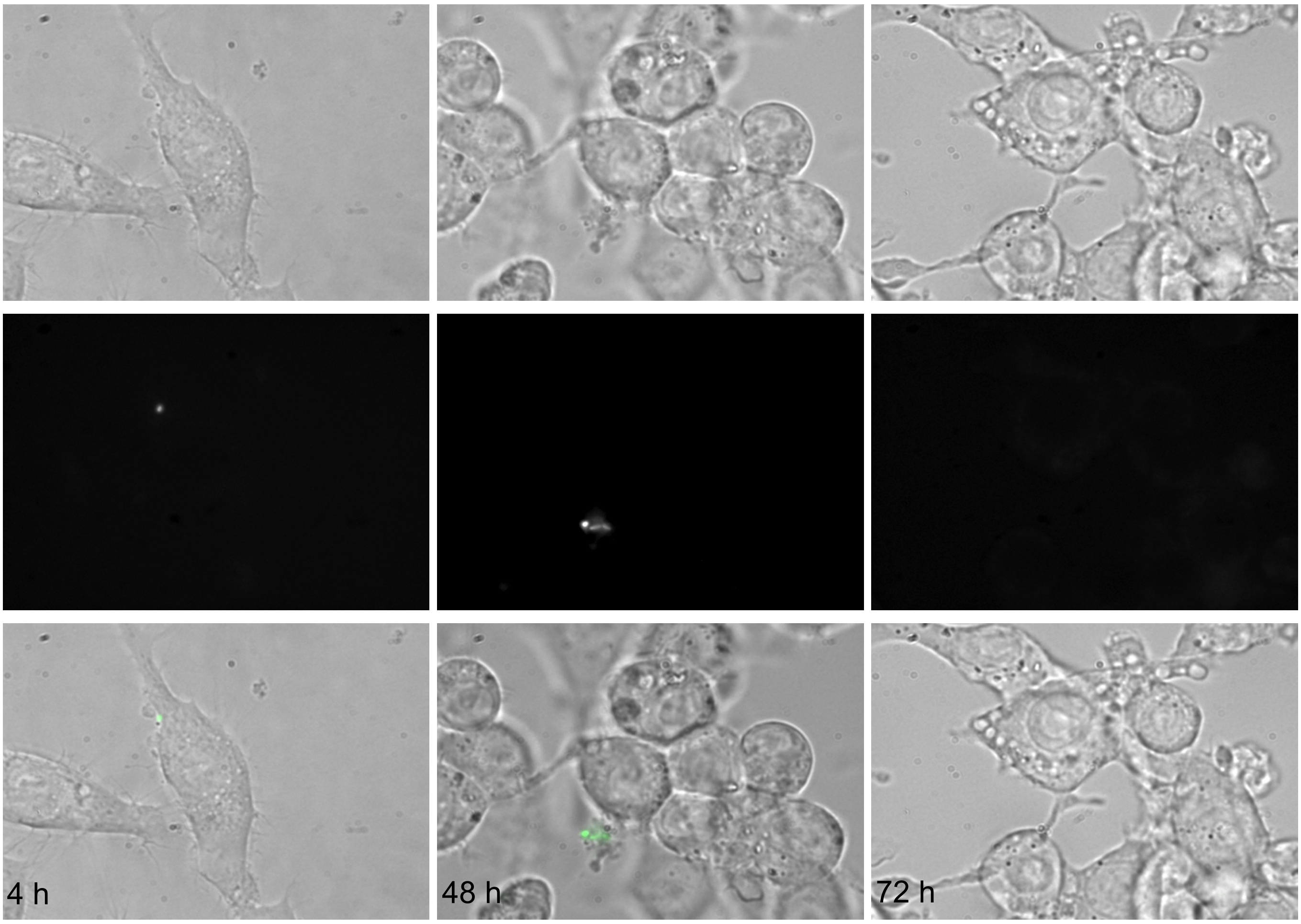

Supplementary Figure 24. Macrophage uptake and degradation of fluorescently-labelled mMPIO-NH_2.

Differential interference contrast (DIC) images (top), single-frame fluorescence images (middle) and overlay of DIC and fluorescence images (bottom) of mMPIO-NH_2 incubated with macrophages at time 4h (left), 48h (centre) and 72h (right). See **Supplementary Method 11** for synthesis of fluorescently-labelled mMPIO-NH_2.

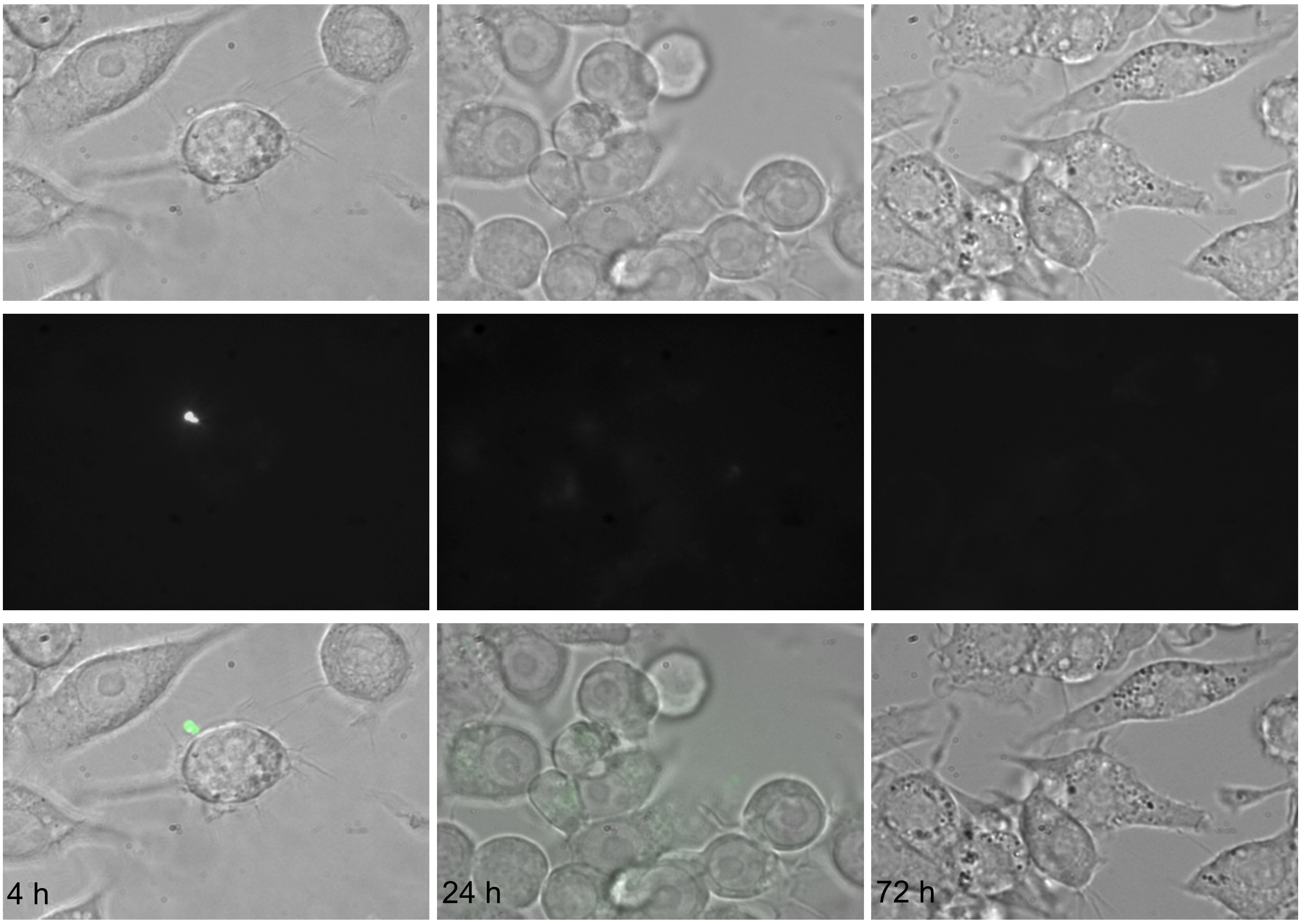

Supplementary Figure 25. Macrophage uptake and degradation of fluorescently-labelled mMPIO-COOH.

Differential interference contrast images (top), single-frame fluorescence images (middle) and overlay of DIC and fluorescence images (bottom) of mMPIO-COOH incubated with macrophages at time 4h (left), 24h (centre) and 72h (right). See **Supplementary Method 11** for synthesis of fluorescently-labelled mMPIO-COOH.

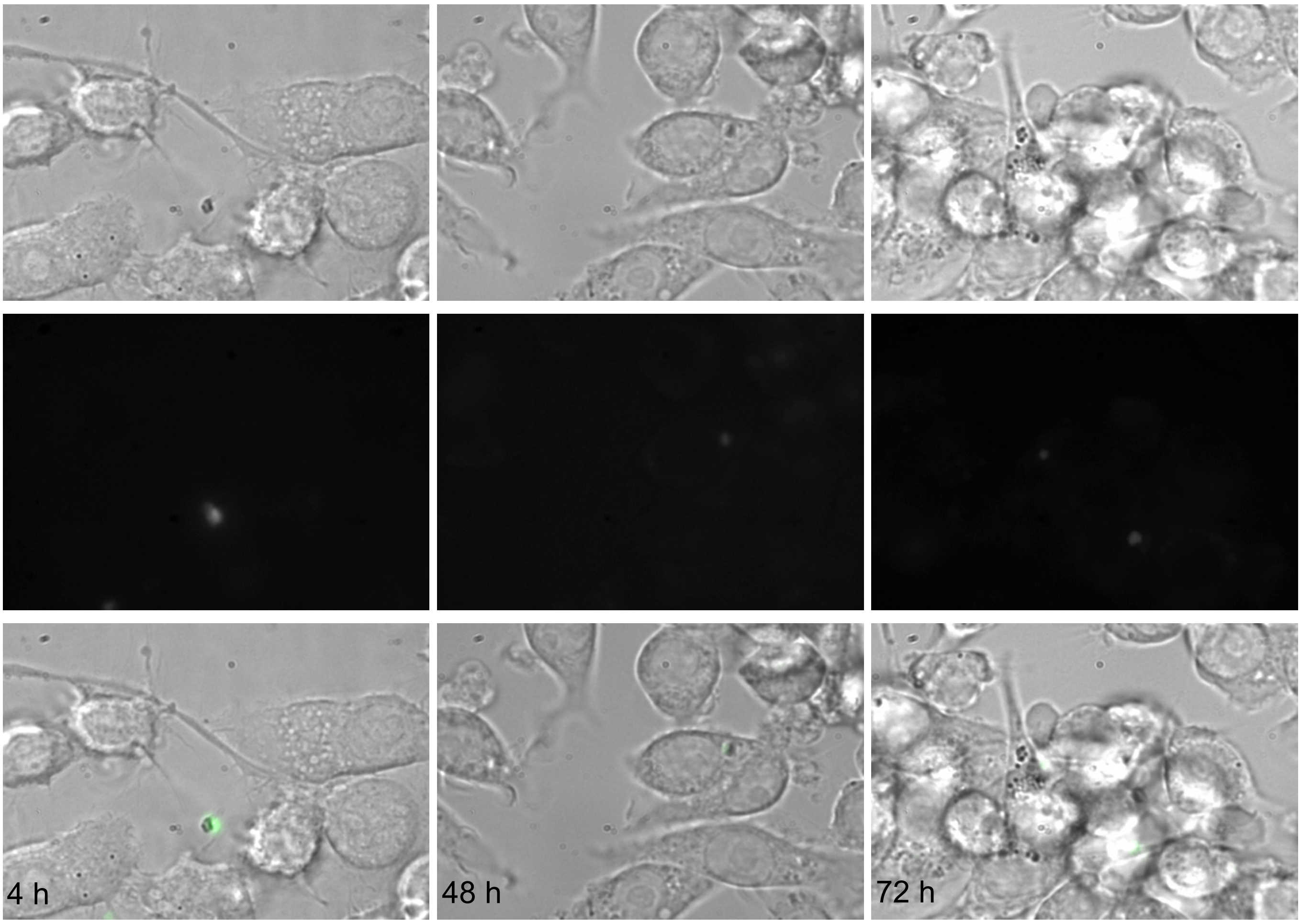

Supplementary Figure 26. Macrophage uptake and degradation of fluorescently labelled MPIO-NH_2.

Differential interference contrast images (top), single-frame fluorescence images (middle) and overlay of DIC and fluorescence images (bottom) of MPIO-NH_2 incubated with macrophages at time 4h (left), 48h (centre) and 72h (right). See **Supplementary Method 12** for synthesis of fluorescently-labelled MPIO-NH_2.

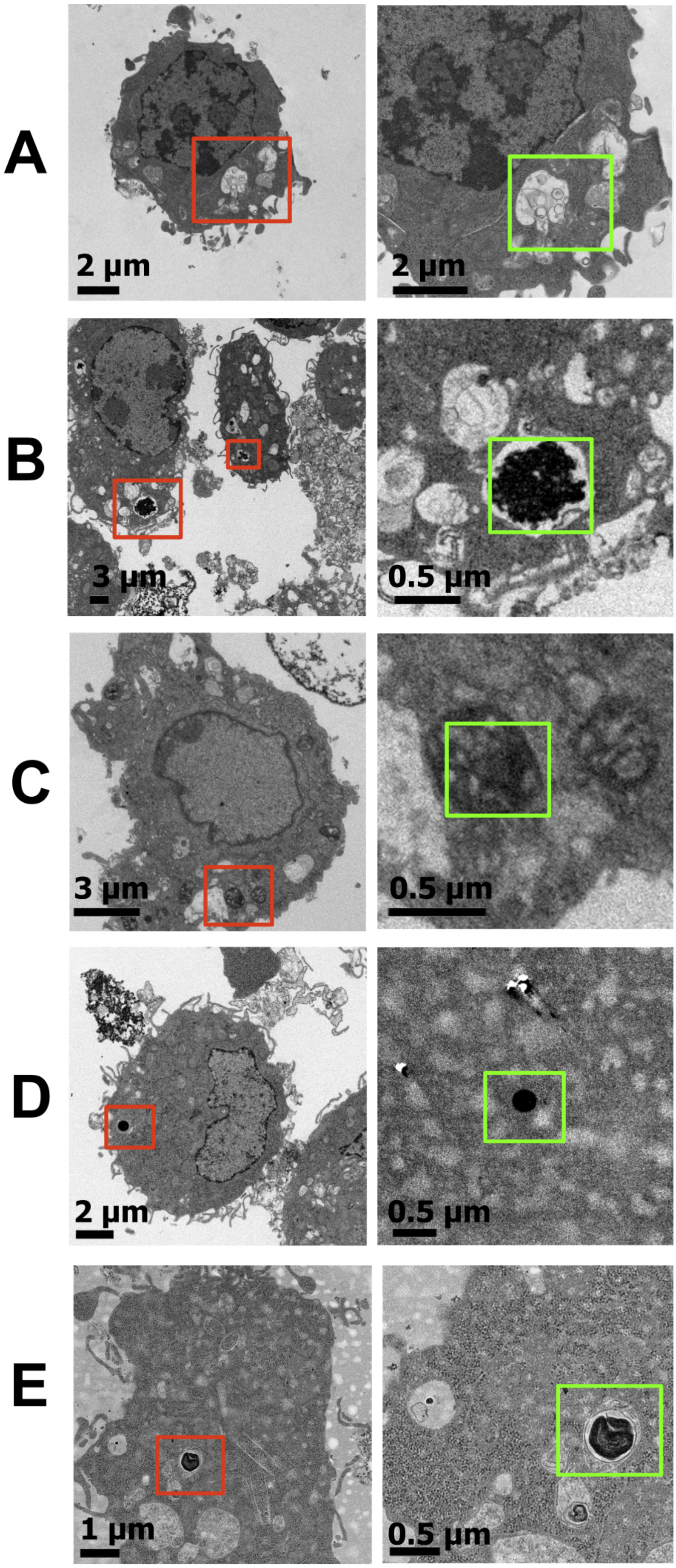
A
2 µm
2 µm
B
3 µm
0.5 µm
C
3 µm
0.5 µm
D
2 µm
0.5 µm
E
1 µm
0.5 µm

Supplementary Figure 27. Intracellular Particle Degradation.

2% UA uranyl acetate stained ultra-thin sections (70-90 nm) as imaged by TEM of RAW cell sections after different incubation times revealed uptake and then loss of mMPIO particles, but negligible degradation of non degradable Dynabead particles. (A) Negative control (RAW cells without mMPIO addition); (B) 1 h after the addition of the mMPIOs; (C) 24 h after the addition of mMPIOs; (D) Dynabeads after 1 h and (E) Dynabeads after 24 h. Left panels are lower magnification EM images. The middle panels show an expansion of the region indicated by the red box.

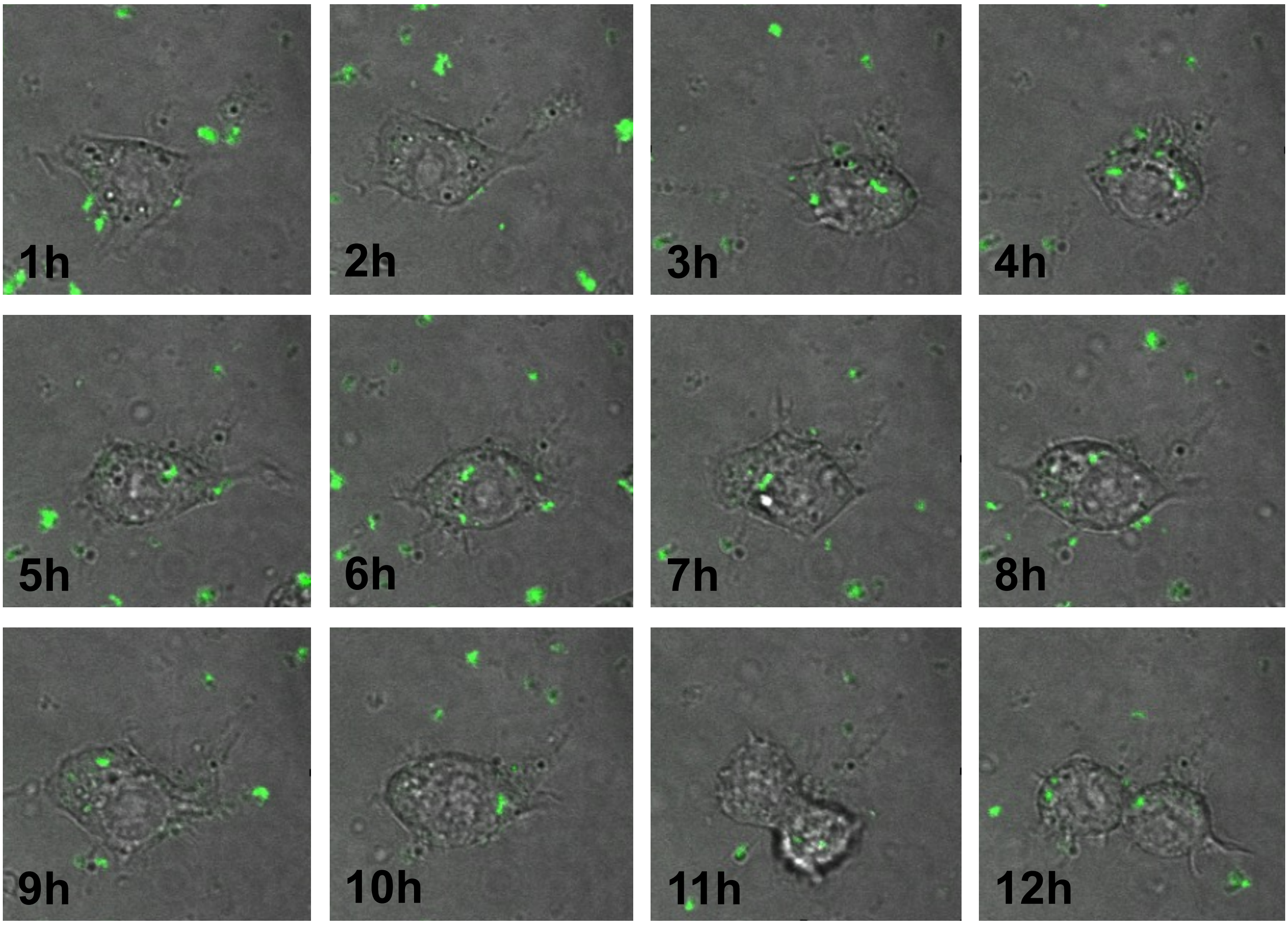

Supplementary Figure 28. A selected example of fluorescently-labelled D-peptide linked mMPIO uptake by a macrophage.

Time-course montage of particle uptake by the murine macrophage cell line RAW264.7 obtained by live cell confocal imaging. Particle uptake was imaged over 12 hours, during which time the number of visible particles within the cell remained stable. The images are a picked example of uptake. The majority of the particles remain outside of the cells. See **Supplementary Figure 29** for a representative image of the time course experiments. *Note:* the particles were shared between the daughter cells during division of the cell tracked (see 10h, 11h and 12h).

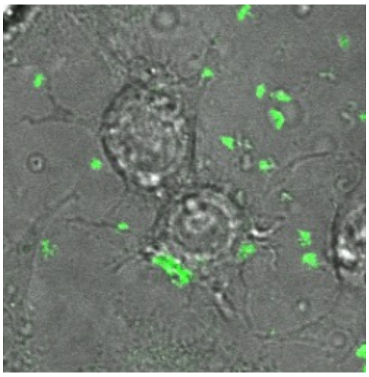

Supplementary Figure 29. A representative example of fluorescently-labelled D-peptide linked mMPIO incubated with macrophages.

The majority of the observed particles were not phagocytosed by the cells during the course of the experiment (13,5 hours), unlike the L-peptide linked mMPIOs (See **Supplementary Figure 22**). The fluorescently-labelled D-peptide linked mMPIOs remained either associated with the cells or passed the cells untouched. See **Supplementary Figure 30** for a quantification of the fluorescent signal of these particles over time.

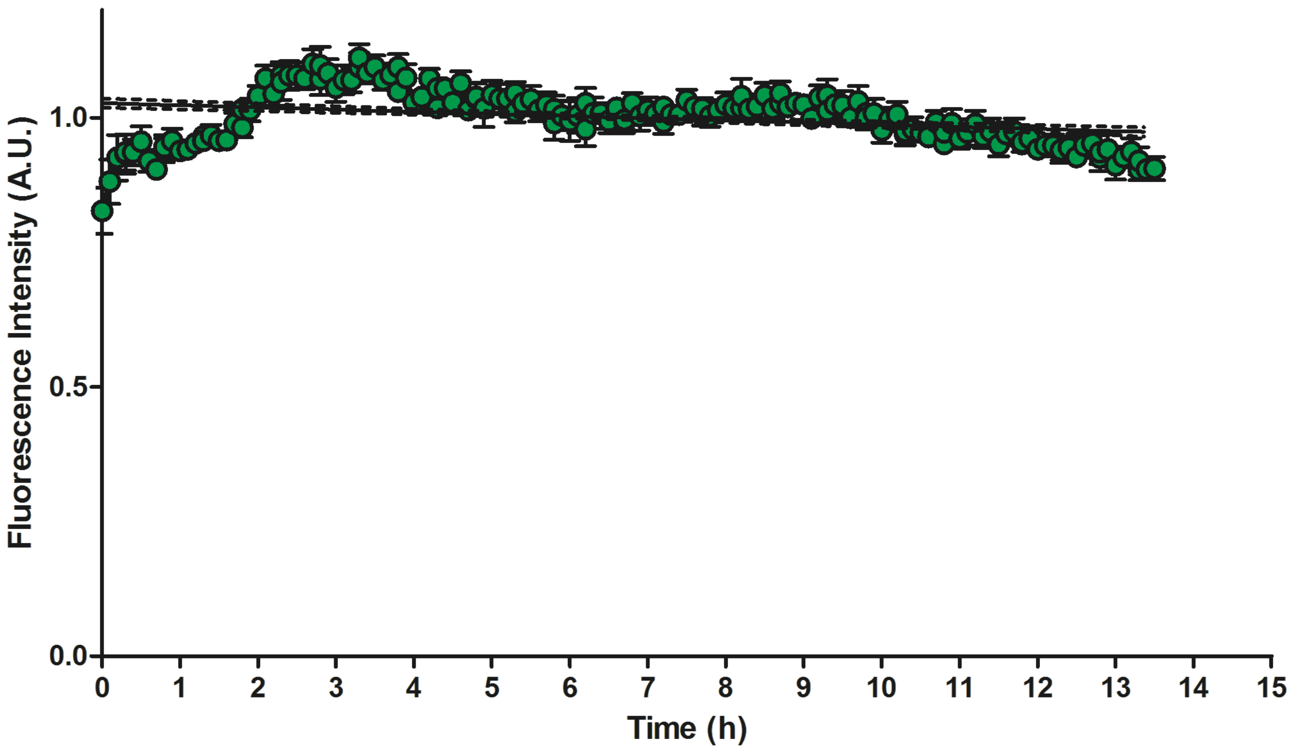

Supplementary Figure 30. Change of fluorescence intensity of the D-mMPIOs incubated with RAW macrophages over time.

Of each frame (5 min, 163 frames in total = 13,5 hour) the fluorescence intensity density was measured for each individual track (n = 7). The values obtained were averaged and the fold change was plotted. There is a minimal decay of the fluorescent signal of the D-peptide linked mMPIOs. Error bars are expressed as mean ± s.d.of the samples. See **Supplementary Figure 31** for a comparison with the L-peptide linked mMPIOs.

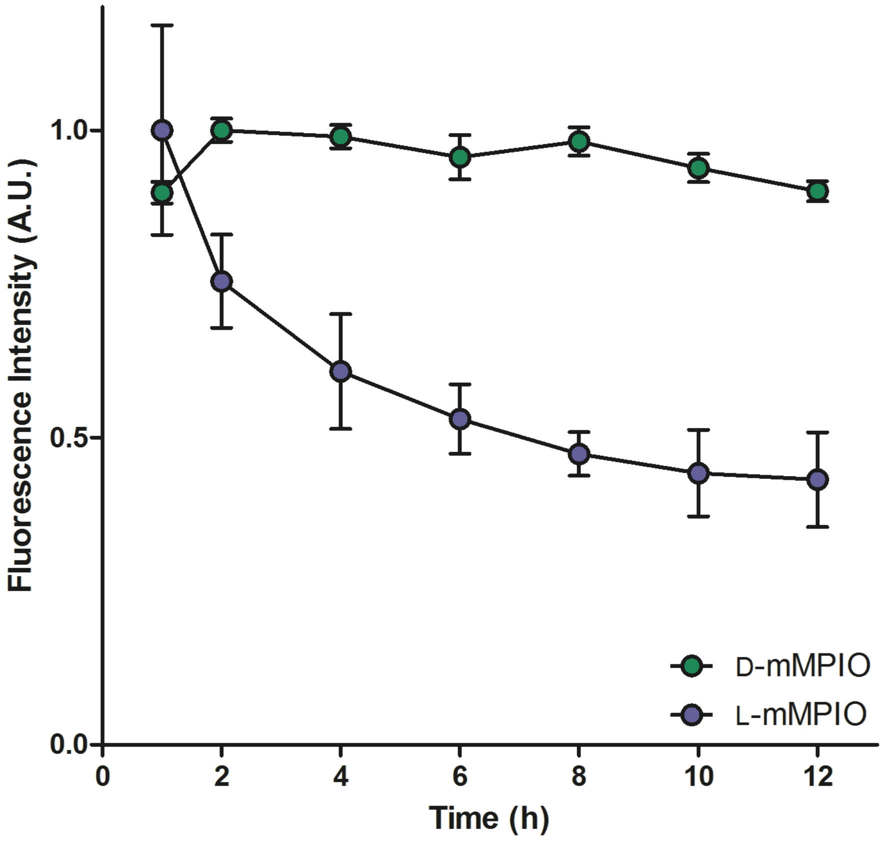

Supplementary Figure 31. Change of fluorescence intensity between L-mMPIOs and D-mMPIOs incubated with RAW macrophages over time.

The fluorescence intensity density was measured for both fluorescently labelled L-mMPIOs (n=5) and D-mMPIOs (n = 7) when incubated with the macrophage cell line RAW264.7. The values obtained were averaged, highest values were set to 1 and the fold change was plotted. There is a minimal decay of the fluorescent signal of the D-peptide linked mMPIOs; however there is a clear decrease of fluorescent signal of the L-peptide linked mMPIOs. Error bars are expressed as mean ± s.d.of the samples.

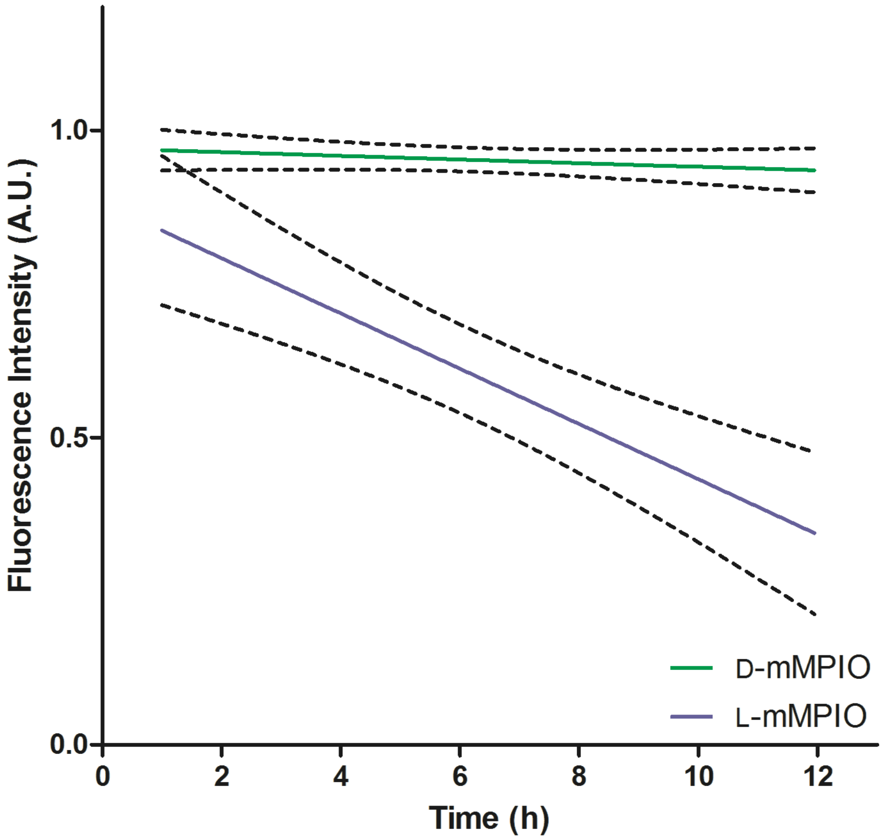

Supplementary Figure 32. Linear regression analysis of the change of fluorescence intensity of L-mMPIOs and D-mMPIOs incubated with RAW macrophages over time.

The r^2 values obtained for the D-peptide linked mMPIOs is 0.0280 and 0.4132 for the L-peptide linked mMPIOs and shows a significant difference between the slopes .

mMPIO biodistribution and toxicological studies

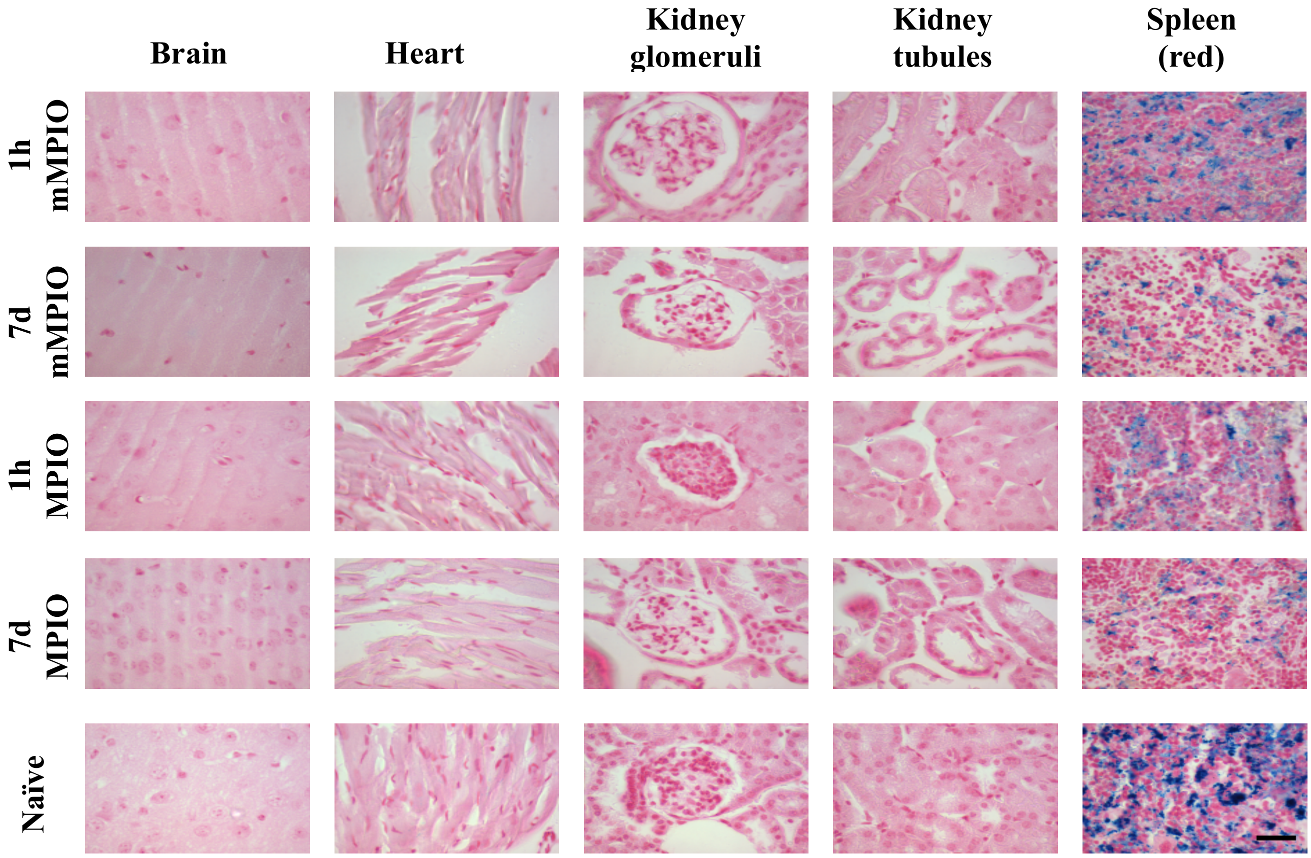

Supplementary Figure 33. mMPIO and MPIO biodistribution.

Representative photomicrographs of Prussian blue-stained tissue sections taken from brain, heart, kidney (glomeruli and tubules) and spleen (red pulp) 1 h and 7 days after intravenous injection of either mMPIO or MPIO. Bottom row shows equivalent sections from a naïve mouse, in which the intrinsic levels of iron present should be noted. Sections have been stained with Prussian blue to identify iron and counterstained with nuclear fast red. Magnification is 400X, scale bar = 50μm.

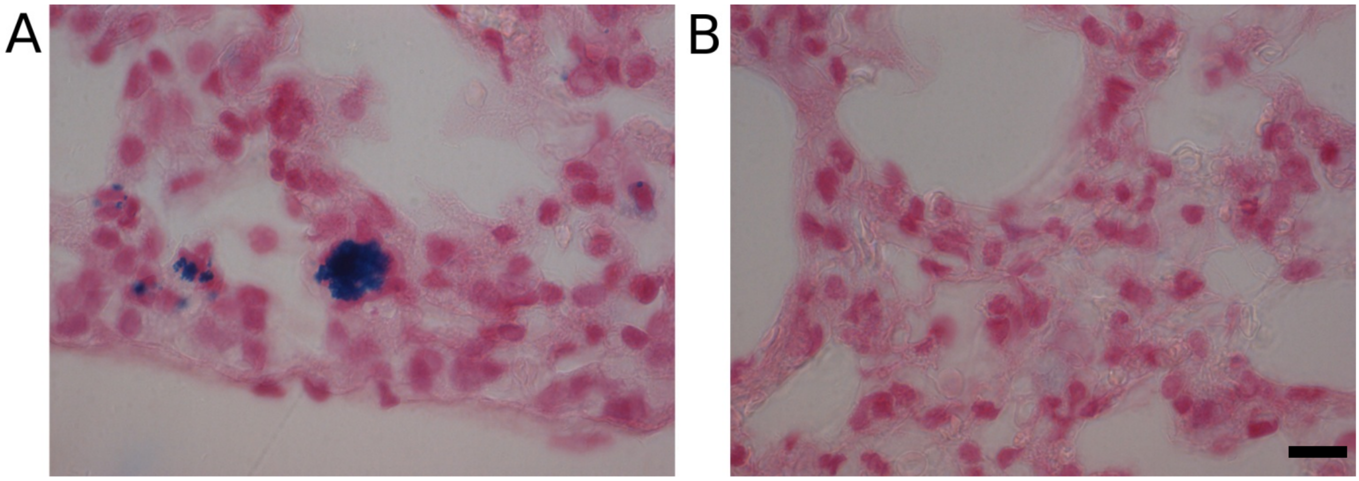

Supplementary Figure 34. Differential behaviour of mMPIO and MPIO in lung.

Representative photomicrographs of Prussian blue-stained tissue sections lungs 7 days after intravenous injection of either MPIO (A) or mMPIO (B). Throughout the time course, from 1 hour to 7 days, the MPIO were found to be sequestered abluminally with the lung. No sequestration was observed following the administration of mMPIO. Sections counterstained with nuclear fast red. Magnification is 400X, scale bar = 50μm.

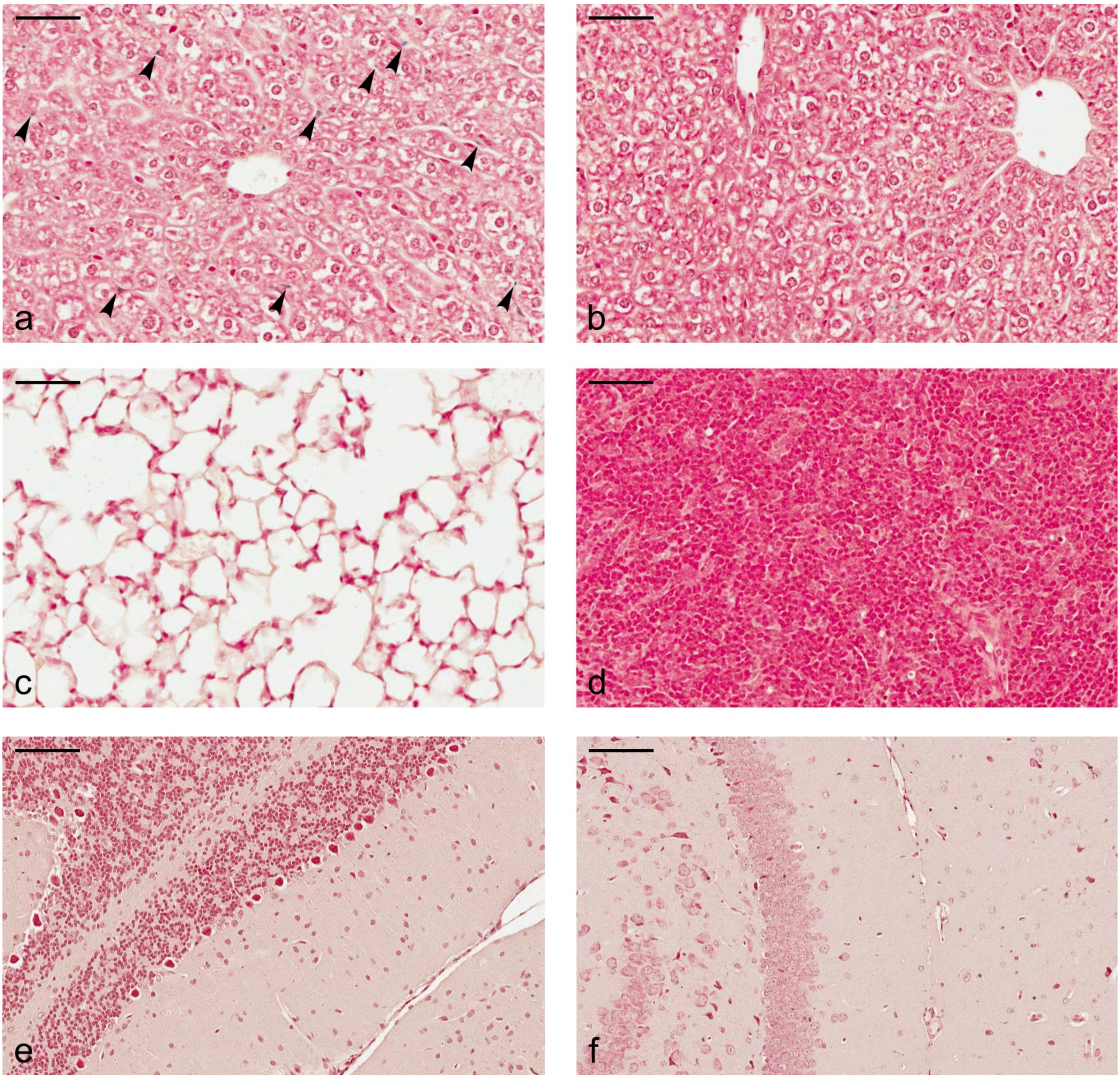

Supplementary Figure 35. Perls´ Prussian blue staining to identify iron deposition after intravenous injection of αhuVCAM-mMPIO.

Representative photomicrographs showing mild, diffuse iron staining (blue) in the liver at 48h (a, black arrows), which was not evident in the liver at 14 days (b). No evidence of iron deposition was found in lung (c), spleen (white pulp) (d), or brain - (e) cerebellum and (f) hippocampus - at 48h after intravenous αhuVCAM-mMPIO injection. Sections were counterstained with Neutral Red. Original magnification is 200X (a-d) and 100X (e-f); scale bar = 50 μm (a-d) or 100 μm (e-f).

Additional experiments with αVCAM1-mMPIO

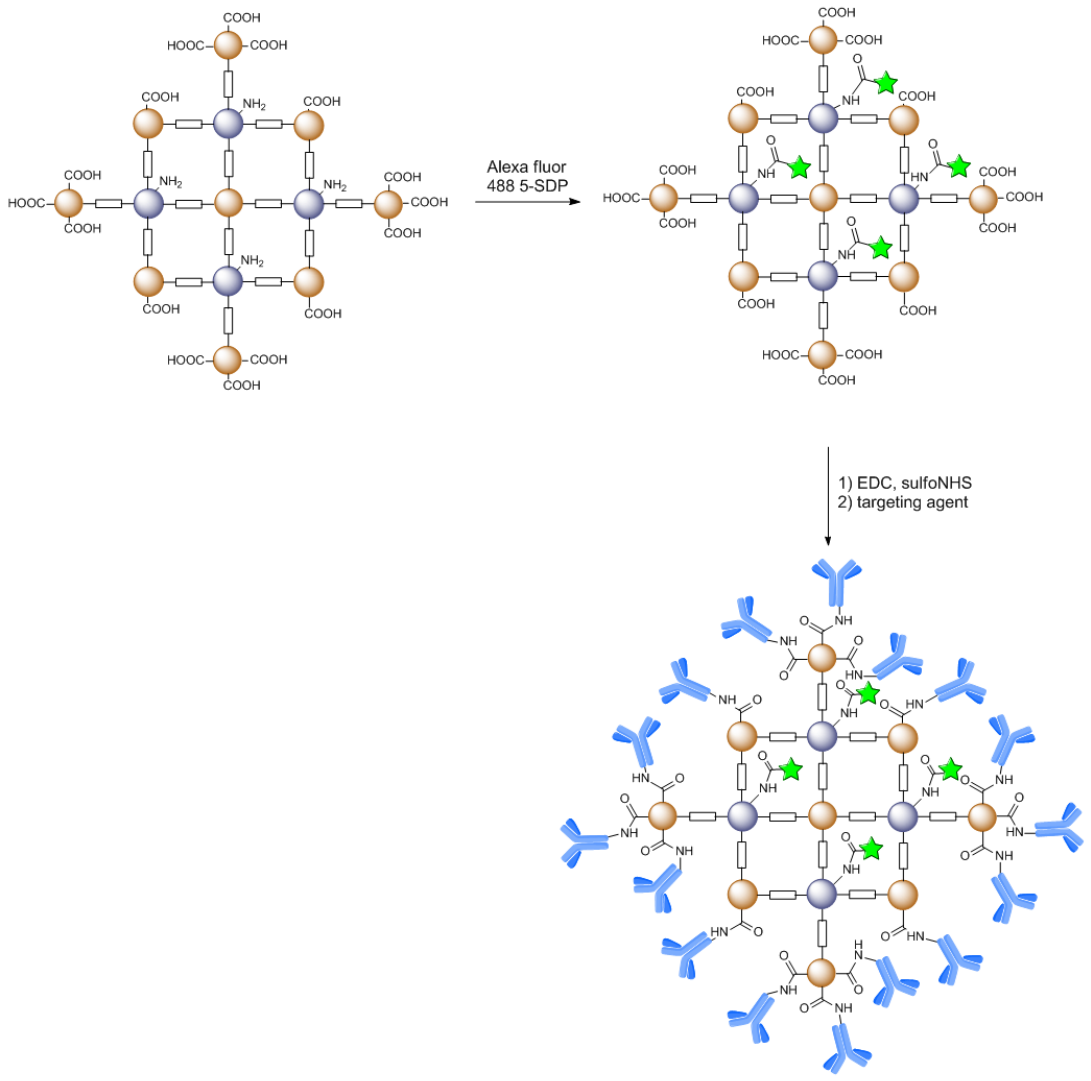

Supplementary Figure 36. Schematic representation of mMPIO-COOH showing the different orthogonal reacting groups present in the particles.

In the interior of the particle primary amino groups allow the conjugation of fluorophores (but potentially other types of small molecule) via several reactions (e.g. amide formation by reaction with activated carboxylic esters or nucleophilic substitutions). The external layer bears an excess of carboxylic acid units that, after being activated by EDC/sulfoNHS, serve to graft multiple copies of the targeting agent (e.g. antibody). The figure represents peptido-NPs as gray spheres, carboxy-NPs as brown spheres, Alexa Fluor 488 as green star and targeting agent in blue, peptide linker is represented as white box between spheres.

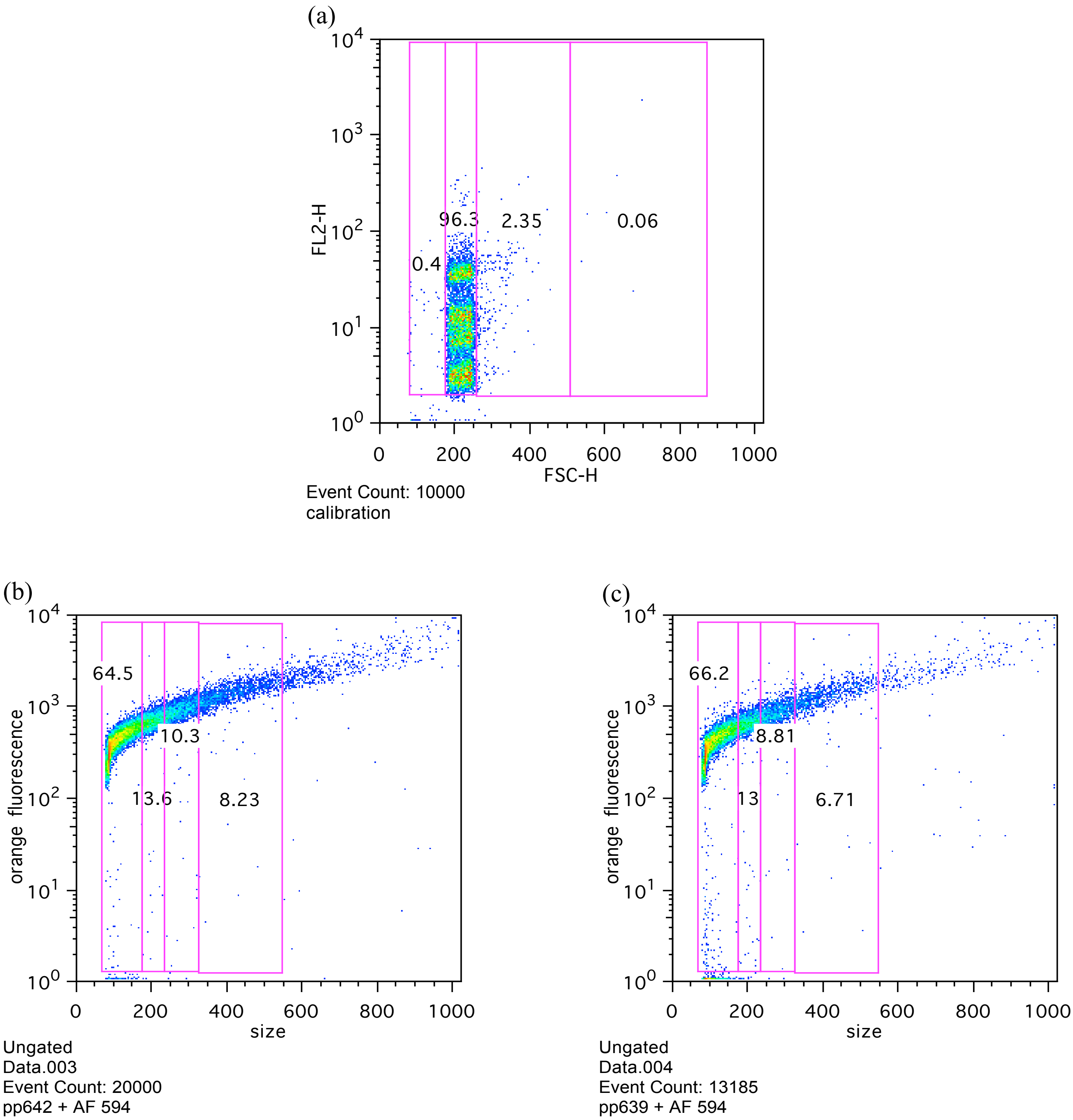

Supplementary Figure 37. Determination of antibody density in αVCAM-AF488-mMPIO and IgG-AF488-mMPIO.

FACS analysis of particles bound to secondary goat anti-mouse Alexa Fluor 594 antibody. (a) Dako Qifikit Calibration beads. 5 populations of 10 μm beads with mouse IgG at different densities; 6, 38, 169, 590 and 1690 molecules per μm^2. Particles incubated with rabbit anti-mouse Alexa Fluor 594 at saturating concentration. (b) αVCAM-AF488-mMPIO incubated with rabbit anti-rat Alexa Fluor 594 at

saturating concentration. (c) IgG-AF488-mMPIO incubated with rabbit anti-rat Alexa Fluor 594. The orange fluorescence intensity obtained with the VCAM-1- and IgG- particles both exceeded the fluorescence signal obtained from the highest density calibration bead, indicating that the density of antibody on the surface of the respective particles was greater than 1690 molecules per μm^2. Both rabbit anti-mouse antibody and rabbit anti-rat antibody were labelled with 4 mol of Alexa Fluor 594 per mol of protein. Static binding experiments (**Supplementary Fig. 38**) and in vivo experiments (**Fig. 5 and Supplementary Fig. 39**) suggest that the high antibody density does not affect antibody binding properties.

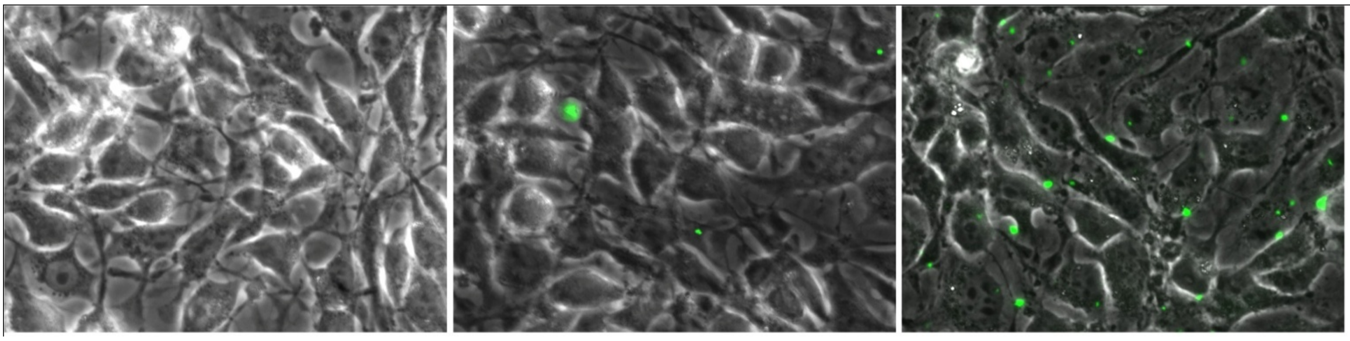

Supplementary Figure 38. αVCAM-AF488-mMPIO binding to activated endothelial cells.

PBS (left), control IgG-AF488-mMPIO (middle) and αVCAM-AF488-mMPIO (right) were added to plates containing activated murine endothelial (sEnd) cells and incubated for 30 minutes. αVCAM-AF488-mMPIO showed high binding affinity to the activated endothelial cells, whilst IgG-AF488-mMPIO showed low non-specific binding to the endothelial cells.

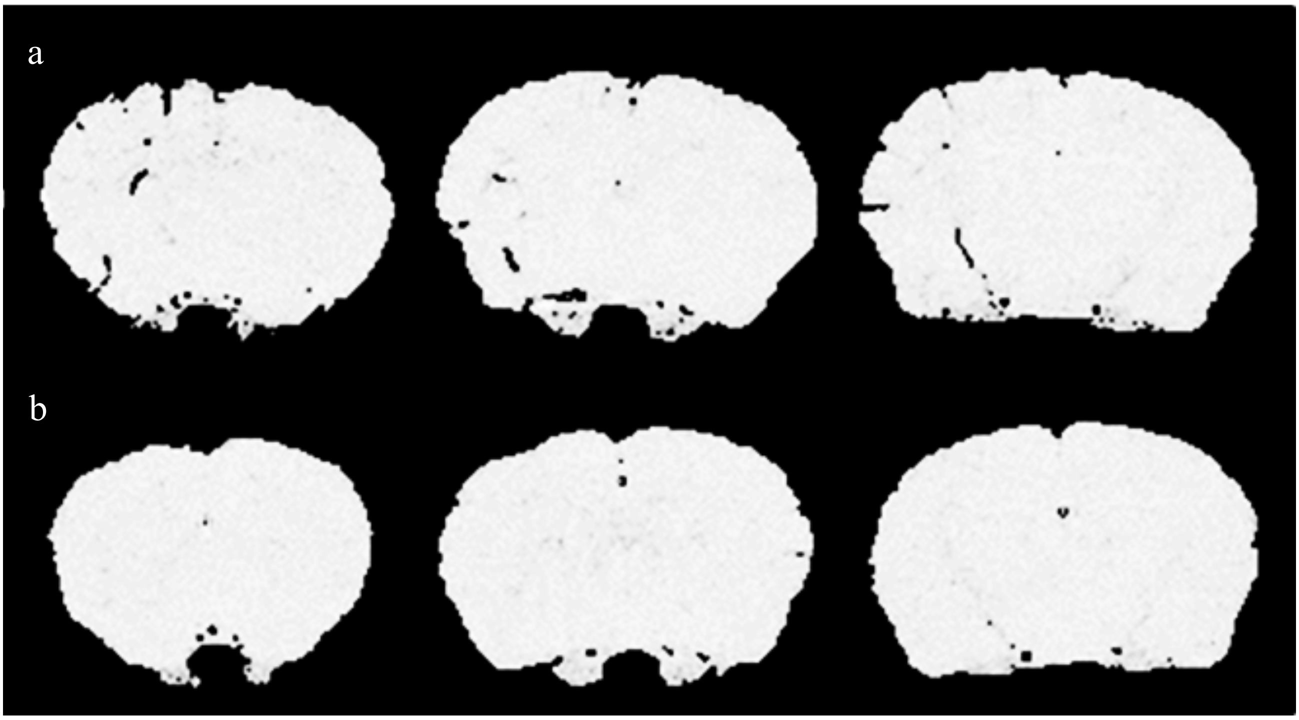

Supplementary Figure 39. In vivo VCAM1 targeted experiments.

Each T_2^*-weighted data set was converted into tiff images, manually masked to exclude extracerebral structures and converted to 8-bit grey-scale. The images were thresholded at a consistent level in the grey channel, such that any pixels of signal intensity > 3 S.D. below the mean intensity of normal brain were set to zero (black) and all others were set to 1 (white). **(a)** Three masked and thresholded T_2*-weighted images from a 3D dataset obtained from a mouse injected intrastriatally with 20 ng of IL-1β in 1μl of saline 3 h before intravenous injection of αVCAM-mMPIO (4mg Fe / kg body weight). Focal hypointense areas (black) in the left hemisphere reflect the specific αVCAM-mMPIO retention on acutely activated vascular endothelium with negligible contrast effects in the contralateral control hemisphere. **(b)** Three masked and thresholded T_2*-weighted images from a 3D dataset obtained from a mouse injected intrastriatally with 20 ng of IL-1β in 1μl of saline 3 h before intravenous injection of the control isotype IgG-mMPIO (4 mg Fe / kg body weight). Negligible contrast effects arising from the mMPIO are evident. The masked and thresholded images were analysed in ImagePro (Media Cybernetic, UK). The same images as shown in Figure 5 (a,b) are shown here.

Characterization of Peptides

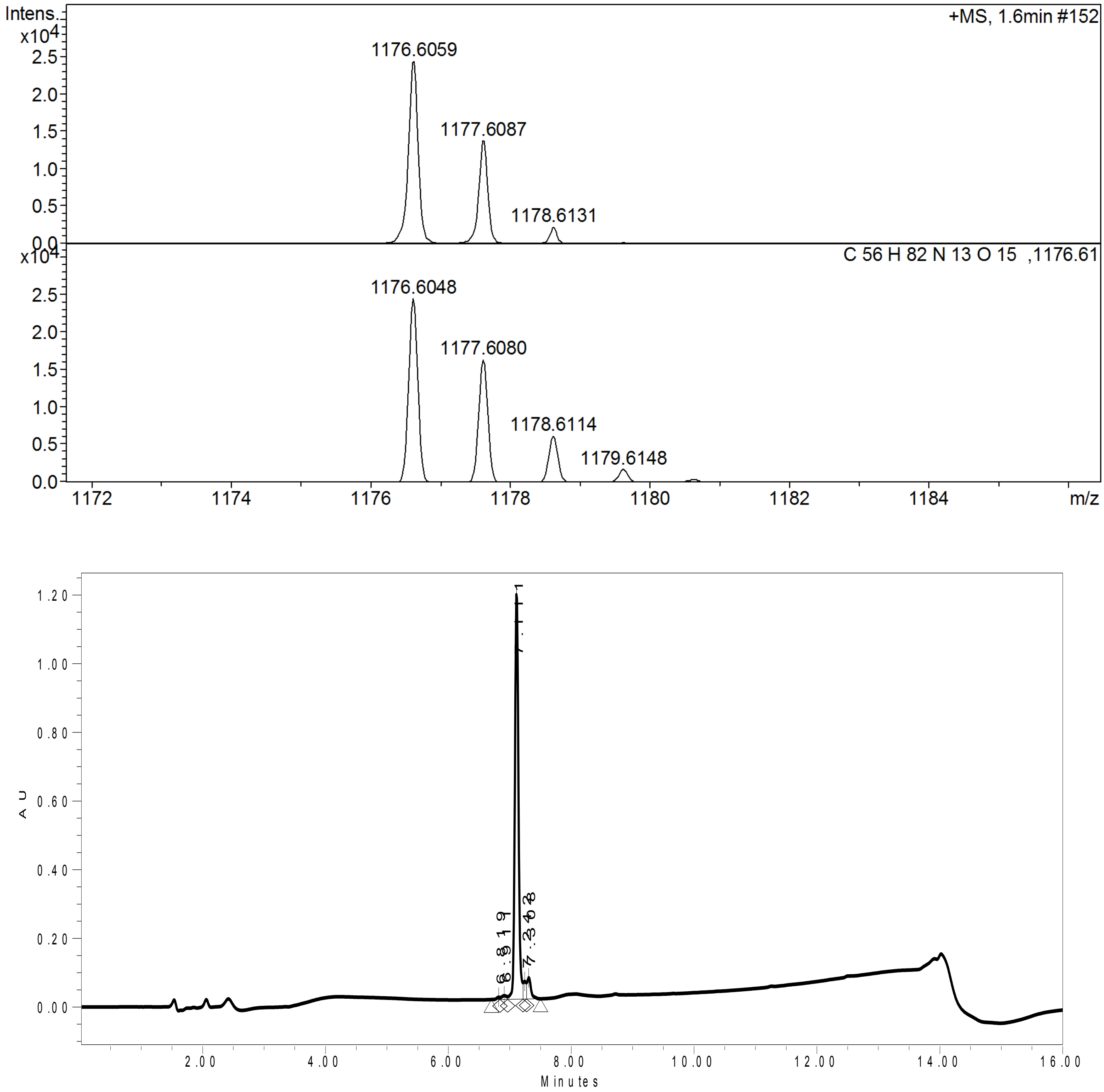

Supplementary Figure 40. Characterization of peptide 1.

High resolution mass spectra (ESI+) (top), theoretical isotopic distribution mass modelling (middle) and HPLC trace at 214 nm (bottom) of peptide **1**.

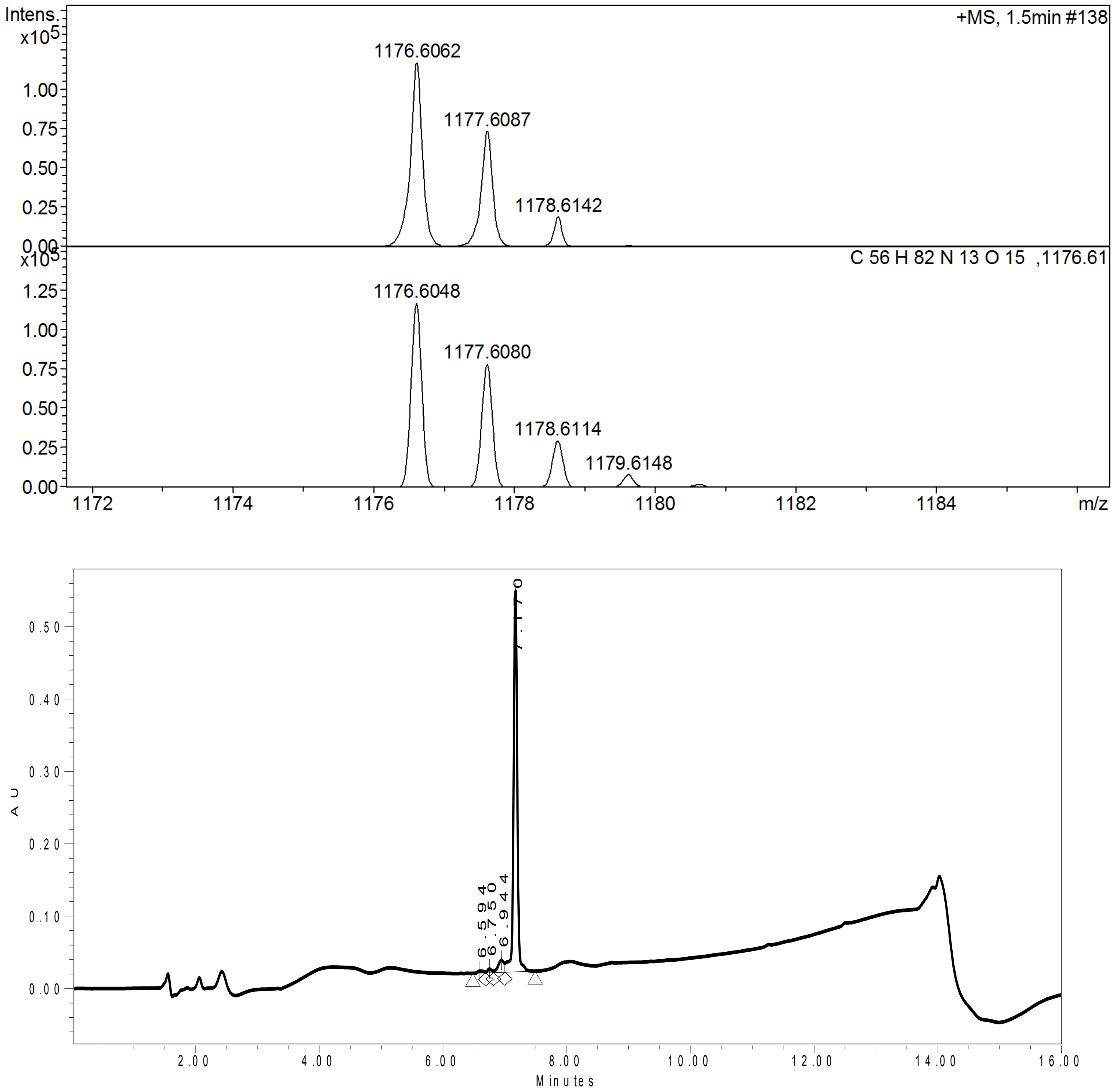

Supplementary Figure 41. Characterization of peptide 2.

High resolution mass spectra (ESI+) (top), theoretical isotopic distribution mass modelling (middle) and HPLC trace at 214 nm (bottom) of peptide **2**.

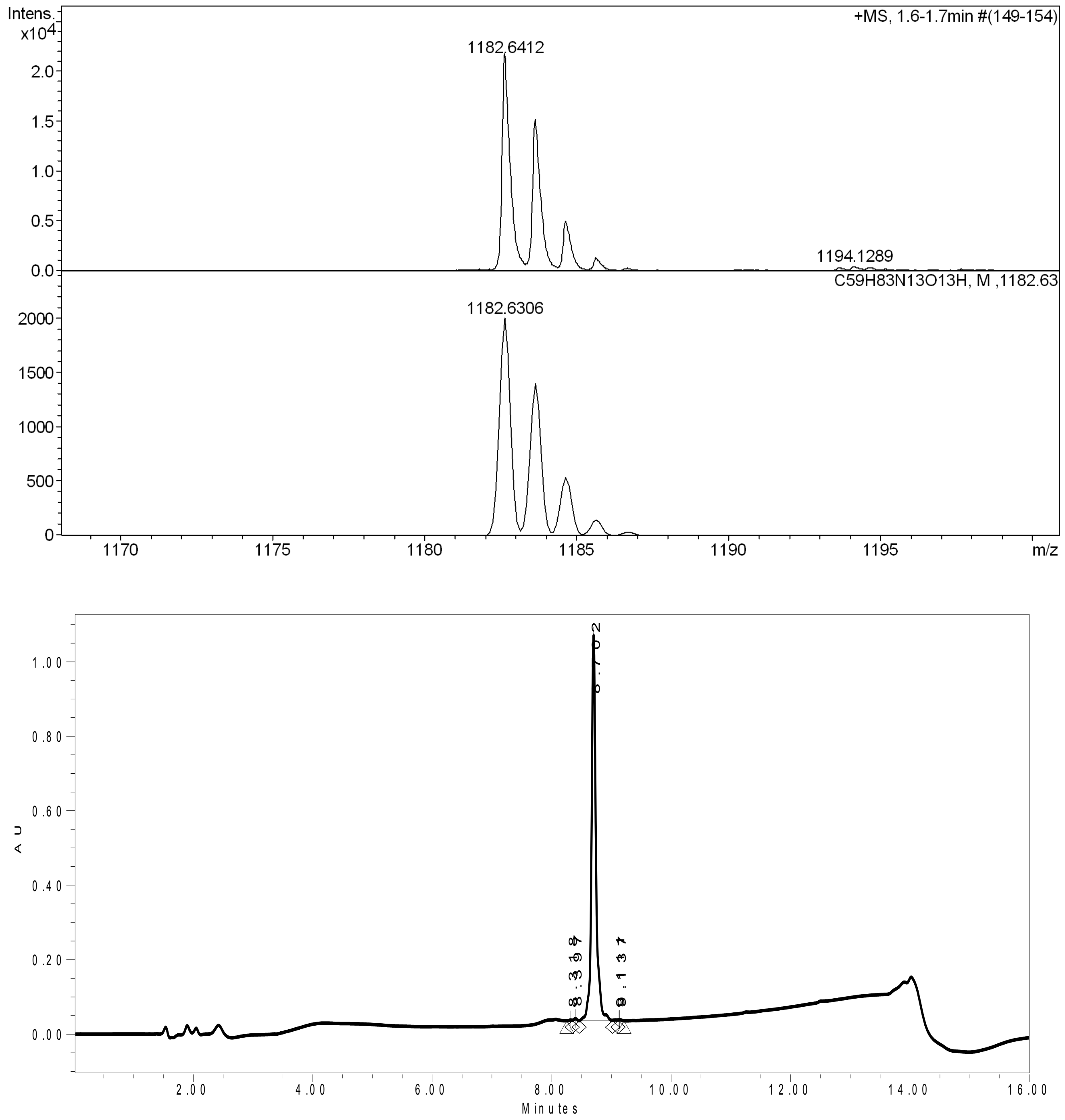

Supplementary Figure 42. Characterization of peptide 3.

High resolution mass spectra (ESI+) (top), theoretical isotopic distribution mass modelling (middle) and HPLC trace at 214 nm (bottom) of peptide **3**.

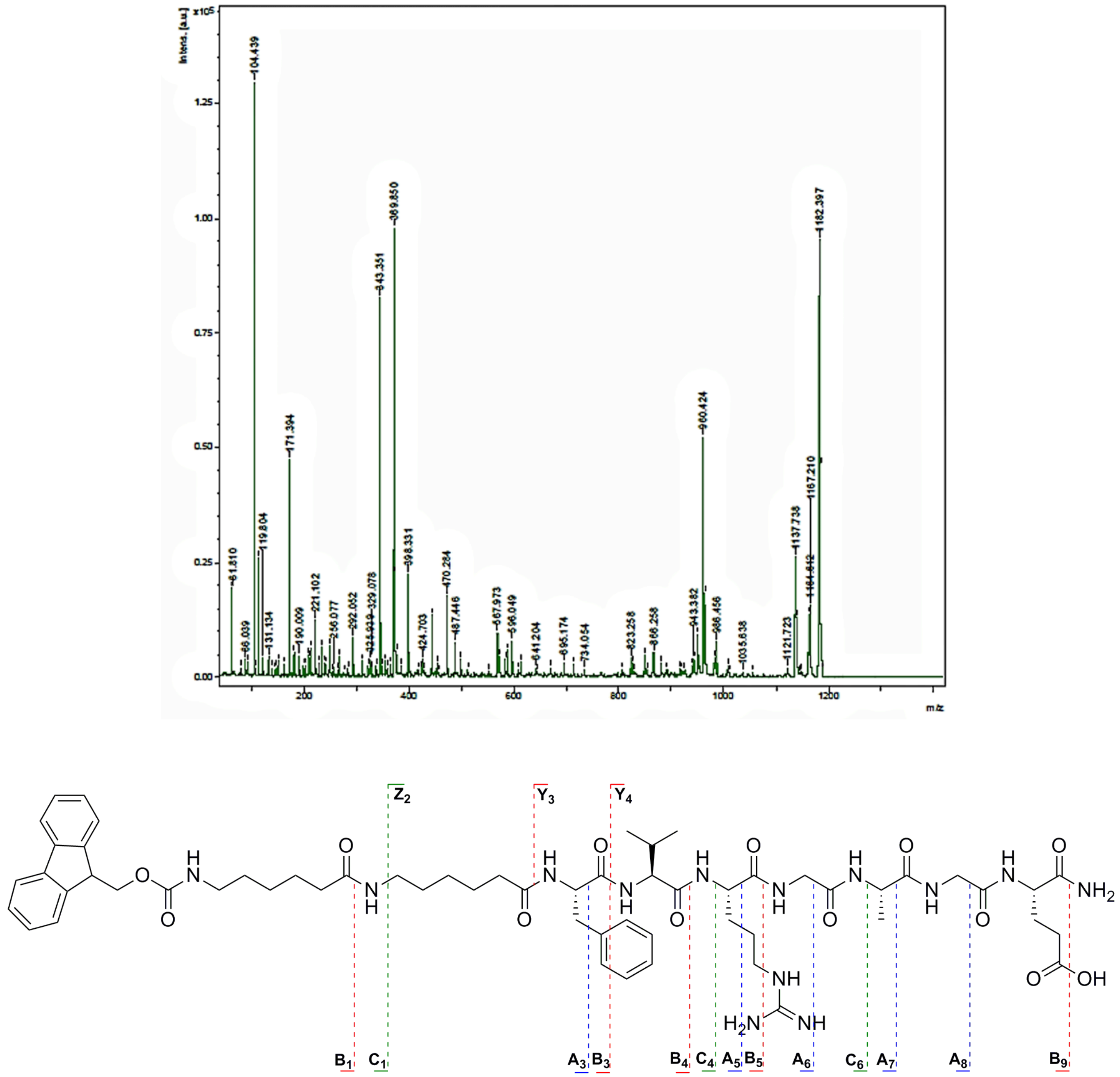

Supplementary Figure 43. Characterization of peptide 3.

MS/MS spectrum of peptide **3** and observed fragmentation pattern.

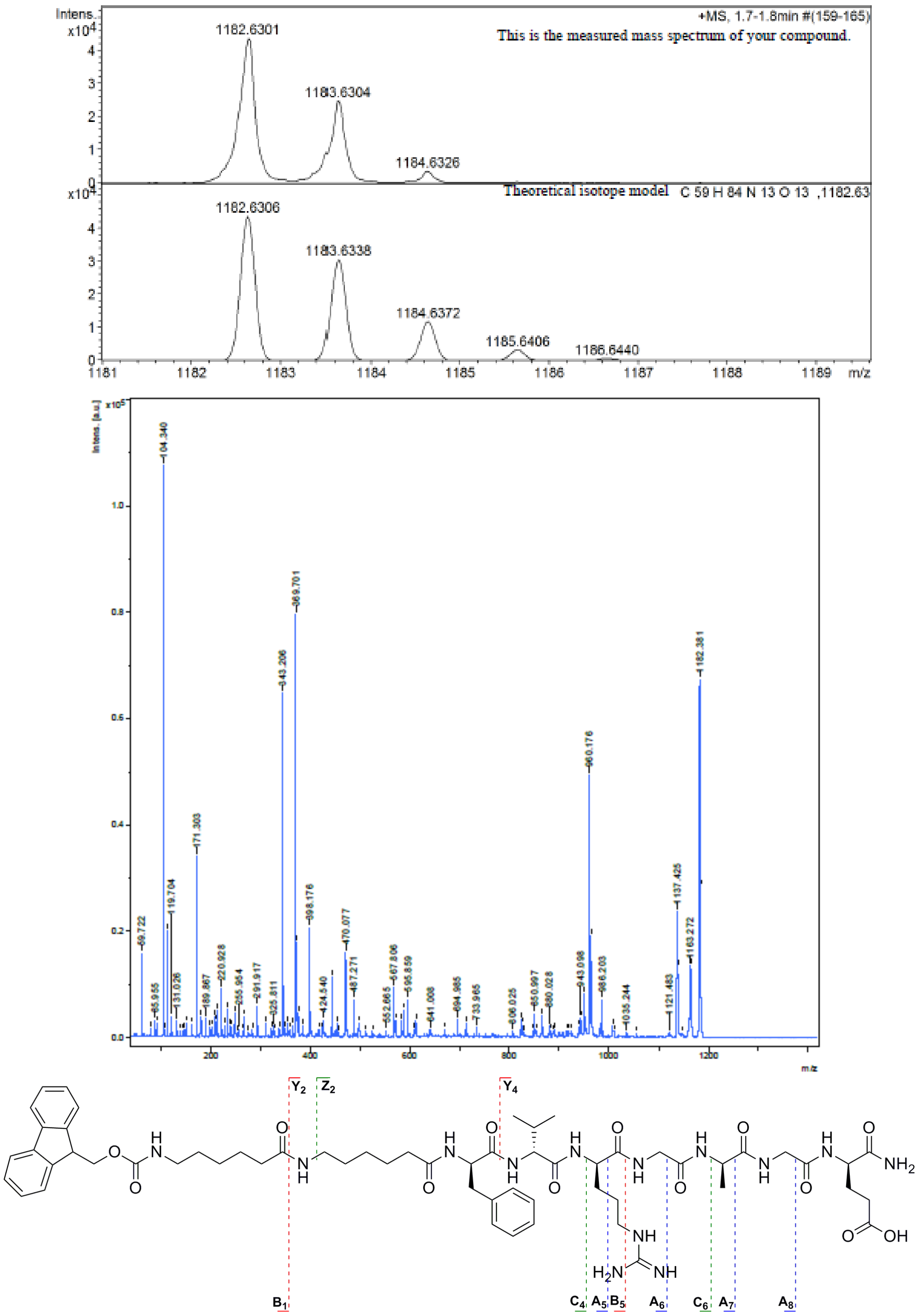

Supplementary Figure 44. Characterization of peptide 4.

High resolution mass spectra (ESI+) and theoretical isotopic distribution mass modeling (top) MS/MS spectrum of peptide **4** (middle) and observed fragmentation pattern (bottom).

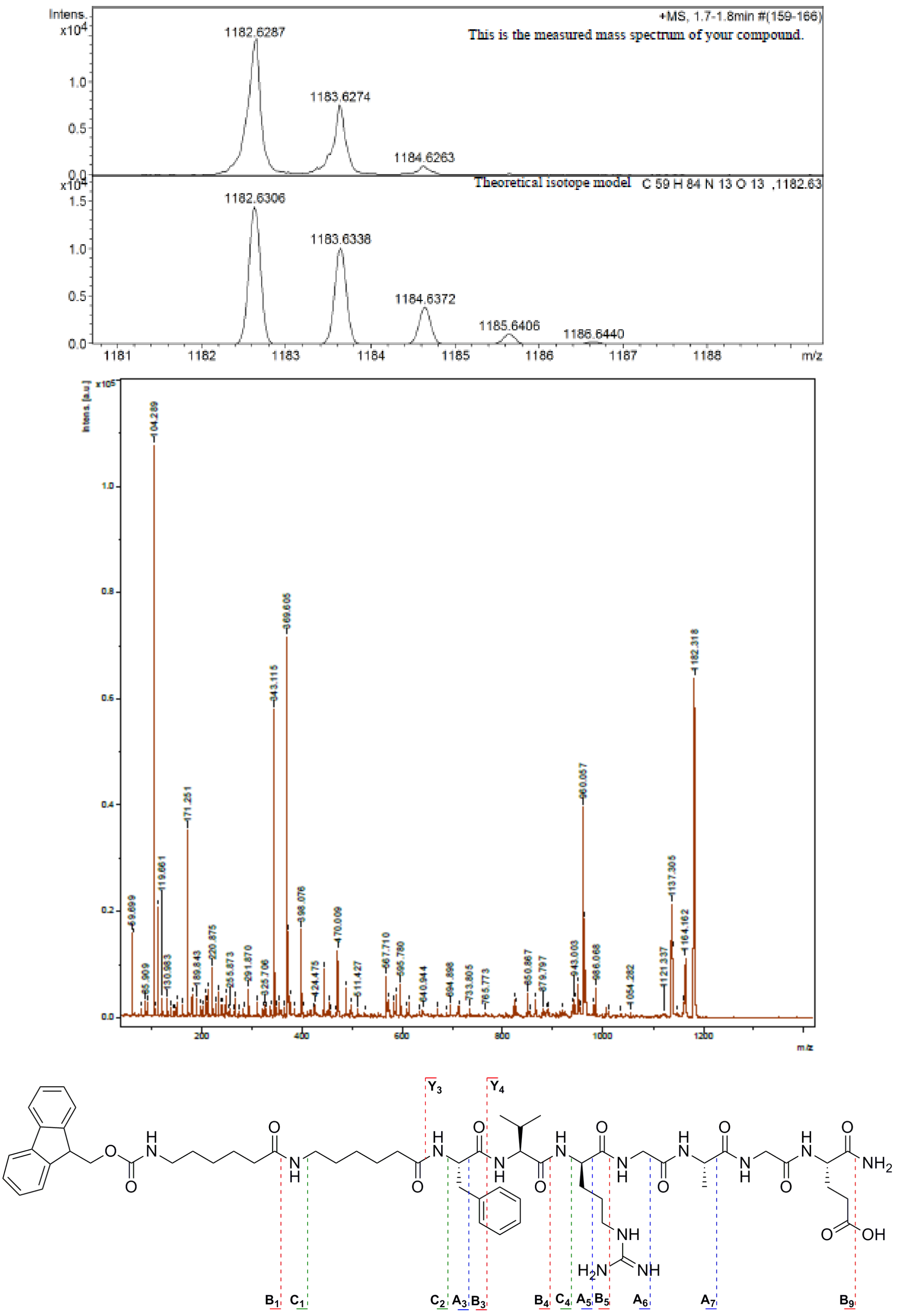

Supplementary Figure 45. Characterization of peptide 5.

High resolution mass spectra (ESI+) and theoretical isotopic distribution mass modeling (top) MS/MS spectrum of peptide **5** (middle) and observed fragmentation pattern (bottom).

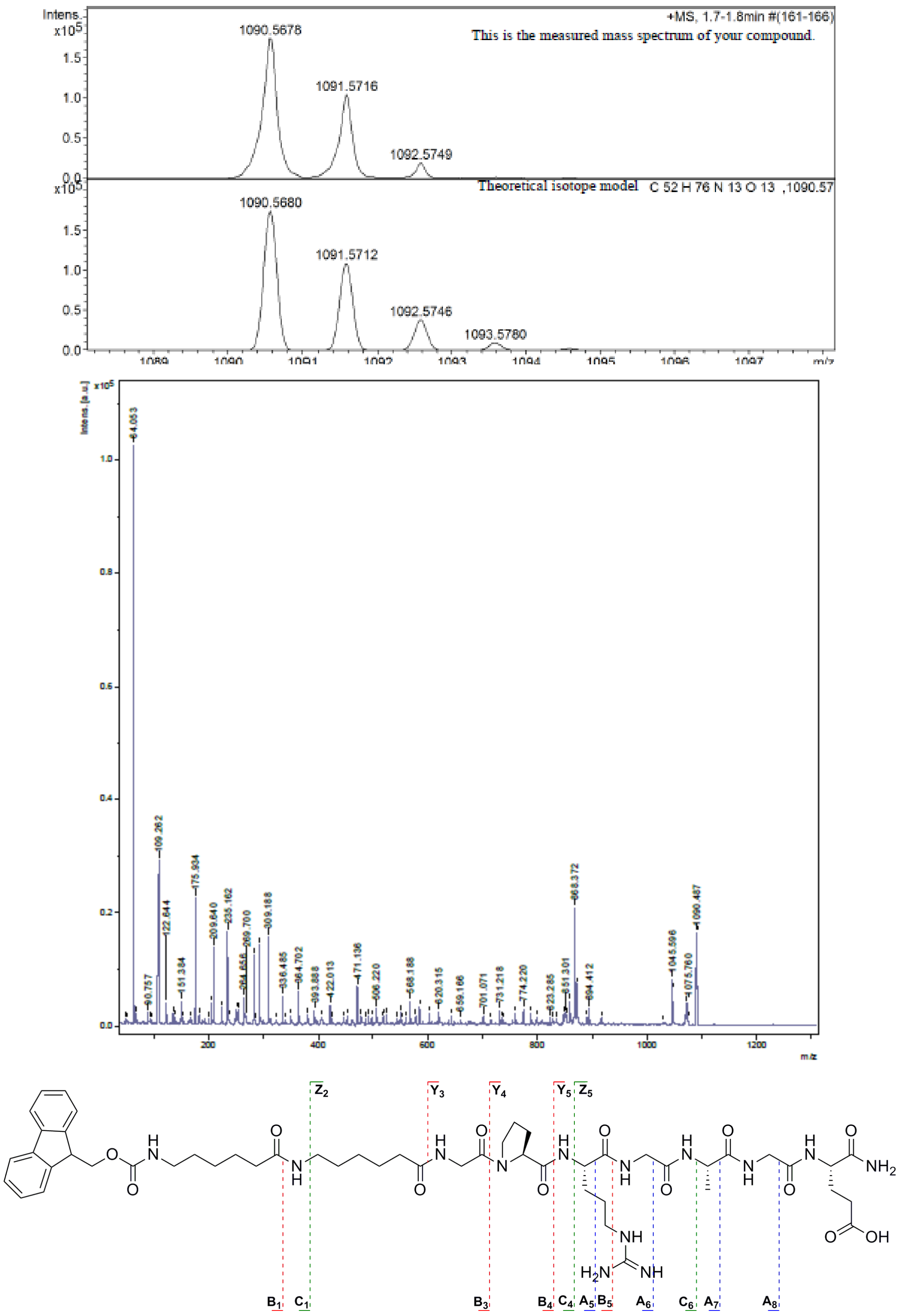

Supplementary Figure 46. Characterization of peptide 6.

High resolution mass spectra (ESI+) and theoretical isotopic distribution mass modeling (top) MS/MS spectrum of peptide **6** (middle) and observed fragmentation pattern (bottom).

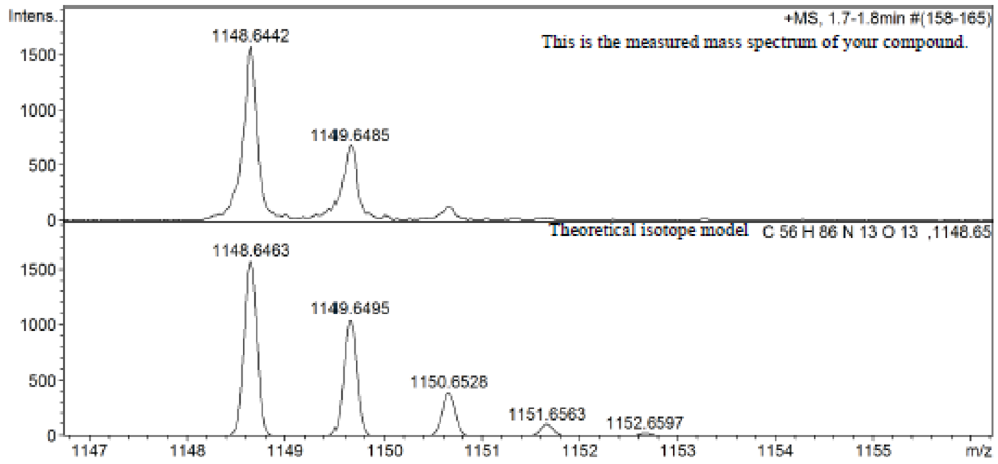

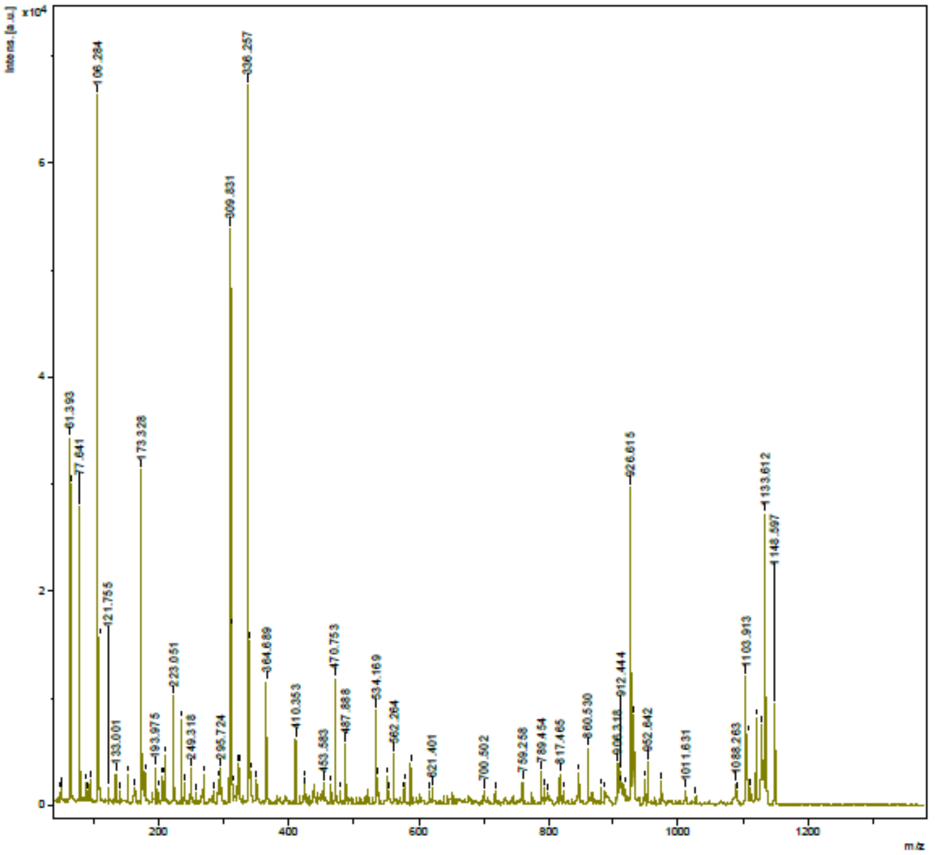

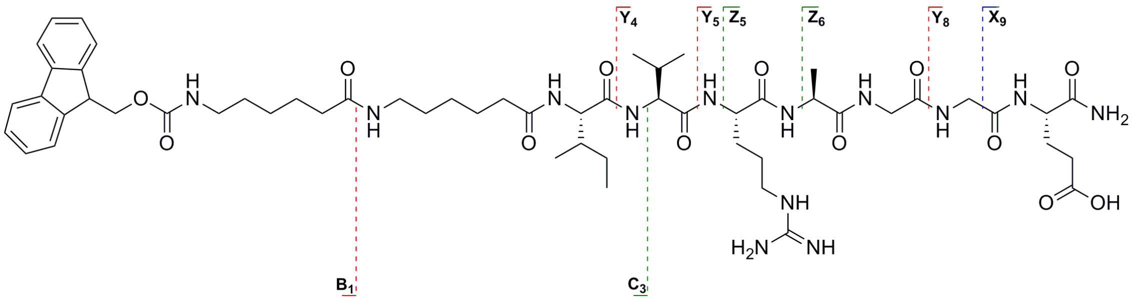

Supplementary Figure 47. Characterization of peptide 7.

High resolution mass spectra (ESI+) and theoretical isotopic distribution mass modeling (top) MS/MS spectrum of peptide **7** (middle) and observed fragmentation pattern (bottom).

Supplementary Tables

Particle synthesis

Product	Dextran (g)	Dextran Lot Nr. [a]	Z_{ave} (nm)	% Fe	% C	% H
**a**	12.0	1331472	28.6	13.1	33.2	4.82
**b**	12.0	1331472	27.7	11.7	33.9	5.62
**c**	12.0	1331472	29.6	11.2	34.6	5.87
**d**	10.0	1331472	52.3	14.2	32.1	4.84
**e**	10.0	1382459	40.9	11.9	34.2	5.00
**f**	10.0	1382459	44.2	9.4	35.8	4.41
**g**	10.0	1382459	44.2	11.6	34.7	5.88
**h**	9.0	1382459	64.9	21.4	32.0	4.89
**i**	9.0	1382459	64.9	21.7	32.0	4.83
**j**	9.0	1382459	60.6	24.1	25.8	3.11
**k**	7.3	HX4271	56.4	19.8	28.7	4.57
**l**	7.1	HX4271	52.6	4.16	36.65	5.77

Supplementary Table 1. Optimization of particle size for the synthesis of dextran covered iron oxide nanoparticles.

[a]Lot numbers 1331472 and 1381472 correspond to dextran from Leuconostoc mesenteroides average molecular weight 9000-11000 from Sigma-Aldrich (cat. Nr. D9260), lot number HX4271 corresponds to Dextran 10 Pharmaceutical Quality from Pharmacosmos, Denmark. The size and composition of the dextran covered iron oxide nanoparticles is highly dependent on the dextran lot employed.

NPs	Dextran Lot Nr. [a]	% Fe	% C	% H	% N	N (μmol/mg Fe)[b]
**h**	1382459	12.5	36.11	5.71	1.11	6.31
**i**	1382459	11.6	36.83	3.94	0.83	5.10
**j**	1382459	18.2	31.30	3.54	0.94	3.88
**k**	HX4271	16.1	35.69	5.57	2.56	11.3
**l**	HX4271	13.1	36.65	6.46	5.47	29.8

Supplementary Table 2. Composition of different amino terminated iron oxide nanoparticles. Dextran coated iron oxide nanoparticle's composition exerts an important influence during crosslinking step. Higher Fe content usually yields lower amino loadings that further influence in particle reactivity towards mMPIO synthesis. [a]Lot number 1381472 correspond to dextran from Leuconostoc mesenteroides average molecular weight 9000-11000 from Sigma-Aldrich (cat. Nr. D9260), lot number HX4271 corresponds to Dextran 10 Pharmaceutical Quality from Pharmacosmos, Denmark. [b] Amine loading is a key parameter that determines further reactivity towards mMPIO formation. Samples with low amine content cannot produce mMPIO, this minimum value is highly dependent of the dextran employed.

Product	Peptide loading (μmol/mg Fe)	% loading
**h**	1.65	23.1
**i**	1.68	27.3
**j**	0.77	20.2
**k**	2.53	22.4
**l**	nd	nd

Supplementary Table 3. Peptide loading of different peptido-NPs.

Final peptide coverage does not differ regardless to the amino content of the parent amino terminated iron oxide nanoparticles. nd = not determined.

	mMPIO-NH_2		mMPIO-COOH	
**Product**	**Ratio**[a]	**Size (nm)** [b]	**Ratio**[a]	**Size (nm)** [b]
**h**	6:1	588	1:6	670
**i**	6:1	535	1:6	587
**j**	6:1	722	1:6	498
**k**	--	--	1:10	519[c]
**l**	--	--	1:6	601

Supplementary Table 4. Final particle size during synthesis of mMPIO.

[a] ratio between peptido-NPs and carboxy-NPs. [b] determined by volume distribution of dynamic light scattering measurements. [c] When a 1:6 ratio was employed the product precipitated during synthesis.

Degradation studies of the peptidic linker

	**Cathepsin L**
Peptide **3**	active
Peptide **4**	×
Peptide **5**	×
Peptide **6**	×
Peptide **7**	×

Supplementary Table 5. Activity assay of peptides with cathepsin L.

The assay condition is described in the methods section. The reaction mixture was analysed by HPLC to detect the formation of products from the overnight reaction.

List of experiments performed with mMPIO

	$mMPIO-NH_2$	mMPIO-COOH
**TEM images**		Fig. 3b, Suppl. Fig. 18
**Sedimentation**	Suppl. Figs. 19-20	
**Relaxation rates**	Suppl. Fig. 21	
**Degradation microscopy**	Suppl. Fig. 24	Suppl. Figs. 22, 23-25
**Degradation TEM**		Suppl. Fig. 27
**Biodistribution mMPIO**	Suppl. Figs. 33-34	
**Biodistribution VCAM-mMPIO**		Suppl. Fig. 35
**Antibody loading determination**		Suppl. Fig. 37

Supplementary Table 6. List of experiments performed with mMPIO.

mMPIO physicochemical properties

Particles	4.7T T_2 Relaxivity ($mM^{-1}s^{-1}$)	7T T_2 Relaxivity ($mM^{-1}s^{-1}$)	4.7T T_1 Relaxivity ($mM^{-1}s^{-1}$)	7T T_1 Relaxivity ($mM^{-1}s^{-1}$)
**Dynabeads**	58.46 ± 2.16	59.53 ± 1.19	0.024 ± 0.004	0.015 ± 0.009
**mMPIO**	194.5 ± 4.5	196.5±4.9	0.949 ± 0.017	0.642±0.009
**MPIO**	251.8 ± 4.9	225.2 ± 4.0	0.6189 ± 0.014	0.3647 ± 0.011

Supplementary Table 7. T_1 and T_2 relaxivities of Dynabeads, mMPIO and monoMPIO at 4.7 and 7T field strength.

Errors are expressed as mean±s.d of three samples.

Compound[a]	Size (nm)	Core size (nm)	Number of cores per particle	Ms (emu/g)	R_2 ($mM^{-1}s^{-1}$)	Relaxivity per particle[b] ($L*bead^{-1}*s^{-1}$)	Relative relaxivity per particle[c]
mMPIO	500-700	9-14	~1000	--	195	2.58E-13	57461
CLIO[17]	25-35	4-7	1	63.8	55	1.19E-17	2.65
MD3[18]	44-74	10-15	1	--	185	3.14E-16	70
AMI 227[19] Sinerem Combidex	20-50	4-5	1	94.8	53.1	4.49E-18	1

Supplementary Table 8. Comparison between relaxivity per bead of different particles.

mMPIO possess equivalent R_2 values than particles with similar iron core size but due the larger number of cores contained in each mMPIO the T_2 contrast effect per particle is orders of magnitude larger. [a] Name of the compound and reference from which the data has been taken. [b] Assuming 40.5 metal atoms/nm^3 of core.[20] [c] Relative to Sinerem.

mMPIO biodistribution and toxicological studies

Sex: Female		Urea mg/dl (Day Number)	Cren mg/dl (Day Number)	Na mmol/l (Day Number)	K mmol/l (Day Number)	ALT U/l (Day Number)	AST U/l (Day Number)	ALP U/l (Day Number)
Group		2	2	2	2	2	2	2
Group: 1 Control 0 mg/kg	Mean	35.13	0.078	147.0	3.68	41.7	64.8	166.3
	SD	4.60	0.008	2.1	0.41	29.5	22.3	33.9
	N	6	5	5	5	6	6	6
Group: 2 VCAM-mMPIO 1.16 mg/kg	Mean	39.28	0.075	150.0	3.73	42.7	68.2	138.3
	SD	5.35	0.010	1.4	0.50	19.6	27.3	25.7
	N	6	6	6	6	6	6	6

Sex: Female		BiliT mg/dl (Day Number)	T. Prot g/dl (Day Number)	Alb g/dl (Day Number)	Glob g/dl (Day Number)	A/G ratio (Day Number)	Ca mg/dl (Day Number)	Chol mg/dl (Day Number)
Group		2	2	2	2	2	2	2
Group: 1 Control 0 mg/kg	Mean	0.090	4.82	3.42	1.40	2.45	10.30	106.3
	SD	0.000	0.23	0.17	0.13	0.22	0.32	19.7
	N	5	6	6	6	6	6	6
Group: 2 VCAM-mMPIO 1.16 mg/kg	Mean	0.090	4.95	3.47	1.48	2.35	10.52	103.8
	SD	0.000	0.14	0.05	0.13	0.23	0.31	17.6
	N	6	6	6	6	6	6	6

Sex: Female		Gluc mg/dl (Day Number)
Group		2
Group: 1 Control 0 mg/kg	Mean	211.2
	SD	39.2
	N	6
Group: 2 VCAM-mMPIO 1.16 mg/kg	Mean	217.7
	SD	16.0
	N	6

Supplementary Table 9. Blood chemistry analysis 2 days post-αhuVCAM-mMPIO injection.

Statistical analysis showed no significant differences between groups with unpaired, two-tailed *t*-tests with a Bonferroni correction. The abbreviations employed are: Cren – creatinine; Na – sodium; K – potassium; ALT- alanine transaminase; AST - aspartate transaminase; ALP - alkaline phosphatase; BiliT- total bilirubin; T. Prot- total protein; Alb – albumin; Glob- Globulin; A/G ratio - albumin to globulin ratio; Ca – calcium; Chol – cholesterol; Gluc – Glucose.

Sex: Female		Urea mg/dl (Day Number)	Cren mg/dl (Day Number)	Na mmol/l (Day Number)	K mmol/l (Day Number)	ALT U/l (Day Number)	AST U/l (Day Number)	ALP U/l (Day Number)
Group		14	14	14	14	14	14	14
Group: 1 Control 0 mg/kg	Mean	35.70	0.073	149.5	3.35	22.8	60.0	139.3
	SD	7.20	0.005	0.8	0.15	4.1	14.8	32.9
	N	6	6	6	6	6	6	6
Group: 2 VCAM-mMPIO 1.16 mg/kg	Mean	44.08	0.082	149.8	3.38	18.3	44.7	148.2
	SD	7.06	0.012	1.0	0.27	2.0	4.0	11.4
	N	6	6	6	6	6	6	6

Sex: Female		BiliT mg/dl (Day Number)	T. Prot g/dl (Day Number)	Alb g/dl (Day Number)	Glob g/dl (Day Number)	A/G ratio (Day Number)	Ca mg/dl (Day Number)	Chol mg/dl (Day Number)
Group		14	14	14	14	14	14	14
Group: 1 Control 0 mg/kg	Mean	0.090	4.95	3.42	1.53	2.22	9.97	105.2
	SD	0.000	0.26	0.19	0.08	0.10	0.12	7.3
	N	6	6	6	6	6	6	6
Group: 2 VCAM-mMPIO 1.16 mg/kg	Mean	0.090	4.93	3.40	1.53	2.23	10.00	106.2
	SD	0.000	0.22	0.15	0.14	0.20	0.26	16.4
	N	6	6	6	6	6	6	6

Sex: Female		Gluc mg/dl (Day Number)
Group		14
Group: 1 Control 0 mg/kg	Mean	221.8
	SD	26.8
	N	6
Group: 2 VCAM-mMPIO 1.16 mg/kg	Mean	207.7
	SD	26.7
	N	6

Supplementary Table 10. Blood chemistry analysis 14 days post-αhuVCAM-mMPIO injection.

Statistical analysis showed no significant differences between groups with unpaired, two-tailed *t*-tests with a Bonferroni correction. The abbreviations employed are: Cren – creatinine; Na – sodium; K – potassium; ALT- alanine transaminase; AST - aspartate transaminase; ALP - alkaline phosphatase; BiliT- total bilirubin; T. Prot- total protein; Alb – albumin; Glob- Globulin; A/G ratio - albumin to globulin ratio; Ca – calcium; Chol – cholesterol; Gluc – Glucose.

Sex: Female		RBC 10^6/ul (Day Number)	Hb g/dl (Day Number)	PCV % (Day Number)	MCV fl (Day Number)	MCH pg (Day Number)	MCHC g/dl (Day Number)	Plate 10^3/ul (Day Number)
Group		2	2	2	2	2	2	2
Group: 1 Control 0 mg/kg	Mean	8.187	13.53	44.20	54.12	16.57	30.63	1269.2
	SD	0.716	1.04	3.57	3.24	0.86	0.38	119.4
	N	6	6	6	6	6	6	5
Group: 2 VCAM-mMPIO 1.16 mg/kg	Mean	8.097	13.27	43.95	54.17	16.35	30.22	1045.0
	SD	0.575	1.10	4.03	1.40	0.20	0.72	301.4
	N	6	6	6	6	6	6	6

Sex: Female		Retics %RBC (Day Number)	WBC 10^3/ul (Day Number)	Neut 10^3/ul (Day Number)	Lymph 10^3/ul (Day Number)	Mono 10^3/ul (Day Number)	Eosin 10^3/ul (Day Number)	Baso 10^3/ul (Day Number)
Group		2	2	2	2	2	2	2
Group: 1 Control 0 mg/kg	Mean	4.70	5.950	0.757	4.885	0.100	0.152	0.007
	SD	0.85	2.018	0.200	1.784	0.057	0.062	0.005
	N	6	6	6	6	6	6	6
Group: 2 VCAM-mMPIO 1.16 mg/kg	Mean	4.53	4.517	0.653	3.610	0.075	0.097	0.002
	SD	1.27	1.181	0.250	1.127	0.043	0.055	0.004
	N	6	6	6	6	6	6	6

Sex: Female		LUC 10^3/ul (Day Number)	Neut % (Day Number)	Lymph % (Day Number)	Mono % (Day Number)	Eosin % (Day Number)	Baso % (Day Number)	LUC % (Day Number)
Group		2	2	2	2	2	2	2
Group: 1 Control 0 mg/kg	Mean	0.047	13.27	81.68	1.60	2.62	0.12	0.73
	SD	0.029	2.89	3.63	0.37	0.92	0.08	0.36
	N	6	6	6	6	6	6	6
Group: 2 VCAM-mMPIO 1.16 mg/kg	Mean	0.077	14.97	79.37	1.63	2.23	0.08	1.73
	SD	0.014	5.73	7.15	0.72	1.24	0.04	0.31
	N	6	6	6	6	6	6	6

Supplementary Table 11. Haematology analysis 2 days post-αhuVCAM-mMPIO injection.

Statistical analysis showed no significant differences between groups with unpaired, two-tailed *t*-tests with a Bonferroni correction, with the exception of %LUC (large unstained cells), which showed a significant increase at this time point. However, the absolute number of LUC was not significantly different between the groups and, moreover, all values for both number LUC and %LUC were within normal ranges (0-0.34 x10^3/µl and 0-3%, respectively) for all animals. No other subsets of leukocytes showed any significant changes. The abreviations employed are: RBC - red blood cells; Hb - haemoglobin; PCV - packed cell volume; MCV - mean corpuscular volume; MCH - mean corpuscular haemoglobin; MCHC - mean corpuscular haemoglobin concentration; Plate - platelets; Retics - reticulocytes; WBC- white blood cells; Neut - neutrophils; Lymph - lymphocytes; Mono - monocytes; Eosin - eosinophils; Baso - basophils.

Sex: Female		RBC 10^6/ul (Day Number)	Hb g/dl (Day Number)	PCV % (Day Number)	MCV fl (Day Number)	MCH pg (Day Number)	MCHC g/dl (Day Number)	Plate 10^3/ul (Day Number)
Group		14	14	14	14	14	14	14
Group: 1 Control 0 mg/kg	Mean	8.902	14.04	43.72	52.18	15.80	30.24	1049.2
	SD	0.223	0.44	6.69	1.03	0.64	0.99	177.2
	N	5	5	6	5	5	5	6
Group: 2 VCAM-mMPIO 1.16 mg/kg	Mean	9.408	14.64	44.13	52.30	15.64	29.86	872.2
	SD	0.585	0.38	12.66	1.21	1.02	1.74	193.6
	N	5	5	6	5	5	5	6

Sex: Female		Retics %RBC (Day Number)	WBC 10^3/ul (Day Number)	Neut 10^3/ul (Day Number)	Lymph 10^3/ul (Day Number)	Mono 10^3/ul (Day Number)	Eosin 10^3/ul (Day Number)	Baso 10^3/ul (Day Number)
Group		14	14	14	14	14	14	14
Group: 1 Control 0 mg/kg	Mean	2.82	6.907	0.933	5.635	0.088	0.150	0.008
	SD	0.44	2.487	0.201	2.269	0.035	0.053	0.008
	N	6	6	6	6	6	6	6
Group: 2 VCAM-mMPIO 1.16 mg/kg	Mean	2.70	5.612	0.777	4.520	0.070	0.155	0.003
	SD	0.57	3.080	0.578	2.451	0.034	0.124	0.005
	N	6	6	6	6	6	6	6

Sex: Female		LUC 10^3/ul (Day Number)	Neut % (Day Number)	Lymph % (Day Number)	Mono % (Day Number)	Eosin % (Day Number)	Baso % (Day Number)	LUC % (Day Number)
Group		14	14	14	14	14	14	14
Group: 1 Control 0 mg/kg	Mean	0.090	14.68	80.43	1.30	2.15	0.12	1.30
	SD	0.041	5.32	5.57	0.44	0.48	0.04	0.21
	N	6	6	6	6	6	6	6
Group: 2 VCAM-mMPIO 1.16 mg/kg	Mean	0.085	14.35	80.43	1.30	2.57	0.05	1.28
	SD	0.077	5.54	5.66	0.38	0.69	0.05	0.66
	N	6	6	6	6	6	6	6

Supplementary Table 12. Haematology analysis 14 days post-αhuVCAM-mMPIO injection.

Statistical analysis showed no significant differences between groups with unpaired two-tailed *t*-tests with a Bonferroni correction. Note the decrease in %LUC observed at 2 days post-αhuVCAM-mMPIO administration is no longer evident at this time point. The abreviations employed are: RBC - red blood cells; Hb - haemoglobin; PCV - packed cell volume; MCV - mean corpuscular volume; MCH - mean corpuscular haemoglobin; MCHC - mean corpuscular haemoglobin concentration; Plate - platelets; Retics - reticulocytes; WBC- white blood cells; Neut - neutrophils; Lymph - lymphocytes; Mono - monocytes; Eosin - eosinophils; Baso - basophils.

Characterization of Peptides

		Theoretical Fragment Ion					
		A	B	C	X	Y	Z
**Fmoc-Ahx**	**1**	308.1645	336.1594	353.186		1182.63	1165.604
**Ahx**	**2**	421.2485	449.2434	466.27	873.4575	847.4782	830.4516
**F**	**3**	568.3169	596.3118	613.3384	760.3735	734.3942	717.3676
**V**	**4**	667.3853	695.3802	712.4068	613.3051	587.3258	570.2992
**R**	**5**	823.4864	851.4813	868.5079	514.2367	488.2574	471.2308
**G**	**6**	880.5078	908.5027	925.5293	358.1356	332.1563	315.1297
**A**	**7**	951.5449	979.5398	996.5664	301.1142	275.1349	258.1083
**G**	**8**	1008.566	1036.561	1053.588	230.0771	204.0978	187.0712
**E**	**9**	1136.625	1164.62	1181.646	173.0557	147.0764	130.0498
		**Observed Fragment Ion**					
		**A**	**B**	**C**	**X**	**Y**	**Z**
**Fmoc-Ahx**	**1**		336.145	353.538		1182.397	
**Ahx**	**2**						830.204
**F**	**3**	567.973	596.049			734.054	
**V**	**4**		695.174	712.232		587.034	
**R**	**5**	823.258	851.247				
**G**	**6**	880.228		925.416			
**A**	**7**	951.394					
**G**	**8**	1008.453					
**E**	**9**		1164.612				

Supplementary Table 13. Predicted masses and observed fragment ions detected from MALDI MS/MS analysis of peptide 3.

	Theoretical Fragment Ion						
		**A**	**B**	**C**	**X**	**Y**	**Z**
**Fmoc-Ahx**	**1**	308.1645	336.1594	353.186		1182.63	1165.604
**Ahx**	**2**	421.2485	449.2434	466.27	873.4575	847.4782	830.4516
**F**	**3**	568.3169	596.3118	613.3384	760.3735	734.3942	717.3676
**V**	**4**	667.3853	695.3802	712.4068	613.3051	587.3258	570.2992
**R**	**5**	823.4864	851.4813	868.5079	514.2367	488.2574	471.2308
**G**	**6**	880.5078	908.5027	925.5293	358.1356	332.1563	315.1297
**A**	**7**	951.5449	979.5398	996.5664	301.1142	275.1349	258.1083
**G**	**8**	1008.566	1036.561	1053.588	230.0771	204.0978	187.0712
**E**	**9**	1136.625	1164.62	1181.646	173.0557	147.0764	130.0498
		**Observed Fragment Ion**					
		**A**	**B**	**C**	**X**	**Y**	**Z**
**Fmoc-Ahx**	**1**		336.006	353.396		1182.381	
**Ahx**	**2**					847.033	829.820
**F**	**3**						
**V**	**4**			712.042		586.845	
**R**	**5**	823.027	850.997				
**G**	**6**	880.028		925.125			
**A**	**7**	951.143					
**G**	**8**	1008.204					
**E**	**9**						

Supplementary Table 14. Predicted masses and observed fragment ions detected from MALDI MS/MS analysis of peptide 4.

		Theoretical Fragment Ion					
		A	B	C	X	Y	Z
Fmoc-Ahx	1	308.1645	336.1594	353.186		1182.63	1165.604
Ahx	2	421.2485	449.2434	466.27	873.4575	847.4782	830.4516
F	3	568.3169	596.3118	613.3384	760.3735	734.3942	717.3676
V	4	667.3853	695.3802	712.4068	613.3051	587.3258	570.2992
R	5	823.4864	851.4813	868.5079	514.2367	488.2574	471.2308
G	6	880.5078	908.5027	925.5293	358.1356	332.1563	315.1297
A	7	951.5449	979.5398	996.5664	301.1142	275.1349	258.1083
G	8	1008.566	1036.561	1053.588	230.0771	204.0978	187.0712
E	9	1136.625	1164.62	1181.646	173.0557	147.0764	130.0498
		Observed Fragment Ion					
		A	B	C	X	Y	Z
Fmoc-Ahx	1		335.947	353.295		1182.318	
Ahx	2			466.034			
F	3	567.710	595.780			733.805	
V	4		694.898	711.917		586.758	
R	5	822.885	850.867				
G	6	879.797					
A	7	950.990					
G	8						
E	9		1164.162				

Supplementary Table 15. Predicted masses and observed fragment ions detected from MALDI MS/MS analysis of peptide 5.

		Theoretical Fragment Ion					
		A	B	C	X	Y	Z
**Fmoc-Ahx**	**1**	308.1645	336.1594	353.186		1090.568	1073.541
**Ahx**	**2**	421.2485	449.2434	466.27	781.3948	755.4155	738.3889
**G**	**3**	478.2699	506.2648	523.2914	668.3108	642.3315	625.3049
**P**	**4**	575.3226	603.3175	620.3441	611.2894	585.3101	568.2835
**R**	**5**	731.4237	759.4186	776.4452	514.2367	488.2574	471.2308
**G**	**6**	788.4451	816.44	833.4666	358.1356	332.1563	315.1297
**A**	**7**	859.4822	887.4771	904.5037	301.1142	275.1349	258.1083
**G**	**8**	916.5036	944.4985	961.5251	230.0771	204.0978	187.0712
**E**	**9**	1044.562	1072.557	1089.584	173.0557	147.0764	130.0498
		**Observed Fragment Ion**					
		**A**	**B**	**C**	**X**	**Y**	**Z**
**Fmoc-Ahx**	**1**		336.485			1090.487	
**Ahx**	**2**						738.165
**G**	**3**		506.220	523.206		642.192	
**P**	**4**		603.219			585.211	
**R**	**5**	731.218	759.230			488.135	471.136
**G**	**6**	788.209		833.216			
**A**	**7**	859.320					
**G**	**8**	916.290					
**E**	**9**						

Supplementary Table 16. Predicted masses and observed fragment ions detected from MALDI MS/MS analysis of peptide 6.

		Theoretical Fragment Ion					
		A	B	C	X	Y	Z
**Fmoc-Ahx**	**1**	308.1645	336.1594	353.186		1147.662	1130.635
**Ahx**	**2**	421.2485	449.2434	466.27	838.4891	812.5098	795.4832
**I**	**3**	533.3485	561.3434	578.37	725.4051	699.4258	682.3992
**V**	**4**	632.4169	660.4118	677.4384	613.3051	587.3258	570.2992
**R**	**5**	788.518	816.5129	833.5395	514.2367	488.2574	471.2308
**A**	**6**	859.5551	887.55	904.5766	358.1356	332.1563	315.1297
**G**	**7**	916.5765	944.5714	961.598	287.0985	261.1192	244.0926
**G**	**8**	973.5979	1001.593	1018.619	230.0771	204.0978	187.0712
**E**	**9**	1101.656	1129.651	1146.678	173.0557	147.0764	130.0498
		**Observed Fragment Ion**					
		**A**	**B**	**C**	**X**	**Y**	**Z**
**Fmoc-Ahx**	**1**		336.257				
**Ahx**	**2**						
**I**	**3**			578.331			
**V**	**4**					587.311	
**R**	**5**					487.888	470.753
**A**	**6**						315.287
**G**	**7**						
**G**	**8**					204.207	
**E**	**9**				173.328		

Supplementary Table 17. Predicted masses and observed fragment ions detected from MALDI MS/MS analysis of peptide 7.

Supplementary Methods

General

Resins, protected aminoacids and coupling reagents for peptide synthesis were purchased from GL Biochem (China) or Sigma Aldrich (UK). Solvents were purchased from Rathburn (UK), Sigma-Aldrich (UK) and Fisher Scientific (UK). Primary antibodies were purchased from Southern Biotech (USA). Fluorophores, Dynabeads Talon and fluorescently labelled secondary antibodies were purchased from Invitrogen (UK). Cells were tested for mycoplasma infection on a quarterly 409 basis using Lonza MycoAlert™ Mycoplasma Detection Kit. All other chemicals were purchased from Sigma-Aldrich (UK). All chemicals and solvents were used without further purification. Solid Phase Peptide Synthesis was performed on a CEM Liberty peptide synthesizer. Analytical reverse phase HPLC spectra was performed using a Dionex Ultimate 3000 system and a Jupiter 4 µm Proteo 90Å 250 x 4.6 mm analytical column (Phenomenex, UK) at a flow rate of 1 mL/min. Reverse phase preparative separations were performed on a Biotage SP4 automated chromatography system with UV monitoring, using a C18 Flash 25+M cartridge or a C18 SNAP 400g cartridge. In all cases buffer A was 0.1% TFA in water and buffer B was 0.1% TFA in acetonitrile. High-resolution mass spectra (HRMS) were recorded on a Bruker MicroTOF ESI mass spectrometer. Nominal and exact m/z values are reported in Daltons. Addition of compounds was done by a Kazel A99 FZ syringe pump. Liquid chromatography-mass spectrometry (LC-MS) was performed on a Waters ZQ2000 (ESI-TOF-MS) coupled to a Waters W2695 HPLC using a ACE 3 µm 150 x 3.0 mm column (Advanced Chromatography Technologies Ltd., UK) at a flow rate of 0.5 mL/min and a Waters W474 fluorescence detector or on a Waters QuattroMicro-MS operating in ESI^+ mode coupled to a Waters 1525µ HPLC pump and a Waters 2777 CTC auto-sampler using a C12 Phenomenex Jupiter Proteo 90Å 4 µm 250x4.60 mm column at a flow rate of 1 mL/min. Buffer A, 10 mM formic acid in water, and buffer B, 10 mM formic acid in acetonitrile, were used as the mobile phase. The electrospray source of LCT was operated with a capillary voltage of 3.5 kV and a cone voltage of 20 V. Nitrogen was used as the nebulizer and desolvation gas at a total flow of 450 L/h. Dynamic light

scattering (DLS) measurements were performed in a Malvern Zetasizer 3000 HS or Zetasizer Nano ZS using intensity weighted distributions for nanoparticles and volume weighted distributions for mMPIO. Zeta potential was measured on a Malvern Instrument Zetasizer–Nano ZS. The measuring was done using following parameters: temperature, 23 °C; liquid viscosity, 0.887 cP; refraction index, 1.33; external fibre angle, 90°. In addition to those specified above, the following abbreviations and designations are used throughout the Supporting Information: AF488 = Alexa Fluor 488, aq. = aqueous, AFM = atomic force microscopy, carboxy-NP = carboxylic acid terminated nanoparticles, DAPI = 4′,6-diamidino-2-phenylindole dihydrochloride, DIC = differencial interference contrast, DMEM = Dubelcco's modified eagle medium, DMF = *N*,*N*-dimethylformamide, DMSO = dimethylsulfoxide, DTT = DL-dithiothreitol, EDC = *N*-(3-dimethylaminopropyl)-*N*′-ethylcarbodiimide hydrochloride, EDTA = ethylenediaminetetraacetic acid, EDX = energy-dispersive X-ray spectroscopy, ESI = electrospray ionization, FBS = fetal bovine serum, HBTU = *O*-(benzotriazol-1-yl)-*N,N,N',N'*-tetramethyluronium hexafluorophosphate, HOBt = 1-hydroxybenzotriazole hydrate, HPLC= high pressure liquid chromatography, HRMS = high resolution mass spectroscopy, IL-1β = interleukin-1 beta, LC-MS = liquid chromatography- mass spectrometry, MALDI = Matrix-assisted laser desorption/ionization, MES = 2-(N-morpholino)ethanesulfonic acid, mMPIO = multimeric microparticles of iron oxide, mMPIO-COOH = carboxylic acid covered mMPIO, mMPIO-NH_2 = amino covered mMPIO, MPIO = microparticles of iron oxide, MPIO-NH_2 = amino covered MPIO, MS/MS = tandem mass spectroscopy, MWCO = molecular weight cut-off, NHS = *N*-hydroxysuccinimide , NP = amino covered nanoparticles , NRS = normal rabbit serum, PBS = phosphate buffer saline, peptido-NP = peptide covered iron oxide nanoparticles, PTFE = polytetrafluoroethylene, RP (chromatography) = reverse phase (chromatography), rpm = revolutions per minute, rt = room temperature, SDP = 3,5-dichloro-4-hydroxybenzenesulfonic acid, SPPS = solid phase peptide synthesis, sulfo-NHS = *N*-hydroxysulfosuccinimide, TE = echo time, TEM = transmission electron microscopy, TFA = trifluoroacetic acid, TIS = triisopropylsilane, TNB = Tris- NaCl blocking buffer, TNF-α = tumor necrosis factor-alpha, TR = repetition time, Tris-HCl = Tris(hydroxymethyl)aminomethane hydrochloride, VCAM1 = vascular cell adhesion molecule 1.

Determination of elemental composition of NPs

Experiment was performed by triplicate. A solution of nanoparticles containing 5 mg of iron was freeze dried in a weighted tube. Dry sample was weighted and %Fe was calculated according to the formula %Fe = 5/dry sample weight * 100. Dry sample was subjected to CH&N determination by combustion analysis. From the data, amine content (µmol N/mg Fe) was calculated according to the formula (%N/14)*1000/%Fe.

Determination of peptide content in peptido-NPs

100 µL of the crude peptide-NP synthesis was precipitated by addition of 1000 µL of 1,4-dioxane, centrifuged at 17000 g for 30 minutes and supernatant was discarded. The pellet was redispersed in 100 µL of DMSO and precipitated with 1000 µL of 1,4-dioxane, centrifuged at 17000 g for 30 minutes and supernatant was discarded. Pellet was redispersed in 100 µL of DMSO, 3 aliquots (10 µL) were taken for iron concentration analysis as previously reported[21] and 3 aliquots (10 µL) were taken and treated with 10 uL of DMF/DBU 96:4 for 30 minutes, 980 µL of 1,4-dioxane was added and centrifuged at 17000 g for 30 minutes. Absorbance of the colourless supernatant was measured at 301nm and compared to a standard curve made by serial dilutions of Fmoc-Gly-OH in DMSO treated in the same way than peptide-NP samples. Peptide content was obtained by dividing Fmoc concentration previously determined by Fe concentration and % peptide loading was determined dividing the peptide content by the amine content of the amino-NPs precursor.

Purification of mMPIO by magnetic pelleting

mMPIO sample in PBS was pelleted in a magnetic separator (DynaMag Spin Magnet, Invitrogen, UK) for 30 minutes, supernatant was discarded and pellet was resuspended in 1 mL PBS containing 0.1 % Tween 20. Sample was shaken at 2000 rpm for at least 8 h and pelleted again for 30 minutes. This

purification process was repeated 3 times and sample was finally resuspended in the desired amount of PBS.

Purification of mMPIO by sucrose gradient

Sucrose solutions of 70%, 65%, 60%, 55%, 45% and 40% (w/v) in 10 mM PBS buffer pH 7.0 were prepared and used within 2 weeks. Gradients were prepared by underlying sucrose solutions of decreasing density (1.4 mL) in a centrifuge tube of 5 mL, and carefully overlaying the sample (0.5 mg of the mMPIO suspended in PBS) to fill the tube (normally 400 μL). Gradients were ultracentrifuged in a Optima MAX-XP (Beckam Coulter, UK) using a swing–out rotor (SW40Ti) and centrifuged at 500 g for 15 minutes at 4 °C to eliminate large aggregates > 2 μm. The top 2 mL of the gradient tube was carefully transferred to the fresh gradients with 70%, 65%, 55%, 50% and 45%. Gradient then centrifuged at 17600 g for 20 min at 4 °C. 1 mL fractions containing the particles were collected and buffer exchanged for 10 mM PBS buffer pH 7.0. Particle size was then investigated by DLS and NTA.

Degradation of fluorescent peptides by cathepsins

Cathepsin B (546 pmol, 300 mU) or cathepsin L (30 pmol, 2 mU) in 98 μL of citrate buffer 0.1M pH 5.5 containing 1 mM EDTA was activated by addition of DTT (2 μL, 100 mM in water) for 15 minutes at room temperature. The fluorescent peptides **1** and **2** (40 μL, 0.43 mM) in DMSO was added to a solution of the protease in 1460 μL of citrate buffer 0.1M pH 5.5 containing 1 mM of EDTA and 2 mM of DTT. The final protease concentration was 20 nM for cathepsin B and 3 nM for cathepsin L. The sample was incubated at 37 ºC with continuous shaking and, at selected time points, 145 μL of that solution was taken, added to 5 μL of E64 solution (100 mM) in DMSO and 10 μL of the mixture was analyzed by HPLC-MS (25-45 % solvent B, fluorescence detector, Ex = 328 nm, Em = 400 nm; **Supplementary Figs. 5-8**).

Stability of fluorescent peptides in human plasma and serum

Plasma: A solution of the fluorescent peptides **1** and **2** (4.6 µL, 1.25 mg/mL) in DMSO was added to 200 µL of human plasma (Sigma-Aldrich, P9523). The samples were incubated at 37 °C. At selected time points 50 µL of this solution was added to 100 µL of acetonitrile/water/formic acid 70:30:0.1 and 2 µL of the mixture was analyzed by HPLC (25-45 % solvent B, fluorescence detector, Ex = 328 nm, Em = 400 nm; **Supplementary Figs. 9-10**). As control the same quantity of peptide was added to 200 µL of PBS and treated as the plasma samples.

Serum: A solution of the fluorescent peptides **1** and **2** (4.6 µL, 1.25 mg/mL) in DMSO was added to 200 µL of human serum). The samples were incubated at 37 °C. At selected time points 50 µL of this solution was added to 100 µL of acetonitrile/water/formic acid 70:30:0.1, sample was centrifuged at 17000 g for 10 min and 2 µL of the mixture was analysed by HPLC (25-45 % solvent B, fluorescence detector, Ex = 328 nm, Em = 400 nm; **Supplementary Figs. 11-12**). As control the same quantity of peptide was added to 200 µL of PBS and treated as the serum samples.

A human serum sample (200 µL) was also treated with (a) hirudin (SigmaAldrich, Cat. Nr. 94581) at a final concentration of 200 µg/mL, (b) EDTA at a final concentration of 10 mM, (c) heparin sodium salt (Applichem, Cat. Nr. 3004) at a final concentration of 10 IU/mL and (d) complete mini EDTA free (Roche, Cat. Nr. 11836170001) for 30 min. at room temperature, or heated at 95 ºC for 10 minutes and then 4.6 µL of solution of the fluorescent peptide **1** (1.25 mg/mL) in DMSO was added. The samples were incubated at 37 °C. At selected time points 50 µL of this solution was added to 100 µL of acetonitrile/water/formic acid 70:30:0.1, sample was centrifuged at 17000 g for 10 min and 2 µL of the mixture was analysed by HPLC (25-45 % solvent B, fluorescence detector, Ex = 328 nm, Em = 400 nm; **Supplementary Fig. 13**). As control the same quantity of peptide was added to 200 µL of serum with no additives and treated as the rest of samples.

Degradation of non-fluorescent peptides by cathepsin L

A solution of cathepsin L (20 μg/mL, 24 units/L) in citrate buffer 0.1 M pH 5.5, 1 mM EDTA, 2 mM DTT was preactivated at 25 °C for 15 minutes. A stock solution of peptide **4, 5, 6** or **7** (125 μL, 25 nmol, 0.20 mM in DMSO) was diluted in 365 μL of the assay buffer to a final concentration of 50 μM and pre-incubated at 37 °C. 10 μL of pre-activated cathepsin L was then added to the peptide solution and incubated with mild shaking at 37 °C. Aliquots of 100 μL were withdrawn at 0 min, 1 hour and overnight. Samples were flash-frozen on liquid nitrogen and were heated at 100 °C for 120 seconds prior to the analyses by HPLC (**Supplementary Figures 14-17** and **Supplementary Table 5**).

Transmission electron microscopy (TEM)

5–10 μl of a sucrose-purified particle suspension (0.1 mg Fe/ml) in Milli-Q water were allowed to settle briefly (1 minute) onto pyroxylin and carbon–coated copper grids (400 mesh, Agar Scientific), and then blotted dry with filter paper. For negative staining 2% (w/v), uranyl acetate was used; the excess solution was removed with filter paper, and the grids left to air-dry until needed. Grids were viewed at 80 Kv in an FEI Tecnai12 TEM (FEI UK Ltd, Cambridge) and images were obtained using a bottom–mounted AMT XR60 CCD camera (Deben UK Ltd, Bury St. Edmunds).

Synthesis of 733 nm amino terminated particles (MPIO)

10 mmol of $FeCl_3 \cdot 6H_2O$ and 3.0 g of dextran (MW 11000) were dissolved in 20 mL of water and deoxygenated thoroughly by repeated cycles of vacuum assisted by sonication and argon flushing. After the first deoxygenation cycle, 6 mmol of $FeCl_2 \cdot 4H_2O$ in 5 mL of water was added and the solution was deoxygenated by the above procedure (4 times). Whilst being stirred with an overhead stirrer at 600 rpm, 8mL of NH_4OH (25%) were added at a rate of 168 mL/h. The reaction was heated to 80 ºC and then

stirred at that temperature for 1 hour. The solution was cooled, dialyzed against water in a Spectra/Por membrane (MWCO 100000) for 21 h.

10 mL of the above synthesized particles (5.7 mg/mL) were loaded into a 250 mL round flask equipped with a 30x16 mm oval stirrer bar. While the solution was stirred at 500 rpm, 5 mL of NaOH (aq. 10 M) were added at a rate of 168 mL/h. After that, 4 mL of epichlorohydrin was added at a rate of 96 mL/h. The mixture was stirred at 1000 rpm for 7h and then 7 mL of NH_4OH (25%) was added at a rate of 168 mL/h. The mixture was stirred at 1000 rpm for 14 hours. The mixture was then dialysed against water in a Spectra/Por 2 membrane and then concentrated on a Vivaspin 15 unit (30000 MCWO) to 15 mg Fe/mL.

Synthesis of fluorescent labelled mMPIOs for macrophage studies

A solution of sulfoNHS (5 μL, 1 M) in water and a solution of Alexa Fluor 488 cadaverine (10 μL, 2 mg/mL) in DMSO were sequentially added to a solution of mMPIO (mMPIO-COOH or mMPIO-NH_2) (500 μL, 1 mg Fe/mL) in bicarbonate buffer 0.1 M pH 8.3. The sample was shaken for 30 minutes and, after that time, a solution of EDC (1 μL, 1 M) was added. The sample was shaken for 24 h, after which particles were collected using a magnet (Invitrogen) and the supernatant was discarded. The particles were washed 5 times with 1 mL of PBS buffer containing 0.1% of Tween 20 and once with 1 mL of PBS buffer, and re-dissolved in 200 μL of PBS buffer.

Synthesis of fluorescent labelled MPIO for macrophage studies

A solution of Alexa Fluor 488 carboxylic acid succinimidyl ester (2 μL, 10 mg/mL) in DMSO was added to a solution of MPIO-NH_2 (400 μL, 1 mg Fe/mL) in bicarbonate buffer 0.1 M pH 8.3. The sample was shaken for 30 minutes and, after that time, a solution of EDC (1 μL, 1 M) was added. The sample was shaken for 24 hours, after which particles were collected using a magnet (Invitrogen) and the supernatant was discarded. The particles were washed 5 times with 1 mL of PBS buffer containing 0.1% of Tween 20 and once with 1 mL of PBS buffer, and re-dissolved in 200 μL of PBS buffer.

Macrophage uptake and mMPIO degradation studies

Freshly isolated murine monocytes were differentiated into macrophages by culturing overnight in DMEM supplemented with 10% FBS in glass bottomed 35 mm culture dishes (SPL Life Sciences, Korea). Particles were added to the culture medium and images taken at selected time points using a cooled monochrome QICAM, coupled to an Olympus IX-71 inverted microscope fitted with a 100X, 1.3NA oil-immersion lens. Single-frame fluorescence images using a filter set for Alexa Fluor 488 and differential interference contrast (DIC) images were taken for each time point and overlaid to demonstrate position of particles relative to the cells (**Supplementary Figs. 24-25**)

Macrophage sectioning and TEM imaging

Raw 264.7 cells were grown and treated in 24-well plates. After treatment, cells were fixed in a solution of 1.6% glutaraldehyde for 1 h at 4 °C. Fixed cells were washed three times with 2% paraformadehyde and 0.1% picric acid in 100 mM phosphate (pH 7.0). Post-fixation staining was done using 1% stock solution of osmium tetroxide in 100 mM phosphate (pH 7.0) for 1.5 h at 4ºC. Cells were washed with phosphate buffer 0.1 M, pH 7.4 and incubated with 2% uranyl acetate for 2 hours at room temperature. Cells were dehydrated in methanol (50, 70, 90 and 100% methanol) and infiltrated in Spurr's low viscosity (2:1, 1:1 and 1:2) resins. Then, cells were infiltrated in Agar 100 (as a pre-mix kit) resin for 12 h at 4 °C, embedded in Beem capsules containing pure resin blocks, and finally hardened at 70 °C for 17 hours (overnight). Ultrathin sections (70-90 nm) were cut (Leica EM UC7), and the sections were analysed under a 180 kV TEM microscope FEI Tecnai12 TEM (FEI UK Ltd, Cambridge) and images were obtained using a bottom–mounted AMT XR60 CCD camera (Deben UK Ltd, Bury St. Edmunds) (**Supplementary Figure 27**).

Synthesis of αhuVCAM-mMPIO for formal toxicology study

All the work was performed in sterile conditions. To 1.5 mL of mMPIO-COOH (1.35 mg Fe/mL, 2.0 mg) in 0.1 M MES buffer, a solution of sulfoNHS (53 μL, 0.1 M) in water and a solution of EDC (53 μL, 0.01 M) in water were sequentially added. The resultant solution was stirred for 5 minutes at rt and then 91 μL of a fully humanised antibody raised against the extracellular domain of human VCAM-1 (Antitope Ltd) (4.3 mg/mL) followed by 1.5 mL of bicarbonate buffer 0.1 M pH 8.3 was added. The sample was shaken for 24h. The sample was filtered through a 2-μm polycarbonate filter (Sterlitech, USA). The particles were purified by magnetic separation (**Supplementary Method 4**) and resuspended in PBS buffer.

αhuVCAM-mMPIO formal toxicology study (Sequani Ltd)

A single-dose, extended-acute study was carried out by the Commercial Research Organisation Sequani Ltd. on the mMPIO conjugated to a fully humanised anti-human VCAM-1 antibody (αhuVCAM-mMPIO). Two groups of 12 female Crl:CD-1 (ICR) mice were injected intravenously with either vehicle (phosphate buffered saline and 10% dextran w/v) or αhuVCAM-mMPIO at 1.2 mg Fe/kg body weight, with a dose volume of 1 mL/kg. Animals were killed at either 2 or 14 days after injection (n = 6 per group); blood and tissues were taken for analysis. The following assessments were made: body weight, organ weights, macroscopic pathology, haematology, blood chemistry and microscopic analysis of the brain (at three levels), heart, kidneys, liver, lungs and spleen. The same organs were assayed for iron deposition with Perls′ Prussian blue stain and nuclear fast red counterstain. Photomicrographs were captured of tissues at original magnifications of 40X (liver, lung and spleen) and 10X for brain and are shown at an image quality of 300 dpi.

For haematology and blood chemistry, αhuVCAM-mMPIO injected animals were compared to controls using a two-tailed t-test with a Bonferroni family-wise correction. In total, 36 comparisons were made for

haematological and blood chemistry measurements at each time point, and consequently results were considered significant at $p<0.0014$.

Supplementary References

1 Jefferson, A. *et al.* Molecular imaging with optical coherence tomography using ligand-conjugated microparticles that detect activated endothelial cells: rational design through target quantification. *Atherosclerosis* **219**, 579-587, (2011).

2 McAteer, M. A. *et al.* In vivo magnetic resonance imaging of acute brain inflammation using microparticles of iron oxide. *Nat. Med.* **13**, 1253-1258, (2007).

3 von zur Muhlen, C. *et al.* A contrast agent recognizing activated platelets reveals murine cerebral malaria pathology undetectable by conventional MRI. *J. Clin. Invest.* **118**, 1198-1207, (2008).

4 Serres, S. *et al.* VCAM-1-targeted magnetic resonance imaging reveals subclinical disease in a mouse model of multiple sclerosis. *The FASEB Journal* **25**, 4415-4422, (2011).

5 von zur Muhlen, C. *et al.* Visualization of Activated Platelets by Targeted Magnetic Resonance Imaging Utilizing Conformation-Specific Antibodies against Glycoprotein IIb/IIIa. *J. Vasc. Res.* **46**, 6-14, (2009).

6 von zur Muhlen, C. *et al.* Magnetic Resonance Imaging Contrast Agent Targeted Toward Activated Platelets Allows In Vivo Detection of Thrombosis and Monitoring of Thrombolysis. *Circulation* **118**, 258-267, (2008).

7 Akhtar, A. M. *et al.* *In Vivo* Quantification of Vcam-1 Expression in Renal Ischemia Reperfusion Injury Using Non-Invasive Magnetic Resonance Molecular Imaging. *PLoS One* **5**, e12800, (2010).

8 Hoyte, L. C. *et al.* Molecular magnetic resonance imaging of acute vascular cell adhesion molecule-1 expression in a mouse model of cerebral ischemia. *J. Cereb. Blood Flow Metab.* **30**, 1178-1187, (2010).

9 Serres, S. *et al.* Molecular MRI enables early and sensitive detection of brain metastases. *Proc. Natl. Acad. Sci. USA* **109**, 6674-6679, (2012).

10 McAteer, M. A. *et al.* A Leukocyte-Mimetic Magnetic Resonance Imaging Contrast Agent Homes Rapidly to Activated Endothelium and Tracks With Atherosclerotic Lesion Macrophage Content. *Arterioscler. Thromb. Vasc. Biol.* **32**, 1427-1435, (2012).

11 McAteer, M. A. *et al.* Magnetic Resonance Imaging of Endothelial Adhesion Molecules in Mouse Atherosclerosis Using Dual-Targeted Microparticles of Iron Oxide. *Arterioscler. Thromb. Vasc. Biol.* **28**, 77-83, (2008).

12 von Elverfeldt, D. *et al.* In Vivo Detection of Activated Platelets Allows Characterizing Rupture of Atherosclerotic Plaques with Molecular Magnetic Resonance Imaging in Mice. *PLoS One* **7**, e45008, (2012).

13 Fan, L. M. *et al.* Endothelial Cell-Specific ROS Production Increases Susceptibility to Aortic Dissection. *Circulation*, (2014).

14 von Elverfeldt, D. *et al.* Dual-Contrast Molecular Imaging Allows Noninvasive Characterization of Myocardial Ischemia/Reperfusion Injury After Coronary Vessel Occlusion in Mice by Magnetic Resonance Imaging. *Circulation* **130**, 676-687, (2014).

15 Kanke, M., Simmons, G. H., Weiss, D. L., Bivins, B. A. & Deluca, P. P. Clearance of 141Ce-labeled microspheres from blood and distribution in specific organs following intravenous and intraarterial administration in beagle dogs. *J. Pharm. Sci.* **69**, 755-762, (1980).

16 Ilium, L. *et al.* Blood clearance and organ deposition of intravenously administered colloidal particles. The effects of particle size, nature and shape. *Int. J. Pharm.* **12**, 135-146, (1982).

17 Wunderbaldinger, P., Josephson, L. & Weissleder, R. Tat Peptide Directs Enhanced Clearance and Hepatic Permeability of Magnetic Nanoparticles. *Bioconjugate Chem.* **13**, 264-268, (2002).

18 Chouly, C., Pouliquen, D., Lucet, I., Jeune, J. J. & Jallet, P. Development of superparamagnetic nanoparticles for MRI: effect of particle size, charge and surface nature on biodistribution. *J. Microencapsulation* **13**, 245-255, (1996).

19 Jung, C. W. & Jacobs, P. Physical and chemical properties of superparamagnetic iron oxide MR contrast agents: Ferumoxides, ferumoxtran, ferumoxsil. *Magn. Reson. Imaging* **13**, 661-674, (1995).

20 Shen, T., Weissleder, R., Mikhail Papisov, M., Bogdanov Jr, A. & Brady, T. J. Monocrystalline iron oxide nanocompounds (MION): Physicochemical properties. *Magn. Reson. Med.* **29**, 599-604, (1993).

21 Pittet, M. J., Swirski, F. K., Reynolds, F., Josephson, L. & Weissleder, R. Labeling of immune cells for in vivo imaging using magnetofluorescent nanoparticles. *Nat. Protocols* **1**, 73-79, (2006).
